# CRISPR/Cas9-induced breaks are insufficient to break linkage drag surrounding the ToMV locus of *Solanum lycopersicum*

**DOI:** 10.1093/g3journal/jkaf068

**Published:** 2025-04-02

**Authors:** Jillis Grubben, Gerard Bijsterbosch, Burak Aktürk, Richard G F Visser, Henk J Schouten

**Affiliations:** Plant Breeding, Wageningen University & Research, Droevendaalsesteeg 1, 6708 PB Wageningen, The Netherlands; Graduate School Experimental Plant Sciences, Wageningen University & Research, Droevendaalsesteeg 1, 6708 PB Wageningen, The Netherlands; Plant Breeding, Wageningen University & Research, Droevendaalsesteeg 1, 6708 PB Wageningen, The Netherlands; Plant Breeding, Wageningen University & Research, Droevendaalsesteeg 1, 6708 PB Wageningen, The Netherlands; Plant Breeding, Wageningen University & Research, Droevendaalsesteeg 1, 6708 PB Wageningen, The Netherlands; Plant Breeding, Wageningen University & Research, Droevendaalsesteeg 1, 6708 PB Wageningen, The Netherlands

**Keywords:** CRISPR/Cas9, targeted recombination, *Solanum lycopersicum*, tomato mosaic virus (ToMV) resistance locus, recombination cold spots

## Abstract

Despite the success of CRISPR/Cas9 in inducing DNA double-strand breaks for genome editing, achieving targeted recombination in somatic cells remains challenging, particularly at recombination cold spots like the tomato mosaic virus (ToMV) resistance locus in *Solanum lycopersicum*. We investigated the potential of CRISPR/Cas9-induced targeted recombination in somatic cells to overcome linkage drag surrounding the ToMV locus. We employed two strategies: first, inducing double-strand breaks in both alleles of F_1_ tomato seedlings to promote nonhomologous end joining and homology-directed repair; second, targeting a single allele in a heterozygous background to induce homology-directed repair in seedlings. CRISPR/Cas9 activity was confirmed in F_1_ seedlings by detecting nonhomologous end joining-mediated mutations at the target sites in ToMV. We developed a bioinformatics pipeline to identify targeted recombinants by analyzing SNPs between parental haplotypes, allowing precise tracking of SNP variations. A two-dimensional pooling strategy was employed to distinguish genuine recombination events from PCR artifacts. Despite these advances and the active CRISPR/Cas9 system in F_1_ progeny, no reliable targeted recombinations were found. We extended our research to protoplasts to assess whether CRISPR/Cas9 could induce targeted recombination under different cellular conditions at the same locus. Consistent with our findings in F_1_ plants, we observed no increase in recombinant patterns compared to wild-type controls in protoplasts. Our findings suggest that CRISPR/Cas9-induced DSBs were insufficient to break the genetic linkage at the ToMV locus on chromosome 9 at a detectable level.

## Introduction

DNA recombination and repair mechanisms play a critical role in preserving genomic integrity. A crucial event in the recombination process is the formation of DNA double-strand breaks (DSBs), as these DSBs provide the essential openings needed for the initiation of repair by the recombination machinery (Hustedt and Durocher 2017). DSBs are formed in all cell types throughout their lifespan, but recombination mainly occurs in meiotic cells and rarely in somatic cells (Lang *et al*. 2012; Vrielynck *et al*. 2016; Zelkowski *et al*. 2019). DSBs can result from exposure to external factors such as UV radiation and chemicals and result from internal factors such as DNA replication errors (Kuzminov 2001; Nawkar *et al*. 2013). Although common, DSBs are highly toxic and cause cell death if unrepaired (Nisa *et al*. 2019). Addressing the challenge of inducing targeted recombination in somatic cells of tomato plants using CRISPR/Cas9 technology not only advances tomato breeding but also has the potential to enhance genetic engineering in other crops and species.

To prevent the potentially lethal effects of DSBs, cells are equipped with sophisticated DNA repair mechanisms that ensure genomic stability and proper cellular functioning. These DNA repair mechanisms include rapid nonhomologous end joining (NHEJ) and precise homology-irected repair (HDR) (Siebert and Puchta 2002; Puchta 2005). NHEJ quickly repairs double-strand breaks by directly ligating the DNA ends and employs the Ku70/Ku80 dimer to bind broken ends and recruit DNA Ligase IV to reconnect them (Waterworth *et al*. 2011; Chang *et al*. 2017). The NHEJ repair pathway does not require a homologous template, allowing it to function at all stages of the cell cycle. A drawback of this repair mechanism is the potential for mutations to occur at the site of the DSB (Chang *et al*. 2017). Conversely, HDR uses a similar DNA sequence as a template to repair DSBs. HDR involves strand invasion and DNA synthesis from the homologous sequence to ensure the repair closely matches the original DNA (Siebert and Puchta 2002; Symington and Gautier 2011). However, the effectiveness of HDR is limited to the S and G2 stages of the cell cycle. At these cell cycle stages, the sister chromatids are available and can be used as templates for repair (Puchta 2005). Understanding these repair mechanisms is essential for integrating them with genome editing techniques for precise genetic alterations.

Advancements in genome editing techniques enable precise genetic alterations ranging from local mutations to large-scale genomic reorganizations (Monsur *et al*. 2020; Schwartz *et al*. 2020; Kouranov *et al*. 2022). Initially, only nontargeted mutagenics were applied using ethyl methane sulfonate (EMS) or nuclear radiation. These nontargeted options were complemented by targeted approaches using zinc fingers, transcription activator-like effector nucleases (TALENs), and clustered regularly interspaced short palindromic repeats associated with Cas9 (CRISPR/Cas9) (Menda *et al*. 2004; Osakabe *et al*. 2010; Mussolino *et al*. 2011; Jiang *et al*. 2013). These targeted approaches in directed DSB induction significantly enhance the efficiency and flexibility of genome editing.

Beyond these site-specific targeted approaches, researchers have induced larger genetic alterations through targeted recombination. Targeted recombination often relies on CRISPR/Cas9-induced DSBs to generate specific recombinations, including targeted crossovers or allele replacements at the site of DSB repair (Kouranov *et al*. 2022; Samach *et al*. 2023). The repair pathway chosen for the DSB determines the recombination outcome (Mahapatra and Roy 2020; Ghosh and Raghavan 2021). NHEJ-mediated repair pathway can initiate a crossover directly at the break site, while the HDR-driven recombination pathway enables not only crossover events but also precise allele replacements (Li *et al*. 2018; Filler-Hayut *et al*. 2021; Kouranov *et al*. 2022).

Yelina *et al*. (2022) employed CRISPR/dCas9 to guide the SPO11 complex to specific sites during meiosis, aiming at increasing crossover frequencies at these sites in *Arabidopsis thaliana*. They were unsuccessful in increasing the frequency of crossovers at the target site, highlighting the difficulty of achieving targeted meiotic recombination for crop improvement. Greater progress has been made in inducing recombination in somatic cells by using targeted approaches. Kouranov *et al*. (2022) showed that targeted recombination via NHEJ is possible by inducing DSBs in both alleles of a heterozygous plant. Subsequently, the repair machinery of the cell can erroneously fuse different haplotypes instead of the original DNA strands, resulting in a CRISPR/Cas-induced recombination event. Additionally, HDR-based targeted recombination by cleaving one allele in a heterozygous background was presented (Filler-Hayut *et al*. 2021; Samach *et al*. 2023). Subsequently, the other allele was used as template for repair, which resulted in recombination and allele replacement patterns flanking the DSB. Despite the challenges observed in enhancing meiotic recombination rates, the advancements in CRISPR/Cas9 technology demonstrate the potential of targeted recombination. This leads us to consider how similar targeted recombination approaches could address specific genetic obstacles in agricultural development.

Crucial disease-resistance genes are found in wild relatives of crops. Sometimes these genes reside in recombination cold spots. Recombination cold spots are regions in the genome where genetic recombination occurs less frequently than in other areas. An example is the ToMV resistance gene *Tm-2^2^* in tomato (van Rengs *et al*. 2022). This resistance gene resides in an introgression from *Solanum peruvianum*, covering 79% of Chr 9 in nearly all modern tomato varieties (Schouten *et al*. 2019). Previous studies have identified this region as a recombination cold spot (see Supplementary Fig. 1 for a schematic representation). Viquez-Zamora *et al*. (2014 ) observed no recombination between approximately 8 and 56 Mb on chromosome 9, since the genetic distance remained unchanged despite increasing physical distance. Within this suppressed area lies the ToMV resistance locus, located ∼13.66 Mb in the cv. Moneymaker variety. Additionally, Schouten *et al*. (2019) reported a high frequency of SNPs between 5 and 57 Mbp on chromosome 9, indicating low sequence similarity that likely restricts recombination. Moreover, van Rengs *et al*. (2022) found multiple inversion, translocation, and duplication events in this region when comparing cv. Moneymaker and cv. Moneyberg. The ToMV resistance locus is situated within one of the few syntenic regions shared between these cultivars. Despite five decades of conventional breeding in many breeding programs worldwide, this introgression with *Tm-2^2^* introgression remains unbroken in modern tomato varieties. Therefore, we aimed at overcoming this persistent issue by inducing targeted recombination using CRISPR/Cas.

Given the difficulties in achieving targeted recombination during meiosis (Yelina *et al*. 2022), but success in somatic cells (Filler Hayut *et al*. 2017; Ben Shlush *et al*. 2021; Filler-Hayut *et al*. 2021; Kouranov *et al*. 2022; Samach *et al*. 2023), we focused on somatic cells of young tomato seedlings. We utilized CRISPR/Cas, PacBio sequencing, and a two-dimensional sample pooling system to eliminate false-positive recombination events. Our approach involved the crossing of parental plants harboring either a gRNA, Cas9, or both. We generated F_1_ seedlings from specific crosses and screened them for targeted recombination events. Despite a highly active CRISPR/Cas9 system in the F_1_ offspring, we did not observe an increased recombination incidence compared to the wild type. Our findings suggest that in the recombination cold spot we tested, inducing recombination via CRISPR/Cas9 is insufficient to break linkage drag.

## Materials and methods

### Plant materials

Wild-type and mutant lines of *Solanum lycopersicum* cv. ‘Moneymaker’ and *Solanum lycopersicum* cv. ‘Moneyberg’ were used in this study. Two groups of mutant lines were developed by using *S. lycopersicum* cv. ‘Moneymaker’.

**Fig. 1.**
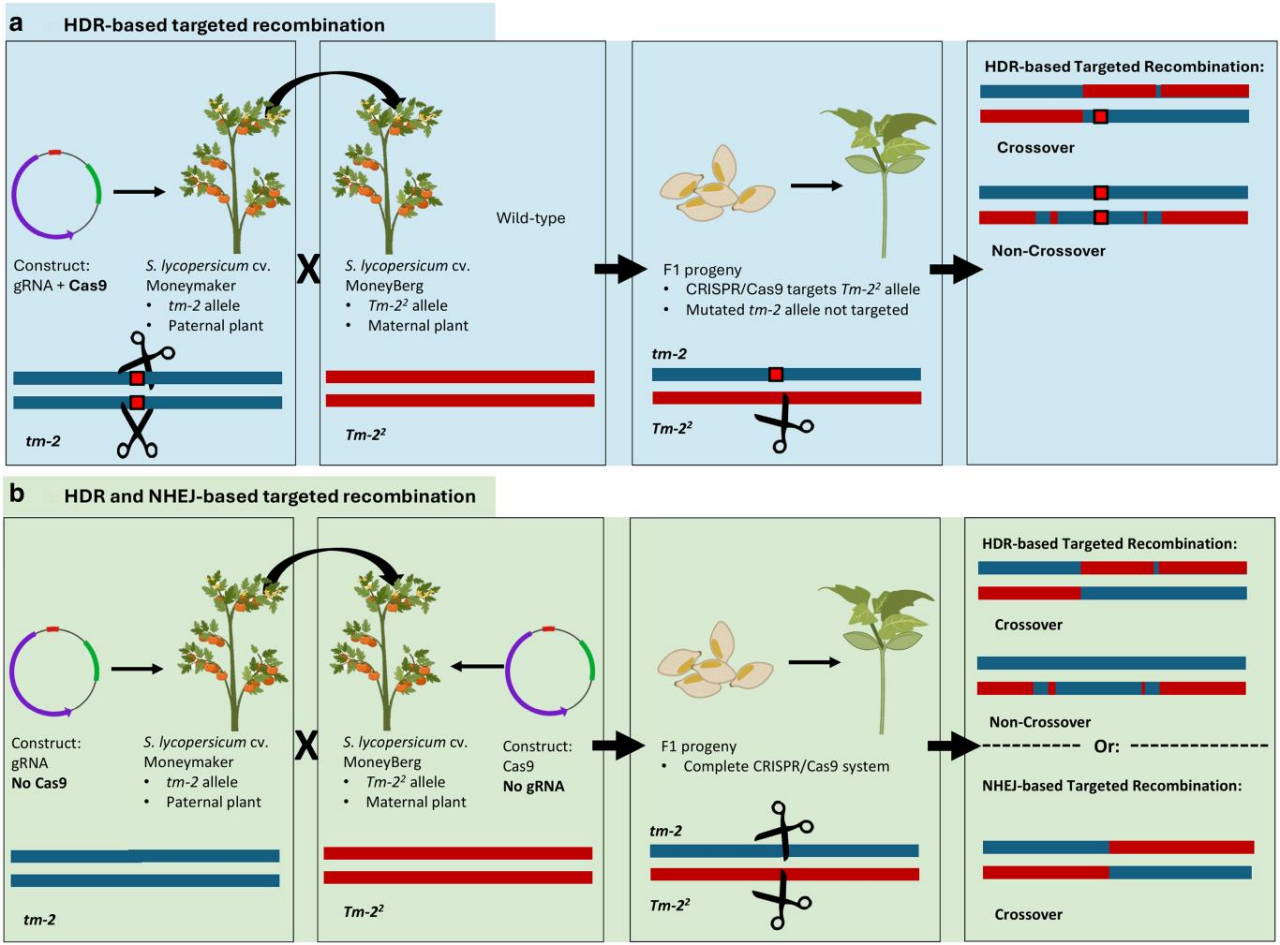
a) Schematic overview of the crossing strategy designed to generate F_1_ progeny with targeted recombination events through HDR only. Left box: *S. lycopersicum* cv. ‘Moneymaker’ T_0_ plants, containing an active CRISPR/Cas9 system, served as pollen donors. These T_0_ plants were specifically selected for having large mutations in both tm-2 alleles (indicated by red blocks on the blue horizontal lines), which disabled CRISPR/Cas9 activity in these mutated tm-2 alleles. Second box: These T_0_ plants were crossed with homozygous *S. lycopersicum* cv. ‘Moneyberg’ wild-type plants, which contained the functional Tm-22 allele. Third box: In the resulting F_1_ progeny that inherited the paternal CRISPR/Cas9 construct, the CRISPR/Cas9 system created double-strand breaks in the maternally inherited Tm-22 allele (indicated by the pair of scissors). Right box: A simplified cartoon depicts potential HDR-mediated repair outcomes, including crossover and noncrossover events. b) HDR and NHEJ-based targeted recombination. Left box: *S. lycopersicum* cv. ‘Moneymaker’ T_0_ plants harbored only a gRNA and no Cas9, and thus had no CRISPR/Cas9 activity. Second box: The maternal *S. lycopersicum* cv. ‘Moneyberg’ plants harbored the Cas9 construct without CRISPR/Cas9 activity either. Third box: Upon crossing, the functional CRISPR/Cas9 system targeted both the tm-2 and Tm-22 alleles in one-quarter of the F_1_ plants. Right box: Possible NHEJ- and HDR-based targeted recombination patterns are shown, including both crossover and noncrossover recombination options. Parts of this figure were produced using BioRender (biorender.com).

The first group of *S. lycopersicum* cv. ‘Moneymaker’ T_0_ plants harbored both a gRNA and the *Cas9* gene and upon crossing with *S. lycopersicum* cv. ‘Moneyberg’ WT plants, targeted recombination could occur via HDR only (Fig. 1a). We selected *S. lycopersicum* cv. ‘Moneymaker’ T_0_ plants that had mutations in both *tm-2* alleles based on mutations that disrupted further CRISPR/Cas9 activity at these sites (Supplementary Fig. 2, a and b) using Tracking of Indels by DEcomposition (Brinkman *et al*. 2014). These T_0_ plants served as pollen donor during crossing with wild-type *S. lycopersicum* cv. Moneyberg plants. We reasoned that upon fertilization of the zygote, the mutated paternal alleles from T_0_ plants would no longer be targeted by CRISPR/Cas9, whereas the maternal *Tm-2^2^* allele from *S. lycopersicum* cv. Moneyberg would be subjected to CRISPR/Cas9 activity. In this scenario, targeted recombination would occur only if the maternal *Tm-2^2^* allele was repaired via HDR using the *tm-2* allele as a template (Fig. 1a). For the first group, one gRNA was used that targeted both the *tm-2* and the *Tm-2^2^* allele (Table 1; Supplementary Table 1; Supplementary Fig. 3).

**Table 1.** Summary of cross descriptions, repair mechanisms, target alleles, gRNAs, and seedling numbers.

Group	Cross description	Repair mechanism	Target allele	gRNA name	Seedlings
1	Moneyberg WT × Moneymaker-CRISPR/Cas9	HDR	*tm-2* and *Tm-2^2^*	gRNA1-HDR	3,888
1	Moneyberg WT × Moneymaker WT	none	none		1,330
2	Moneyberg-Cas9 × Moneymaker-gRNA	HDR or NHEJ	*tm-2* and *Tm-2^2^*	gRNA2-HDR/NHEJ	1,584
2	Moneyberg-Cas9 × Moneymaker-gRNA	HDR	*tm-2*	gRNA3-tm2-HDR	3,760
2	Moneyberg-Cas9 × Moneymaker WT	none	none		1,236

The second group of *S. lycopersicum* cv. ‘Moneymaker’ T_0_ plants contained only the gRNA but lacked the Cas9 gene, resulting in an inactive CRISPR/Cas9 system. *S. lycopersicum* cv. Moneyberg plants harboring the Cas9 component were a kind gift from Dr. R.A. de Maagd, BU Bioscience, Wageningen University & Research. Upon crossing, 1/4th of the progeny inherited both the gRNA as well as the Cas9 construct (Fig. 1b). Progeny that inherited both CRISPR/Cas9 components were subjected to CRISPR/Cas9-mediated double-strand breaks. HDR-based targeted recombination could occur if the paternal *tm-2* allele was repaired using the *Tm-2^2^* allele as a template, or vice versa. NHEJ-based targeted recombination could occur if both alleles were cut in a small window of time, and the homologous fragments were erroneously repaired by fusing noncorresponding ends. In this group of plants, two gRNAs were used. One gRNA targeted both the *tm-2* and *Tm-2^2^* alleles, while the other gRNA specifically targeted only the *tm-2* allele. This specificity was achieved by designing the second gRNA to bind to a position with SNPs. The presence of SNPs at the PAM site in the *Tm-2^2^* allele prevented this gRNA from cutting the *Tm-2^2^* allele, ensuring it only targeted the *tm-2* allele (Table 1; Supplementary Fig. 3). *Solanum pimpinellifolium* G1.1554 plants were used as a control in this study. Plants were cultivated at Unifarm (Wageningen University & Research) at 18–24°C.

### gRNA design, cloning, and in vivo testing of constructs

gRNAs were designed using CRISPOR (http://crispor.tefor.net) and synthesized by Macrogen Europe. GoldenGate cloning (Engler *et al*. 2014 ) was used to generate constructs containing NosP::NPTII, pUBI::Cas9, pU6-26:sgRNA, and pCsVMV::turboGFP (Supplementary Table 2). In constructs containing only the gRNA, an oligo filler replaced the Cas9 position in the plasmid. To identify effective gRNAs, constructs were transfected into F_1_ protoplasts from an *S. lycopersicum* cv. ‘Moneyberg’ × *S. lycopersicum* cv. ‘Moneymaker’ cross, using the protocol by (Maas and Werr 1989) with a 48 h incubation. Transfection efficiency was determined by the fluorescence-to-survival ratio of protoplasts under an Axio Vert.A1 Inverted Microscope (Carl Zeiss). Centrifugation of protoplasts was performed at 150 RCF (Thermo Fisher Scientific Megafuge ST4R Plus-MD). Following centrifugation, the supernatant was discarded, and the remaining protoplasts were immediately flash-frozen in liquid nitrogen for long-term storage. DNA was isolated using the Nucleomag Plant 96 kit (MACHEREY-NAGEL) in combination with the KingFisher Flex System (Thermo Fisher Scientific). The isolated DNA was used as a template to amplify the region around each CRISPR/Cas9 target site using the primers indicated in Supplementary Table 3. Amplicons were sequenced using Hi-Seq sequencing (Eurofins Nederland—Eurofins Scientific). CRISPR/Cas9 mutations in the amplicons were detected using the R package Amplican (Labun *et al*. 2019). The best-performing gRNAs were selected.

### Stable transformation

Tomato plants were stably transformed using an *Agrobacterium*-mediated method adapted from the protocol described by Ellul *et al*. (2003). To ensure a robust selection of T_0_ plants, approximately 800 explants per construct were prepared. This approach enabled the selection of T_0_ plants based on CRISPR-induced mutation profiles. Cotyledons were transversally cut and placed in SIM + AS medium (cocultivation medium) composed of MS salts (4.3 g/l), Nitsch vitamins (108.73 mg/l), sucrose (30 g/l), and micro agar (8 g/l) with a pH adjusted to 5.8, sterilized by autoclaving. Additionally, filter-sterilized zeatin riboside (1.5 mg/l), IAA (0.2 mg/l), and acetosyringone (100 µM) were added post-autoclaving. The explants underwent a 48 h period in the dark at 24°C to promote effective *Agrobacterium* infection in subsequent steps.

*Agrobacterium tumefaciens* strain AGL1 was inoculated into 2 ml of LB medium supplemented with antibiotics and cultured at 28°C for 2 days. After this initial growth phase, 0.5 ml of the culture was diluted into 10 ml of fresh LB medium with antibiotics and incubated overnight at 28°C. The next day, the optical density (OD_600_) of the bacterial cultures was measured, and the bacteria were pelleted by centrifugation. Based on these OD measurements, the pellet was resuspended in approximately 40 ml of inoculation medium (4.4 g/L MS salts and vitamins, 30 g/L glucose, and 100 mM acetosyringone) to achieve a final OD_600_ of 0.4.

Explants were transferred to petri dishes containing inoculation medium composed of MS salts, vitamins (4.4 g/L), glucose (30 g/L), and pH adjusted to 5.2 using 0.1N KOH. Just before use, 50 µl of 100 mM acetosyringone per 50 ml of MS liquid was added to prepare the final *Agrobacterium* suspension. Explants were gently agitated during a 15–20-min incubation to promote infection and then blotted on sterile filter paper to remove excess bacteria before being returned to the preculture plates. After 48 h of cocultivation at 24°C in the dark to promote effective *Agrobacterium* infection, the explants were moved to the selection medium. This medium included MS salts (4.3 g/L), vitamins, sucrose, and micro agar, all sterilized and pH adjusted to 5.8, with added zeatin riboside (1.5 mg/L), IAA (0.2 mg/L), and kanamycin (50 mg/L). Explants were transferred to fresh medium every 3 weeks and monitored for GFP-positive shoots and GFP-negative material was discarded.

### *S*. *lycopersicum* cv. ‘Moneymaker’ T_0_ plant selection

T_0_ plants of *S. lycopersicum* cv. ‘Moneymaker’ were screened for mutations in both alleles that inhibit CRISPR/Cas9 activity at the intended target site. This step was essential to ensure that, after crossing with *S. lycopersicum* cv. ‘Moneyberg’, the resulting F_1_ plants would use only the *S. lycopersicum* cv. ‘Moneyberg’ genome as the template for HDR-mediated targeted recombination. Screening involved DNA extraction from leaves of in vitro-grown *S. lycopersicum* cv. ‘Moneymaker’ T_0_ plants, followed by PCR amplification of the target region. The generated amplicons were subjected to Sanger sequencing and analyzed using TiDE (Brinkman *et al*. 2014) to identify T_0_ plants exhibiting only mutated sequences, not the wild-type (WT) sequence, at the CRISPR/Cas9 cleavage site.

### Genetic crosses

Crosses were made via hand pollination using the *S. lycopersicum* cv. ‘Moneyberg’ as the maternal plant. The experimental setup comprised a control cross between *S. lycopersicum* cv. ‘Moneyberg’ and ‘Moneymaker’ to establish a baseline comparison. Additionally, crosses were performed between *S. lycopersicum* cv. ‘Moneyberg’ and genetically engineered *S. lycopersicum* cv. ‘Moneymaker’ plants, the latter modified to carry the CRISPR/Cas9 system. In a separate set of experiments, ‘Moneyberg’ plants expressing the Cas9 protein were crossed with ‘Moneymaker’ plants containing only a gRNA. We precisely recorded the origin of each seed at the fruit level to trace the source of any putative targeted recombinants. In total, approximately 12,000 seeds were collected from 247 fruits resulting from 46 specific crossing combinations (Supplementary Table 4).

We used three distinct gRNAs capable of binding to either one or both of the ToMV alleles. For the first gRNA, i.e. gRNA1-HDR, we produced stably transformed *S. lycopersicum* cv. Moneymaker T_0_ plants containing Cas9 and this gRNA. Five plants were selected with biallelic mutations at the gRNA1 target site. Due to the mutations, CRISPR/Cas9 was not able to cause DSBs anymore at the gRNA1 target site of these plants. These five plants were crossed with wild-type *S. lycopersicum* cv. Moneyberg plants. In the progeny that harbored CRISPR/Cas9, this construct could only cut in the Moneyberg allele. Therefore, targeted recombination could only be caused by HDR. We sowed F_1_ 3,888 seeds of this type.

For the experiment, we used stably transformed *S. lycopersicum* cv. Moneymaker T_0_ plants with only the gRNA (without Cas9) and crossed them with *S. lycopersicum* cv. Moneyberg plants harboring Cas9. We used two different gRNAs. gRNA2-HDR/NHEJ targeted both alleles, facilitating targeted recombination via either HDR or NHEJ. We sowed 1,584 seeds of this type. The other gRNA, i.e. gRNA3-tm2-HDR, targeted only the *tm-2* allele, enabling targeted recombination via HDR only. We sowed 3,760 seeds of this type (Table 1).

Additionally, over 2,500 control F_1_ seeds were sown (Table 1).

### F_1_ growing conditions

F_1_ heterozygous seeds were collected and labeled, to allow the seeds to be traced to individual fruits. Seeds were sown in 4 × 4 cm rockwool cubes (Grodan) in a climate chamber (Unifarm, Nergena, Wageningen University & Research). *S. pimpinellifolium* seeds were sown at specific positions, enabling verification of correct pooling during NGS analyzes, and for functioning as controls. Seeds underwent cold stratification at 4°C for 3 days, followed by a 14-day germination phase under 16 h light/8 h dark at 24°C day and 18°C night temperatures. After the seedling sampling was completed, the temperature in the climate chamber was adjusted to 12°C. By maintaining this reduced temperature of 12°C throughout the experimental phases—including DNA isolation, PCR amplifications, PacBio sequencing, and subsequent data analysis—the experiment prevented the seedlings from growing excessively large. Maintaining this reduced temperature was critical to facilitate the identification of plants with targeted recombination events. After identification of plants with putative recombination events, the climate conditions were returned to 16 h light/8 h dark at 24°C day and 18°C night temperatures.

### Two-dimensional plant sampling and pooling

A specialized pooling strategy was devised for F_1_ seedlings to determine the authenticity of targeted recombination events and to be able to distinguish them from PCR artifacts. Approximately 12,000 seeds were sown and arranged in a two-dimensional (2D) matrix (Fig. 3, a and b). This matrix consisted of 4 × 4 cm rock wool blocks that were organized in a uniform grid pattern. Each rock wool block contained two seeds, with detailed records maintained for the exact position of each seed, the specific cross, and the fruit from which it was harvested. The matrix was divided into 15 distinct sections, 12 of which comprised 21 × 21 rockwool blocks, while the remaining three sections were configured into 21 × 10 rockwool blocks due to spatial constraints (Fig. 3b).

To generate row-based sample pools, we harvested a 0.5 cm segment from each cotyledon extremity and combined these segments by row. We repeated this for column-based pools, so every seedling contributed to exactly two pools: one row and one column (Fig. 3c). Immediately after sampling, pooled tissues were frozen in liquid nitrogen for DNA extraction.

**Fig. 2.**
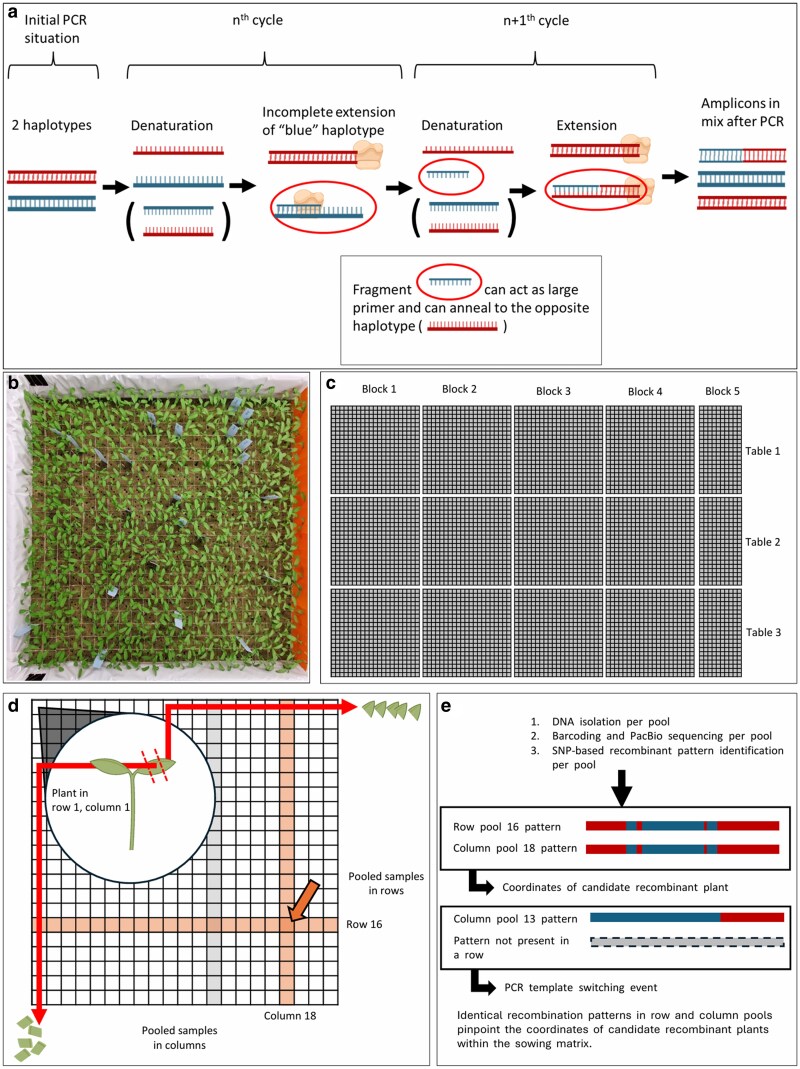
a) Formation of chimeric amplicons due to PCR artifacts. Two gDNA haplotypes (one blue, one red) are shown. After the *n*th cycle, incomplete amplification of the blue template results in a fragment that can act as a large primer in a subsequent cycle, binding to the complementary red sequence and forming a chimeric molecule. b) Block of 21 × 21 rockwool cubes with two seeds sown in each cube. c) Division of rockwool blocks from (b) across three tables, each with four blocks of 21 × 21 cubes and one block of 21 × 10 cubes. d) The 2D pooling method: cotyledons were collected from seedlings in each column and row, sampling each seedling twice. Orange columns and rows represent pools containing a genuine targeted recombination event, indicated by the orange arrow. e) Processing and analysis of pools for chimeric molecules. A genuine recombination event was identified when a pattern appeared in both a column and a row pool within the same block, pinpointing the plant location. Detection of a specific pattern in a single row, but not in the corresponding column or other pools, indicated the presence of a PCR template-switching chimeric molecule. Parts of this figure were produced using BioRender (biorender.com).

This design prevents false positives by cross-verifying each haplotype pattern. Such false-positive events are caused by chimeric DNA molecules generated during PCR, a common issue when working with PCR-based target enrichment of a pool of nonhomozygous starting material (Qiu *et al*. 2001; Haas *et al*. 2011). In such pools, chimeric molecules can form during a PCR cycle when amplicons are partially amplified (Fig. 2a). In subsequent PCR cycles, these partially amplified amplicons of one haplotype can anneal to molecules of the other haplotype. During subsequent cycles, chimeric amplicons are formed that are indistinguishable from actual recombination events.

**Fig. 3.**
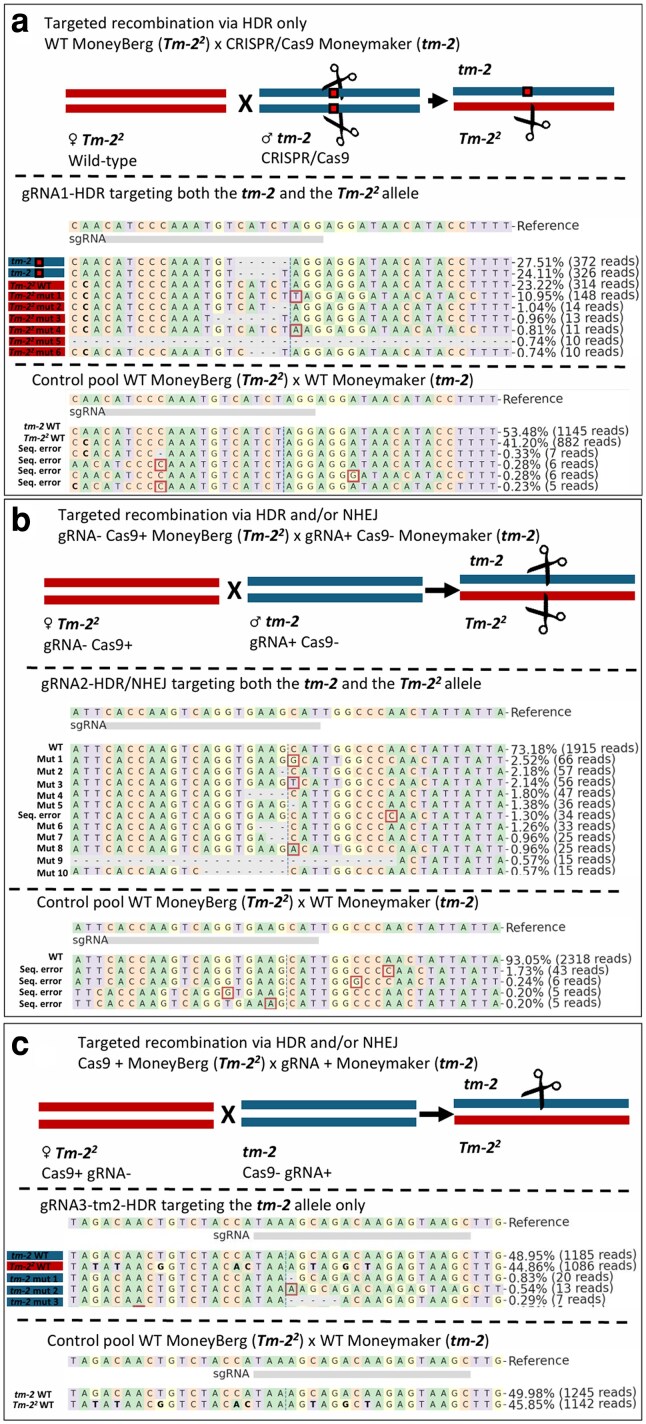
Analysis of PacBio sequencing data using CRISPResso2 for detecting mutations at DSBs in pooled F_1_ seedlings from crosses of Moneyberg (Tm-22) × Moneymaker (tm-2). a) Analysis with gRNA “gRNA1-HDR” targeting tm-2 and Tm-22. The cartoon shows the tm-2 allele of the paternal Moneymaker plant in blue, with the cutting site marked by scissors. Red boxes indicate mutations that prevent further CRISPR/Cas9 cutting in this allele in the F_1_ progeny. The Tm-22 allele of the maternal plant is shown in red, with no cutting indicated due to the absence of CRISPR/Cas9 activity in the wild-type maternal plant. In the F_1_ progeny, new DSBs can be induced in only the Tm-22 allele, indicated with the scissors. Below, CRISPResso2 analysis shows deletions (“-”) and insertions (red boxes), with the predicted cleavage site marked by a vertical dashed line. The sgRNA position is indicated by a gray box. SNPs distinguishing tm-2 from Tm-22 are in bold. Reads of the tm-2 allele are marked in blue and red for Tm-22. Control samples of an F_1_ of WT Moneymaker × WT Moneyberg showed no editing events. b) Analysis with gRNA2-HDR/NHEJ targeting tm-2 and Tm-22. The cartoon shows the tm-2 allele of the paternal plant in blue, with no cutting occurring because it only has the gRNA and not Cas9. The Tm-22 allele of the maternal Moneyberg plant is shown in red, with no cutting indicated as it had Cas9 but not the gRNA. In the F_1_ offspring, plants that inherited Cas9 from the mother and the gRNA from the father can have cutting in both alleles, indicated by scissors. c) Analysis with gRNA3-tm2-HDR targeting tm-2 only. The cartoon shows the tm-2 allele (blue) of the paternal Moneymaker harboring the gRNA but not Cas9 and the Tm-22 allele (red) of the maternal Moneyberg plant with Cas9 but not the gRNA. In F_1_ offspring inheriting Cas9 from the mother and gRNA from the father, cuts are indicated by scissors.

Our goal was to detect and exclude chimeric events by sampling and pooling each seedling twice. Genuine targeted recombination events would display identical SNP patterns in both row and column pools, while patterns unique to a single pool would indicate artifacts generated during PCR (Fig. 3d).

### DNA isolation, PCR, and next-generation sequencing

The frozen pooled samples containing F_1_ cotyledon material were homogenized with a Retsch MM300 Tissue Lyser, and DNA extraction was performed using the NucleoMag Plant kit in tandem with the KingFisher Flex System (Thermo Fisher Scientific). For target locus enrichment, PCR amplification employed PacBio SMRT Sequencing Target-Specific Primers (Supplementary Table 5) and Phire Hot Start II DNA Polymerase (Thermo Fishe Scientific). Each 20 µl PCR reaction was prepared according to the Phire Hot Start II DNA Polymerase guidelines, incorporating 80 ng of genomic DNA. For the detailed PCR protocol please refer to Supplementary Table 5. PCR products were purified using the AMPure XP (Beckman Coulter) kit, adopting an adjusted protocol with an Agencourt AMPure XP solution-to-PCR volume ratio of 0.6:1. The attachment of Barcoded Universal Primers was achieved through a two-cycle Phire II Polymerase protocol (Supplementary Table 6). The purified barcoded amplicon pools were again cleaned using the AMPure XP kit, maintaining the adjusted solution-to-volume ratio. Amplicon fragment sizes were verified on a 1% agarose gel and DNA concentrations of the purified, barcoded amplicon pools were quantified using the Qubit dsDNA HS Assay Kit. Samples were pooled in equimolar concentrations and were sent for PacBio Sequel II SMRT Cell sequencing at the Leiden Genome Technology Center, Leiden University Medical Center.

To verify the targeted recombination patterns, four distinct leaf samples were collected from each of the 10-week-old F_1_ plants. The four collected samples were pooled and processed for sequencing as per the protocol previously described. Sequencing utilized the Oxford Nanopore Technologies GridION system with an R10 flow cell, conducted at the Plant Breeding Department, Wageningen University & Research.

### Targeted recombination pipeline

We developed a Bash and Python-based pipeline for detecting putative targeted recombination events from PacBio or Oxford Nanopore sequencing data. The pipeline allows analysis beyond the tomato ToMV locus through a configuration file where parameters such as the reference genome, SNP positions, and quality thresholds can be specified. The code and detailed documentation are available on Figshare (10.6084/m9.figshare.26582380). A test input file is available for download on Figshare (https://doi.org/10.6084/m9.figshare.27967968.v1) to validate the functionality of the pipeline.

The pipeline was analyzed using ChatGPT-4o (OpenAI, September 2024 iteration) to improve its modularity and reusability. The script was reviewed for functionality (“Please analyze this script. Tell me what it does so that we are on the same page.”), then areas lacking annotation were identified (“Please indicate where potential annotation of the script is missing.”). Suggestions for improving modularity and reusability were requested (“Please think along with me on how to improve script modularity and reusability.”). The responses of the model were reviewed and incorporated into the final code where deemed appropriate.

Prior to running the pipeline, raw sequencing data were demultiplexed to separate each sample pool into individual files corresponding to the row or column pools in the 2D sampling matrix. Seqtk (https://github.com/lh3/seqtk) was then used to convert sequencing data from FASTQ format to FASTA format. To effectively manage and trace each read in subsequent steps in the pipeline, a pool identifier was appended to each read name. This was accomplished using set (https://github.com/mirror/sed). This modification allowed us to identify the source pool for each read in each modified FASTA file. The modified reads were then concatenated into a master file, serving as the input for the pipeline.

The pipeline consists of several key steps. First, the concatenated reads were aligned to the tm-2 reference genome using Minimap2 (https://github.com/lh3/minimap2). The alignment files were then converted, sorted, and indexed using SAMtools (https://github.com/samtools/samtools) to prepare them for further analysis. Next, a custom Python script (concatenated_snp_string_from_bam_v1.py) was used to extract SNP positions from each read in the BAM file, generating SNP strings representing the sequence variations at specified loci. This step required three key inputs: the BAM file with aligned reads, an SNP BED file detailing SNP positions, and a target BED file listing regions of interest.

Next, a custom Python script (haplotype_script_excl_position.py) converted the SNP strings into clearly distinguishable haplotype strings by renaming bases according to their specific alleles. In the resulting output file, bases were labeled as “M” for the *tm-2* allele from *S. lycopersicum* cv. ‘Moneymaker’ and “B” for the *Tm-2^2^* allele from *S. lycopersicum* cv. ‘Moneyberg.’ Deletions in the sequences were marked with “-”, and bases that did not correspond to either the M or B haplotypes were annotated with “?”.

Recombinant sequences were identified and extracted from the haplotype data by analyzing sequences for the presence of recombinant haplotypes characterized by interspersed “M” and “B” alleles, representing different parental origins. Only sequences displaying more than one instance of each allele type were considered recombinants, excluding sequences predominantly consisting of a single allele type. These identified recombinant reads were saved for further analysis, enabling the identification of chimeric sequences within the haplotype data.

Following the extraction of recombinant sequences, a subsequent Python script (group_recombinants.py) was employed to group identical recombinant reads based on their sequences and associated pool identifiers. The groups were renamed to reflect the pool and the frequency of occurrence. Only groups with more than two reads and fewer than 15 unidentified bases (“?”) were retained for further analysis. The outputted recombinant patterns were then manually compared between rows and pools to verify putative targeted recombinant events.

### CRISPR/Cas9 editing efficiency testing

CRISPR/Cas9-induced mutations were quantified using the CRISPResso2 software package, using the “CRISPRessoBatch” pipeline (Clement *et al*. 2019). This pipeline was used to analyze PacBio Sequel II data from F_1_ seedling pools, F_1_ protoplast pools, and ONT sequencing data of mature F_1_ plants.

### Generation and transfection of F_1_ protoplasts

We generated protoplasts from seedlings of *S. lycopersicum* cv. Moneyberg × *S. lycopersicum* cv. Moneymaker, which shares the same genotype as the WT F_1_ plants used in the F_1_ seedling experiment. The seeds were first sterilized in 1% NaClO for 20 min and subsequently washed with sterilized Milli-Q. Seeds were sown on a germination medium (½ MS including Duchefa vitamins, 3% sucrose, and 0.8% Daishin agar, pH = 5.8). Seeds were sown in plastic vessels (OS140BOX/green filter, Duchefa) and subsequently were grown for 3 weeks in a climate chamber (24°C, 60% relative air humidity, and light intensity of 150 Wm^2^). Protoplast generation, isolation, and transfection were carried out following the protocol described in our previous study, available as a preprint on bioRxiv (Grubben *et al*. 2024).

## Results

### Approaches developed for achieving targeted recombination via NHEJ or HDR

Our research aimed to induce targeted recombination events in *S. lycopersicum* by inducing DSBs in the ToMV resistance locus on chromosome 9 using CRISPR/Cas9. The tomato plants were heterozygous for this locus, having the functional *Tm-2^2^* allele conferring resistance and the nonfunctional allele *tm-2*. We focused on two repair mechanisms: HDR-based repair only and a combination of HDR-based and NHEJ-based repair. To induce HDR-based targeted recombination, we transformed *S. lycopersicum* cv. ‘Moneymaker’ plants that were homozygous for *tm-2* with CRISPR/Cas9 constructs targeting both the *tm-2* alleles (Fig. 1a). T_0_ plants with biallelic mutations in the *tm-2* allele were selected and were crossed with wild-type *S. lycopersicum* cv. ‘Moneyberg’ plants carrying the *Tm-2^2^* allele homozygously. The CRISPR/Cas construct could cut that allele too. In the offspring, the CRISPR/Cas construct could not cut in the mutated *tm-2* allele anymore but still could target the *Tm-2^2^* allele. This could induce targeted recombination via HDR-based repair of the *Tm-2^2^* allele using the mutated *tm-2* allele as a template.

Additionally, we developed by means of stable transformation *S. lycopersicum* cv. ‘Moneymaker’ plants containing only the gRNA but lacking the Cas gene. We crossed these transgenic plants with cv. ‘Moneyberg’ plants harboring the Cas9 gene but lacking the gRNA. One-quarter of the offspring harbored both the gRNA and Cas9, being able to cut both in *tm-2* and *Tm-2^2^*. We aimed at targeted recombination via HDR-based or NHEJ-based events in these seedlings (Fig. 1b). By comparing these two approaches, we sought to elucidate the efficiency and nature of targeted recombination mechanisms in tomato.

In view of the anticipated rarity of targeted recombination events, we conducted 247 crosses, and we sowed over 9,000 F_1_ seeds to induce targeted recombination via either HDR or NHEJ, using three different gRNAs. Additionally, we sowed more than 2,500 seeds as controls (Table 1).

### Implementation of a 2D pooling strategy enables differentiation of chimeric PCR artifacts from true recombinants

We used a 2D pooling strategy to distinguish genuine recombination events from chimeric molecules due to PCR artifacts. Such chimeric molecules that can be introduced during PCR amplification of polyallelic material are indistinguishable from genuine targeted recombination events. We aimed to distinguish PCR artifacts from genuine targeted recombination events by sampling each seedling twice. To achieve this, we pooled samples from each seedling according to their row and column positions within a grid-like matrix. This dual pooling strategy allowed us to identify genuine recombination events by comparing SNP patterns across row and column pools, as chimeric molecules generated during PCR would likely not show the same pattern in both row and column pools (Fig. 2, b–d). A second advantage of this pooling strategy was that less sequencing samples were required compared to sequencing each individual seedling twice.

To validate our targeted recombinant detection system, we utilized *S. pimpinellifolium* seedlings as a positive control. These seedlings were distributed across the experimental sowing blocks and displayed a distinct SNP pattern combining alleles from both Moneymaker and Moneyberg. These SNP patterns are comparable to HDR-based repair patterns that could be induced by our targeted recombination method and should therefore be detectable in our targeted recombinant detection pipeline (Supplementary Table 7). As expected, the expected *S. pimpinellifolium* SNP combinations were identified in the sequencing data from our F_1_ pools. Furthermore, the analysis confirmed that the positive control SNP patterns accurately corresponded to the coordinates of the original *S. pimpinellifolium* seed locations.

We analyzed the PacBio sequencing data from these sampling pools to identify SNP patterns indicative of recombination events. We compared the SNP patterns in the row and column pools to distinguish genuine recombination events from PCR artifacts. Genuine recombination events were identified by identical SNP patterns present in both pools, whereas patterns unique to either the row or column pools suggested the presence of chimeric molecules caused by PCR template switching events (Fig. 2e).

### Active CRISPR/Cas9-induced mutations confirmed in F_1_ seedlings

We first confirmed CRISPR/Cas9-induced mutations in pooled F_1_ seedlings from specific parental crosses to ensure the CRISPR/Cas9 system functioned in the F_1_ progeny. We screened the PacBio sequencing data of these pooled seedlings for mutations at the expected DSB sites. Based on SNP patterns, we could distinguish the maternal ‘Moneyberg’ alleles from the paternal ‘Moneymaker’ alleles (Supplementary Fig. 4). In pools of F_1_ plants from wild-type ‘Moneyberg’ crossed with ‘Moneymaker’ containing Cas9 and gRNA1-HDR, we found the expected mutations in the paternal *tm-2* allele. These mutations, which prevented further CRISPR/Cas9 activity in the *tm-2* allele, were only detected in the paternal DNA. As expected, we also found mutations in the maternally transferred *Tm-2^2^* allele, indicating active CRISPR/Cas9 targeting of this allele (Fig. 3a). We observed no CRISPR/Cas9 activity in the control pools (Supplementary Table 8; Supplementary Fig. 5a).

Similarly, we observed new mutations at the expected DSB sites in pools of F_1_ seedlings from crosses between ‘Moneyberg’ (containing the Cas9 gene) and ‘Moneymaker’ (containing either gRNA2-HDR/NHEJ or gRNA3-tm2-HDR, but lacking Cas9). The parental plants of these F_1_ seedlings lacked a complete CRISPR/Cas9 system and could not induce mutations themselves. In pools with gRNA2-HDR/NHEJ, which targeted both alleles, we detected mutations in both alleles as expected (Fig. 3b). Mutation frequencies varied but clustered ∼20–30% (Supplementary Table 8; Supplementary Fig. 5b). Although at lower frequencies, we also observed mutations with gRNA3-tm2-HDR, which targets only the *tm-2* allele (Supplementary Table 8; Supplementary Fig. 5c). The observed mutations occurred exclusively in the *tm-2* allele, as expected (Fig. 3c). These findings confirm the successful initiation of the CRISPR/Cas9 system in F_1_ progeny derived from parental plants that contained only part of the CRISPR/Cas9 system.

We identified microhomology-mediated end joining (MMEJ) repair signatures at the CRISPR/Cas9 target sites (Supplementary Fig. 6). MMEJ-based repair was observed at the gRNA1-HDR and gRNA2-HDR/NHEJ target sites, whereas no MMEJ-mediated repair was detected at the gRNA3-tm2-HDR target site.

### Targeted recombination screening in F_1_ pools yielded inconclusive results

**Fig. 4.**
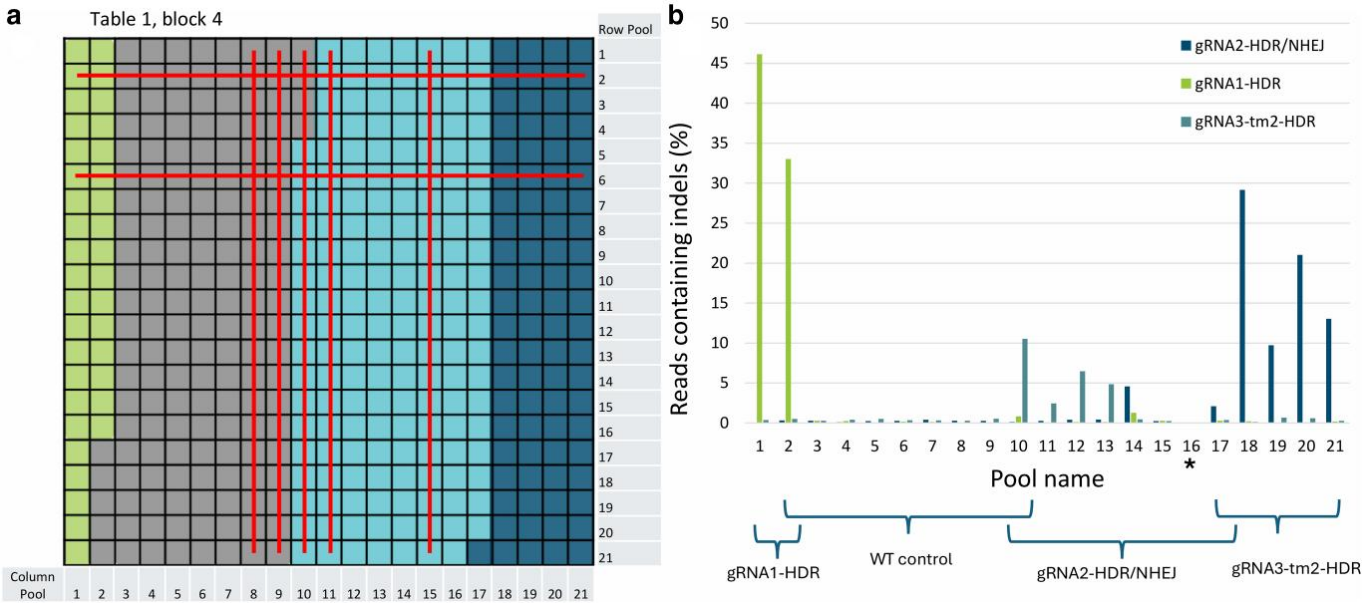
a) The sowing block consisting of 21 × 21 rockwool pieces shows putative targeted recombination events in pooled samples. Pooled row samples are indicated on the right, and pooled column samples are indicated at the bottom. Horizontal and vertical red lines represent rows and columns where putative targeted recombinants were detected, respectively. Seedlings with gRNA1-HDR are shown in green, gRNA2-HDR/NHEJ in dark blue, gRNA3-tm2-HDR in light blue, and control seedlings in gray. b) Shows on the *y*-axis the percentage of reads containing CRISPR/Cas9-induced indels at the DSB sites for the three aforementioned gRNAs. The *y*-axis shows the percentage of reads containing CRISPR/Cas9-induced indels at the DSB sites for the three gRNAs. The *x*-axis displays column pools 1–21. Curly brackets indicate the gRNAs expected to induce mutations in each column pool. Note that column pool 16* had insufficient sequencing depth due to a technical error and thus shows no mutations.

The newly formed mutations in our sequencing pools confirmed that our CRISPR/Cas9 system worked as intended and gave us confidence that our experimental setup was a strong basis for the generation of targeted recombinants. We used our in-house designed targeted recombination pipeline to analyze the PacBio sequencing data from the row and column pools of each 2D pooling block. We discarded chimeric patterns present only in a single row or column pool and retained patterns occurring in both row and column pools to eliminate chimeric molecules formed by PCR template switching (Fig. 2, d and e). We detected putative targeted recombination events that occurred in both row and column pools (Fig. 4a, Table 2). Surprisingly, all detected recombination events were confined to the fourth block on the first table (Fig. 2c). This was unexpected since we randomly sowed F_1_ seeds from different crosses, including controls, across the 2D pooling blocks (Fig. 2c, Supplementary Table 7). Given this distribution, we expected the putative recombination events to occur in various blocks rather than clustering in one.

**Table 2.** Putative targeted recombination patterns detected in both row and column pools, their frequencies, and CRISPR/Cas9-induced indel percentages.

Pool name	Putative targeted recombination pattern^a^	Pattern frequency (%)	Indels at target site (%)	Editing at gRNA target
Column Pool 8	BBBMMMMMMMMMMMMMMMMMMMMMMMMMMMMMMMMMMMMMMMMMMMMMMMMMMMMMMMMMMM	0.24	0	Negative control
Column Pool 9	MMMMMMMBBBBBBBBBBBBBBBBBBBBBBBBBBBBBBBBBBBBBBBBBBBBBBBBBBBBBBB	0.39	0.3	Negative control
	BBBBBBBMMMMMMMMMMMMMMMMMMMMMMMMMMMMMMMMMMMMMMMMMMMMMMMMMMMMMMM	0.52		
	BBBBBBBBBMMMMMMMMMMMMMMMMMMMMMMMMMMMMMMMMMMMMMMMMMMMMMMMMMMMMM	0.52		
	MMMMMMMMMMMMMMMMMMMMBBBBBBBBBBBBBBBBBBBBBBBBBBBBBBBBBBBBBBBBBB	0.39		
	MMMMMBBBBBBBBBBBBBBBBBBBBBBBBBBBBBBBBBBBBBBBBBBBBBBBBBBBBBBBBB	0.52		
	BBBMMMMMMMMMMMMMMMMMMMMMMMMMMMMMMMMMMMMMMMMMMMMMMMMMMMMMMMMMMM	0.91		
Column Pool 10	MMMMMMMBBBBBBBBBBBBBBBBBBBBBBBBBBBBBBBBBBBBBBBBB**|**BBBBBBBBBBBBBB	0.21	10.5	gRNA3-tm2-HDR
	BBBBBBBMMMMMMMMMMMMMMMMMMMMMMMMMMMMMMMMMMMMMMMMM**|**MMMMMMMMMMMMMM	0.21		
	MMMMMMMMMBBBBBBBBBBBBBBBBBBBBBBBBBBBBBBBBBBBBBBB**|**BBBBBBBBBBBBBB	0.58		
	BBBBBBBBBMMMMMMMMMMMMMMMMMMMMMMMMMMMMMMMMMMMMMMM**|**MMMMMMMMMMMMMM	0.41		
	MMMMMMMMMMMMMMMMMMMMMBBBBBBBBBBBBBBBBBBBBBBBBBBB**|**BBBBBBBBBBBBBB	0.12		
	MMMMMMMMMMBBBBBBBBBBBBBBBBBBBBBBBBBBBBBBBBBBBBBB**|**BBBBBBBBBBBBBB	0.12		
	MMMMMMMMMMMMMMMMMMMMMMBBBBBBBBBBBBBBBBBBBBBBBBBB**|**BBBBBBBBBBBBBB	0.17		
	MMMMMMMMMMMMMMMMMMMMMMMBBBBBBBBBBBBBBBBBBBBBBBBB**|**BBBBBBBBBBBBBB	0.12		
	MMMMMBBBBBBBBBBBBBBBBBBBBBBBBBBBBBBBBBBBBBBBBBBB**|**BBBBBBBBBBBBBB	0.17		
	BBBBBBBBBBBBBMMMMMMMMMMMMMMMMMMMMMMMMMMMMMMMMMMM**|**MMMMMMMMMMMMMM	0.12		
	BBBMMMMMMMMMMMMMMMMMMMMMMMMMMMMMMMMMMMMMMMMMMMMM**|**MMMMMMMMMMMMMM	0.33		
Column Pool 15	MMMMMMMBBBBBBBBBBBBBBBBBBBBBBBBBBBBBBBBBBBBBBBBB**|**BBBBBBBBBBBBBB	0.14	0.2	gRNA3-tm2-HDR
	BBBBBBBMMMMMMMMMMMMMMMMMMMMMMMMMMMMMMMMMMMMMMMMM**|**MMMMMMMMMMMMMM	0.18		
	MMMMMMMMMBBBBBBBBBBBBBBBBBBBBBBBBBBBBBBBBBBBBBBB**|**BBBBBBBBBBBBBB	0.37		
	BBBBBBBBBMMMMMMMMMMMMMMMMMMMMMMMMMMMMMMMMMMMMMMM**|**MMMMMMMMMMMMMM	0.55		
	MMMMMMMMMMMMMMMMMMMMBBBBBBBBBBBBBBBBBBBBBBBBBBBB**|**BBBBBBBBBBBBBB	0.18		
	MMMMMMMMMMBBBBBBBBBBBBBBBBBBBBBBBBBBBBBBBBBBBBBB**|**BBBBBBBBBBBBBB	0.14		
	MMMMMMMMMMMMMMMMMMMMMMBBBBBBBBBBBBBBBBBBBBBBBBBB**|**BBBBBBBBBBBBBB	0.23		
	MMMMMMMMMMMMMMMMMMMMMMMBBBBBBBBBBBBBBBBBBBBBBBBB**|**BBBBBBBBBBBBBB	0.23		
	BBBMMMMMMMMMMMMMMMMMMMMMMMMMMMMMMMMMMMMMMMMMMMMM**|**MMMMMMMMMMMMMM	0.41		
Row Pool 2	MMMMMMMBBBBBBBBBBBBBBBBBBBBBBBBBBBBBBBBBBBBBBBBBBBBBBBBBBBBBBB	0.12	^b^	^b^
	MMMMMMMMMBBBBBBBBBBBBBBBBBBBBBBBBBBBBBBBBBBBBBBBBBBBBBBBBBBBBB	0.12		
	MMMMMMMMMMMMMMMMMMMMMBBBBBBBBBBBBBBBBBBBBBBBBBBBBBBBBBBBBBBBBB	0.12		
	MMMMMMMMMMMMMMMMMMMMBBBBBBBBBBBBBBBBBBBBBBBBBBBBBBBBBBBBBBBBBB	0.12		
	MMMMMMMMMMBBBBBBBBBBBBBBBBBBBBBBBBBBBBBBBBBBBBBBBBBBBBBBBBBBBB	0.12		
	MMMMMMMMMMMMMMMMMMMMMMBBBBBBBBBBBBBBBBBBBBBBBBBBBBBBBBBBBBBBBB	0.12		
	MMMMMMMMMMMMMMMMMMMMMMMBBBBBBBBBBBBBBBBBBBBBBBBBBBBBBBBBBBBBBB	0.16		
	MMMMMBBBBBBBBBBBBBBBBBBBBBBBBBBBBBBBBBBBBBBBBBBBBBBBBBBBBBBBBB	0.28		
Row Pool 6	MMMMMMMBBBBBBBBBBBBBBBBBBBBBBBBBBBBBBBBBBBBBBBBBBBBBBBBBBBBBBB	0.22	^b^	^b^
	BBBBBBBMMMMMMMMMMMMMMMMMMMMMMMMMMMMMMMMMMMMMMMMMMMMMMMMMMMMMMM	0.29		
	MMMMMMMMMBBBBBBBBBBBBBBBBBBBBBBBBBBBBBBBBBBBBBBBBBBBBBBBBBBBBB	0.44		
	BBBBBBBBBMMMMMMMMMMMMMMMMMMMMMMMMMMMMMMMMMMMMMMMMMMMMMMMMMMMMM	0.22		
	MMMMMMMMMMMMMMMMMMMMMBBBBBBBBBBBBBBBBBBBBBBBBBBBBBBBBBBBBBBBBB	0.22		
	MMMMMMMMMMMMMMMMMMMMBBBBBBBBBBBBBBBBBBBBBBBBBBBBBBBBBBBBBBBBBB	0.22		
	MMMMMMMMMMBBBBBBBBBBBBBBBBBBBBBBBBBBBBBBBBBBBBBBBBBBBBBBBBBBBB	0.22		
	MMMMMMMMMMMMMMMMMMMMMMBBBBBBBBBBBBBBBBBBBBBBBBBBBBBBBBBBBBBBBB	0.29		
	MMMMMMMMMMMMMMMMMMMMMMMBBBBBBBBBBBBBBBBBBBBBBBBBBBBBBBBBBBBBBB	0.37		
	MMMMMBBBBBBBBBBBBBBBBBBBBBBBBBBBBBBBBBBBBBBBBBBBBBBBBBBBBBBBBB	0.51		
	BBBBBBBBBBBBBMMMMMMMMMMMMMMMMMMMMMMMMMMMMMMMMMMMMMMMMMMMMMMMMM	0.22		
	BBBMMMMMMMMMMMMMMMMMMMMMMMMMMMMMMMMMMMMMMMMMMMMMMMMMMMMMMMMMMM	0.22		

^a^The symbol “|” indicates the SNP positions between which the CRISPR/Cas9 system was predicted to make cuts.

^b^Row pools include F_1_ seedlings from wild-type and CRISPR/Cas9-edited plants with different gRNA targets (Fig. 4a), rendering the indel frequency in these pools non-informative.

We analyzed F_1_ progeny obtained from crosses involving specific genotypes and control groups to investigate the occurrence and distribution of putative recombination patterns. The recombination patterns were identified in F_1_ seedlings resulting from crosses between *S. lycopersicum* cv. ‘Moneyberg’ containing Cas9 and *S. lycopersicum* cv. ‘Moneymaker’ lacking Cas9 but containing gRNA3-tm2-HDR (Fig. 4a, Table 2). These F_1_ seedlings could harbor targeted recombination events occurring solely via HDR, as the gRNA3-tm2-HDR was designed to target only the *tm-2* allele (Supplementary Table 1). HDR-based repair often results in recombination patterns that do not occur at the CRISPR/Cas9 cutting site but can form away from the DSB site based on the resectioning length of the 5′-end at the DSB site (Schmidt *et al*. 2019), which was consistent with our observations (Table 2). However, similar recombination patterns were also detected in control pools comprising WT *S. lycopersicum* cv. ‘Moneyberg’ crossed with WT *S. lycopersicum* cv. ‘Moneymaker’ (Fig. 4a, Table 2). To rule out the possibility of mislabeling, we confirmed the absence of CRISPR/Cas9 editing in these control pools (Fig. 4b). Additionally, we verified CRISPR/Cas9 activity in experimental pools containing putative recombination patterns by screening for DSB site mutations, where we identified NHEJ-based mutations likely unrelated to targeted recombination (Fig. 4b, Supplementary Table 8). Consequently, since similar recombination events occurred in control pools lacking CRISPR/Cas9 activity, we cannot conclusively confirm targeted recombination events. Therefore, additional analyses were necessary to validate and clarify these findings. The 2D pools that consisted of crosses containing the gRNA1-HDR and gRNA2-HDR/NHEJ did not contain targeted recombination events, but they did show high mutation rates at the DSB sites (Fig. 4b).

We included *S. pimpinellifolium* plants as positive controls in each 2D pooling block. These control plants exhibited distinct and recognizable haplotype patterns when their amplicons were processed through our targeted recombination detection pipeline (see Supplementary Table 7 for details). In pools containing the homozygous *S. pimpinellifolium* genotypes, the median read depth for these haplotype patterns was 70 reads (Supplementary Fig. 7).

If the targeted recombination patterns in *S. lycopersicum* were heterozygous, the expected read depth for targeted recombinant events would be approximately 35 reads, assuming the event occurred consistently throughout the entire plant. However, the putative recombination patterns appeared at a depth of 3–14 reads (Supplementary Table 9). This lower read depth might be attributed to multiple distinct recombination patterns or chimeric template switching artifacts. Consistently, we detected multiple distinct putative recombination events in both row and column pools with putative targeted recombinations (Fig. 4a, Table 2). These recombination patterns were not unique to one column/row combination but appeared in multiple row/column combinations.

Additionally, we observed complement haplotype sequences in different column/row pool combinations. For example, sequences such as “…MMMMBBB…” and “…BBBBBMMM…” can indicate genuine recombination patterns where there is an exchange between the alleles in the F_1_ plant.

We also examined the other 2D pooling blocks to assess whether any single-pool chimeric molecules (i.e. appearing exclusively in either a row or a column pool) were abundant, as a high frequency of such events could increase the likelihood of accidentally generating the same chimeric pattern in both technical replicates. In other words, if template switching artifacts were common, then by chance alone, a spurious recombinant-like pattern might appear in both a row and a column pool, potentially mimicking a genuine recombination event. However, in these additional blocks, we detected only a small number of single-pool chimeric molecules, and none of these appeared in both row and column pools within the same block. This suggests that PCR template switching rarely produced artifact patterns in two separate technical replicates, reinforcing that our dual pooling strategy was effective for distinguishing true recombinants from chimeric PCR artifacts.

Out of the 555 pools analyzed, only 16 contained a small number of chimeric molecules, with artifact prevalence ranging from 0.09 to 2.7% (Supplementary Table 10). While these events were likely chimeric sequences resulting from PCR template switching, we cannot exclude the possibility that they might represent genuine rare targeted recombination events. If targeted recombination occurred in a limited region of the cotyledon, the small number of affected cells could explain the lower-than-expected read depth observed for these events. Additionally, because each seedling was sampled twice, once for the row pool and once for the column pool, targeted recombination events confined to a small area might be detected in only one pool, preventing their simultaneous identification in both pools.

### Targeted recombination events were not confirmed in mature F_1_ seedlings

To validate the putative recombinations and to evaluate whether targeted recombination patterns manifested only during later developmental stages, we cultivated 182 F_1_ seedlings to maturity over 4 months.

These seedlings, with potential CRISPR/Cas9-mediated recombination, originated from the 2D sowing block containing putative targeted recombinant seedlings. We selected GFP-positive seedlings from the sowing block that showed the most putative recombination events. We reasoned that if we found the same patterns in these mature F_1_ plants as in the pooled row and column samples, this would indicate genuine recombination events. We collected five distinct leaf samples from each F_1_ plant. These samples were sequenced using ONT sequencing, and we analyzed the targeted recombination patterns using our recombination detection pipeline. We found that over 97% of the putative recombination patterns in mature plants matched those in the pooled F_1_ plants. We expected to find F_1_ plants with these exact patterns at the coordinates where the corresponding row and column pools intersected.

We identified two F_1_ plants in which both row and column pools displayed putative targeted recombination patterns at their intersection coordinates. The first plant (Column 15, Row 2) originated from a cross between a ‘Moneyberg’ maternal plant (harboring Cas9) and a paternal plant carrying gRNA3-tm2-HDR but lacking Cas9. Eight distinct putative recombination patterns, including complement haplotypes, were detected, mirroring those found in the corresponding column and row pools. The second plant (Column 9, Row 2) was a control derived from a wild-type ‘Moneyberg’ crossed with wild-type ‘Moneymaker,’ yet it exhibited three putative recombination patterns (without any complementary haplotypes).

Despite these patterns appearing in both row and column pools for each plant, both plants showed low read depth for these patterns compared to the total sequencing depth. For instance, the first plant had ∼15,000 reads total, but only 10–38 reads displayed the recombination patterns, and the second plant had 23,000 reads with only 16–24 matching these events. The appearance of similar low-frequency recombination patterns in a control plant suggests that these signals are PCR-generated chimeric artifacts rather than genuine targeted recombination. Even if targeted recombination had occurred in only a small subset of cells, the fact that a control plant (lacking the CRISPR/Cas9 system) also exhibited similar patterns makes it unlikely that these represented true recombination events. Nonetheless, we cannot entirely rule out the possibility that one or more events in the first plant may have been genuine targeted recombination events.

Notably, several seedlings in the sowing block where chimeric molecules were detected showed signs of damping-off disease. This was particularly evident in the top section of the block, corresponding to the coordinates where the chimeric patterns were found. Damping-off is a common disease in seedlings caused by soil-borne pathogens, leading to rapid and severe seedling decay (Lamichhane *et al*. 2017). Symptoms were mild in the seedlings during the sampling stage, but the disease led to the loss of several mature plants that were kept for targeted recombinant verification.

### Protoplast experiments confirm lack of increased targeted recombination at the ToMV locus

We were unable to reliably confirm targeted recombinants in F_1_ seedlings and mature F_1_ plants, possibly due to the extreme rarity of such events, insufficient seedling numbers, or chimeric recombination patterns. Alternatively, our gRNAs might have targeted sequence motifs within the ToMV locus that did not allow for targeted recombination-based repair via NHEJ or HDR. To address this, we conducted a protoplast experiment using heterozygous protoplasts identical in genotype to the Moneyberg × Moneymaker genotype used in the F_1_ seedlings screening. We transfected these protoplasts with CRISPR/Cas9 constructs, including the three gRNAs from the F_1_ seedling experiment (gRNA1-HDR, gRNA2-HDR/NHEJ, and gRNA3-tm2-HDR) (Table 1) and four additional gRNAs (Table 3). Each construct was tested in four biological replicates, while the controls were tested in eight biological replicates. Three of the additional gRNAs cut both the Moneyberg *Tm-2^2^* and Moneymaker *tm-2* alleles, while one cut only the Moneymaker *tm-2* allele.

**Table 3.** Overview of gRNA names, repair mechanisms, and target alleles.

gRNA name	Repair mechanism	Target allele
gRNA1-HDR/NHEJ (771)^a^	HDR or NHEJ	*tm-2* and *Tm-2^2^*
gRNA2-HDR/NHEJ (1001)	HDR or NHEJ	*tm-2* and *Tm-2^2^*
gRNA3-tm2-HDR (tmv3)	HDR	*tm-2*
gRNA4-tm2-HDR (tmv1)	HDR	*tm-2*
gRNA5-HDR/NHEJ (1352)	HDR or NHEJ	*tm-2* and *Tm-2^2^*
gRNA6-HDR/NHEJ (1838)	HDR or NHEJ	*tm-2* and *Tm-2^2^*
gRNA7-HDR/NHEJ (2038)	HDR or NHEJ	*tm-2* and *Tm-2^2^*
gRNA-control	none	none

^a^The gRNA1-HDR/NHEJ targets both alleles in a WT heterozygous F_1_ background, allowing for HDR and NHEJ-based recombination. In the F_1_ seedlings experiment, this gRNA could only induce HDR-based recombination because the *tm-2* allele inherited from the paternal plant was already mutated, lacking the complete target sequence necessary for NHEJ-based recombination.

We sequenced the ToMV allele and analyzed protoplast pools via PacBio Sequel II sequencing using the same primers as in the F_1_ seedling experiment. We screened approximately 150,000 genomes per sample for putative targeted recombinants using our bioinformatics pipeline. Screening this many genomes per sample means each sequence likely has a depth of 1. To facilitate this, we modified the pipeline to include events with a depth of 1 read, reasoning that our PacBio reads were of sufficient quality to consider single-occurrence reads.

Including single-read events could introduce noise from sequencing errors, leading to chimeric sequences and inflating detected recombination rates. To mitigate this, we increased pipeline stringency by excluding reads with SNPs differing from the Moneyberg or Moneymaker haplotypes, deletions at any SNP position, or chimeric events with fewer than three consecutive Moneyberg or Moneymaker SNPs. This approach significantly reduced the impact of sequencing errors on the chimeric read rate.

Protoplast screenings enable the examination of hundreds of thousands of genomes but do not allow for our 2D pooling technique since each protoplast genome cannot be sampled twice. This limitation prevented us from distinguishing true recombination events from chimeric molecules caused by PCR template switching. Although using unique molecular identifiers (UMIs) would help differentiate these events, it requires much higher sequencing depth, limiting the number of samples and genomes we could analyze (Karst *et al*. 2021). Instead, we established a noise baseline using control protoplast pools transfected with a nontargeting CRISPR/Cas9 construct. We reasoned that if targeted constructs exhibited a higher crossover frequency than the baseline, putative targeted recombinants would be present. To mitigate early-cycle PCR template switching, which could skew results, we divided each sample into 10 separate PCR reactions, thereby averaging out early-cycle template switches.

**Fig. 5.**
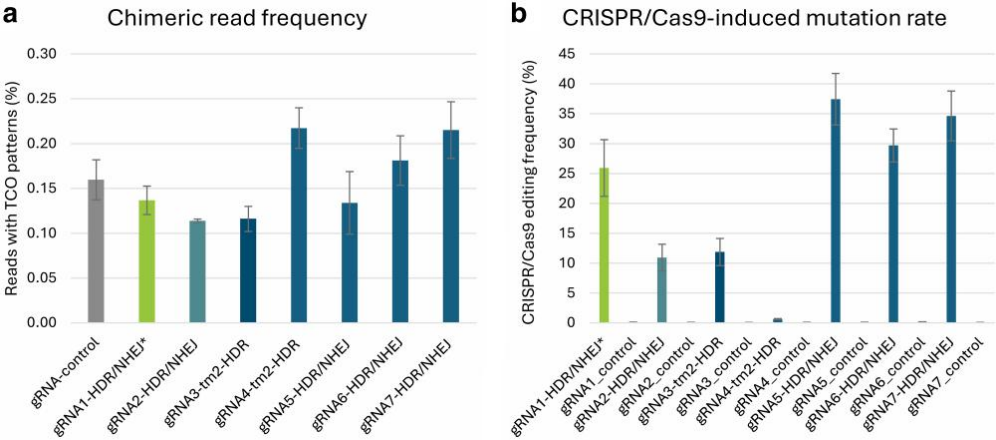
a) Frequency (%) of PacBio sequencing reads containing chimeric amplicons in the ToMV locus for three gRNAs used in the F_1_ seedlings experiment (gRNA1, gRNA2, gRNA3) and four additional gRNAs (gRNA4, gRNA5, gRNA6, gRNA7). b) CRISPR/Cas9-induced mutation rates (%) at the predicted DSB sites for the seven gRNAs, with controls showing mutation frequencies at each target site. The gray bar represents the controls. Error bars indicate the standard error of the mean for the biological replicates (*n* = 4 for each target construct, *n* = 8 for controls).

By analyzing the PacBio sequencing data filtered through our pipeline, we observed a chimeric read occurrence of approximately 0.15% in the control samples (Supplementary Table 11). For true targeted recombination events to become apparent, they should occur well above this threshold. However, we did not find a significant difference in the chimeric read frequency between all gRNAs and the control (Fig. 5a; Tukey HSD; Supplementary Table 12). This indicates that none of the gRNAs targeting the ToMV loci significantly increased the targeted recombination rate. Notably, the prevalence of chimeric molecules in the protoplast pools was substantially higher than in the F_1_ seedling pools, where chimeric molecules were largely absent.

We verified successful transfection and confirmed that the protoplasts contained functional CRISPR/Cas9 constructs by analyzing the PacBio data for mutations at the predicted DSB sites. Indeed, we found that all gRNAs except gRNA4-tm2-HDR efficiently induced DSBs, as indicated by the NHEJ-mediated indels detected at the DSB target sites (Fig. 5b). Control samples did not show these patterns.

## Discussion

### Targeted recombination in somatic cells might occur only in reproducing tissues

Despite our comprehensive experimental design and the extensive analysis of over 9,000 seedlings with potential for CRISPR/Cas9-mediated targeted recombination via HDR or NHEJ, we did not confirm targeted recombination events (Fig. 1, a and b). We used *S. pimpinellifolium* seedlings with distinct SNP patterns as a positive control to verify our detection system. These patterns were detected at the expected sequencing depth in our F_1_ pools. Additionally, the detected SNP patterns accurately corresponded to the original sowing locations of the *S. pimpinellifolium* seeds. The functionality of the CRISPR/Cas9 system was confirmed in F_1_ seedlings by the presence of mutations that resulted from NHEJ-based repair of CRISPR/Cas9-induced DSBs (Fig. 3, a–c; Fig. 4b; Supplementary Fig. 5). F_1_ control crosses did not show indels. A 2D pooling strategy was employed to distinguish genuine targeted recombination events from chimeric sequences caused by PCR artifacts (Fig. 2, d and e). Furthermore, we screened individual F_1_ plants. However, putative targeted recombination events in both the sequencing pools and the individual F_1_ plants were rare and exhibited much lower depth than expected. The presence of chimeric sequences in control plants due to PCR artifacts suggested that true targeted recombination events were not detected. Despite this, the sound experimental setup and controls support that targeted recombinants would likely have been identified if present. Factors that might explain why no targeted recombination events were detected include the developmental stage of the plants analyzed, the number of F_1_ seedlings used, and the specific target region selected for the study.

Previous studies have shown that CRISPR/Cas9-induced somatic targeted recombination between homologous chromosomes is feasible in tomato (Filler Hayut *et al*. 2017; Ben Shlush *et al*. 2021; Samach *et al*. 2023) and *A. thaliana* (Filler-Hayut *et al*. 2021) within recombining chromosomal regions. For example, Filler Hayut *et al*. (2017) and Samach *et al*. (2023) used strategies similar to ours (Fig. 1a) by targeting the wild-type allele in heterozygous F_1_ plants. Still, they differed by focusing on visual markers like fruit color genes, which allowed for the selection of recombination events through phenotypic assessment.

The four abovementioned studies from Avi Levy's lab (Filler Hayut *et al*. 2017; Ben Shlush *et al*. 2021; Samach *et al*. 2023; Kouranov *et al*. 2022) consistently found chimeric patterns for the targeted recombinations. Moreover, the targeted recombinations were observed in most cases at the late stages of plant development. Filler Hayut *et al*. (2017) mentioned that no complete tomato fruits with targeted recombinations were found, but only sectors within fruits, which indicated late recombinations leading to chimerism.

For our 2D pooling strategy of young seedlings, we sampled each seedling twice: a part of one cotyledon for a row pool and a part of the other cotyledon for the column pool. In the case of chimeric targeted recombination, a genuine recombination in one cotyledon that was absent in the other might be labeled as a template switch. Only early targeted recombinations that were present in both cotyledons would be considered for further analysis.

Our data indicate that such early targeted recombinations did not occur, despite the large number of evaluated seedlings. Additionally, it shows that in the 2D pooling approach, chimeric recombinations that occur later during plant development may remain undetected.

To investigate whether targeted recombination occurred in leaves at later developmental stages, we grew 182 F_1_ seedlings from the sowing block showing chimeric sequence patterns and expressing GFP to maturity. GFP expression indicated the presence of CRISPR/Cas9 components. Due to the large number of F1 plants, we could not grow all plants to maturity and did not analyze flower or fruit tissues. ONT sequencing of the 2.5 kbp region spanning the DSB site confirmed CRISPR/Cas9 activity in these plants but not in controls. However, no convincing evidence of targeted recombination was found in seedlings or mature plants. We found recombination patterns at low sequencing depths, which may have been caused by template switching artifacts during PCR.

### The ToMV allele might resist targeted recombination

Another possible explanation for the absence of targeted recombination events in our experiments may be resistance of the ToMV locus to recombination. Traditional breeding has failed to induce recombination in or around the ToMV locus for decades (Pelham 1966; Lin *et al*. 2014). This lack of recombination is supported by the mapping studies of Viquez-Zamora *et al*. (2014) and Demirci *et al*. (2017) where it was indicated that there is a lack of recombination across a substantial portion of chromosome 9. While Cas9 successfully reached target sites and caused mutations (Fig. 3, a–c; Fig. 4b; Supplementary Fig. 5), targeted recombination at the ToMV locus was either too rare to observe or did not occur. Below, we discuss how repair mechanisms might explain resistance of the ToMV locus to recombination.

Using the homologous chromosome as a template for HDR-based repair requires high sequence similarity for accurate alignment and repair, making sister chromatids, which have identical sequences in somatic cells, ideal templates (Takahashi *et al*. 2010). In regions where meiotic recombination is rare due to sequence dissimilarity, HDR using the homologous chromosome is also likely to be rare and inefficient.

The efficiency of HDR in the ToMV locus may be compromised by sequence dissimilarity in flanking regions, which disrupts the process of strand invasion. We targeted the ToMV locus, characterized by overall high sequence similarity within the two haplotypes. However, the surrounding flanking regions exhibit high SNP density and sequence divergence (Supplementary Figs. 8 and 9). Within the ToMV locus, there is a small region with elevated SNP density (Supplementary Fig. 8, position 12,620–12,850 bp). While the overall high sequence similarity within the ToMV locus could promote HDR, the surrounding dissimilarity might hinder the process, particularly during the DNA resection stage facilitated by EXO1 (Zhang *et al*. 2015). EXO1 generates 3′ ssDNA that overhangs through 5′ end resection, potentially extending into regions of low sequence similarity as resectioning can extend for kilobases (Filler Hayut *et al*. 2017; Zakharyevich *et al*. 2010; Ben Shlush *et al*. 2021; Filler-Hayut *et al*. 2021). This extension can disrupt the ability of RAD51 to find homologous sequences, preventing stable strand invasion and D-loop formation, thus hindering HDR and potentially leading to a shift to the NHEJ pathway.

Unlike HDR, NHEJ does not require resectioning or strand invasion, making it potentially less affected by sequence dissimilarity outside the ToMV locus. When both alleles are cut simultaneously in the same cell, NHEJ-mediated targeted recombination can fuse complementary haplotype sequences at the DSB site. However, we did not detect recombination events between SNPs or directly at the DSB site (Table 2). This rarity aligns with findings in maize, where CRISPR/Cas12a-mediated targeted crossover frequencies were low—0.71 and 3.6% for different gRNAs (Kouranov *et al*. 2022). Similarly, in *A. thaliana*, NHEJ-mediated recombination in somatic cells was rare, with only 1 in 453 plants showing this pattern, compared to 17 plants showing HDR-based noncrossover patterns (Filler-Hayut *et al*. 2021). In tomato, targeted crossover events have not been confirmed via molecular markers, but visual fruit markers indicated such events (Filler Hayut *et al*. 2017; Ben Shlush *et al*. 2021). The fewer plants used in these tomato studies compared to the *A. thaliana* study may support the idea that targeted crossovers—whether via HDR or NHEJ—are less common than targeted allele replacements, which require HDR.

### PCR template switching as the cause of chimeric sequences

We detected several chimeric sequences mimicking targeted recombination events in one sowing block (Fig. 4a, Table 2). These chimeric sequences exhibited specific patterns present in both column and row pools from our F_1_ seedling sequencing data but appeared at lower-than-expected sequencing depths. Other blocks with F_1_ plants from the same crosses did not show any chimeric sequences. This discrepancy led us to hypothesize that the chimeric sequences in the F_1_ pools might represent genuine targeted recombination events occurring in a limited number of cells, making them rare. To investigate this, we analyzed ONT sequencing data from mature F_1_ plants, revealing that 97% of the chimeric patterns detected in the PacBio pool/column analysis were also present in individual plant analysis. Additionally, we observed chimeric sequences with their complementary haplotype (Fig. 1, a and b, right window). However, similar chimeric patterns were also observed in control plants. Consequently, we could not firmly conclude that these sequences were true targeted recombination events.

Template switching occurs when the polymerase does not fully amplify the ssDNA during a PCR cycle. The unfinished ssDNA may anneal to the complement haplotype in a subsequent cycle and subsequently be extended by DNA polymerase, like a large primer (Chakravarti and Mailander 2008; Haas *et al*. 2011). This process can form chimeric molecules indistinguishable from true recombination events. To reduce PCR template switching, we minimized the number of PCR cycles during target enrichment (Supplementary Tables 5 and 6). Consequently, chimeric molecules were rare under optimized conditions, aligning with expectations for PCR template switches (Haas *et al*. 2011; Kebschull and Zador 2015). Template switching typically occurs at challenging sites for the polymerase, such as regions that form secondary structures within themselves, with their ssDNA complement strand, or with other ssDNA sequences in the mix (Montgomery *et al*. 2014; Fan *et al*. 2019). Amplifying larger amplicons, as done in this study, increases the chance of polymerase stalling due to more secondary structures. If a sequence pattern between two SNPs causes the polymerase to halt, it could lead to the formation of chimeric molecules during PCR for each haplotype amplicon. This could explain the presence of both the sequence and its complement haplotype in specific sequencing pools. However, this does not account for the majority of chimeric molecules being detected in only one out of fifteen sowing blocks, as polymerase halting would not be specific to one block.

Strikingly, the majority of chimeric molecules detected in only one out of fifteen sowing blocks were associated with the presence of damping-off disease in seedlings within this block. Two possible explanations are proposed for the damping-off disease as having caused the PCR artifacts, leading to chimeric molecules. Firstly, damping-off disease can induce the production of reactive oxygen species (ROS) as a defense mechanism against pathogen invasion. These ROS can cause DNA breaks, resulting in polymerase stalling during PCR amplification (Choudhary *et al*. 2020). In our heterozygous background, this stalling can produce chimeric molecules that mimic recombination events. Secondly, the pathogen may lead to tissue degradation and subsequently degraded DNA within it (Lamichhane *et al*. 2017). Degraded DNA quality can impair polymerase function and increase the likelihood of template switching and chimeric molecule formation. Although DNA quality checks from pooled samples on agarose gel indicated good quality, each pool contained 42 samples, and degraded samples could have been masked by the predominantly high-quality samples.

However, chimeric molecules formed during PCR from damaged genomic DNA would not compromise our ability to detect true targeted recombinants. Genuine targeted recombinants would likely have higher coverage, as indicated by the coverage of the positive *S. pimpinellifolium* controls. Additionally, we would have been able to find the coordinates of the plant via our 2D pooling method and verify recombinant plants through ONT sequencing of the individuals that harbored targeted recombinations.

## Conclusion

We investigated the potential of CRISPR/Cas9 technology to induce somatic targeted recombination within the ToMV resistance locus of *S. lycopersicum*, a region notoriously resistant to natural recombination. We employed two approaches—one focusing on HDR and another combining HDR with NHEJ. Additionally, we developed and implemented a novel 2D pooling method coupled with a bioinformatics pipeline designed to detect chimeric sequences to accurately detect true recombinants. Despite this experimental setup and the extensive analysis of over 9,000 seedlings, 182 mature plants with putative targeted recombination events, and millions of protoplast cells, our results consistently demonstrated that CRISPR/Cas9-induced DSBs were insufficient to disrupt the genetic linkage within this locus at the specific sites tested at a detectable level. This outcome suggests that the ToMV locus may possess inherent resistance to recombination, including somatic targeted recombination, potentially due to its unique genomic context and sequence dissimilarity in flanking regions. This finding underscores the difficulty of inducing somatic recombination in specific genomic regions and underlines the need for further exploration into the conditions under which targeted recombination might be more effectively induced.

## Supplementary Material

jkaf068_Supplementary_Data

## Data Availability

Strains and plasmids are available upon request. The data underlying this article are available in Sequence Read Archive (SRA) at https://www.ncbi.nlm.nih.gov/sra and can be accessed with PRJNA1148103. The software code used for data analysis is available through the Figshare portal at https://doi.org/10.6084/m9.figshare.26582380. This repository includes scripts for data processing and analysis. Supplemental material available at G3 online.
