# Supplementary material for: CRISPR/Cas9-induced breaks are insufficient to break linkage drag surrounding the ToMV locus of Solanum lycopersicum
Source: G3 (Bethesda). 2025 Apr 2;15(6):jkaf068. doi: 10.1093/g3journal/jkaf068 (PMC12159746; doi:10.1093/g3journal/jkaf068)
Supplement: jkaf068_Supplementary_Data [file jkaf068_supplementary_data.docx]

Title:

CRISPR/Cas9-induced breaks are insufficient to break linkage drag surrounding the ToMV locus of *Solanum lycopersicum*

Authors: Jillis Grubben^1,2^, Gerard Bijsterbosch^1^, Burak Aktürk, Richard G.F. Visser^1^, Henk J. Schouten^1,3^

Affiliations: ^1^ Plant Breeding, Wageningen University & Research, Droevendaalsesteeg 1, 6708 PB Wageningen, The Netherlands

^2^ Graduate School Experimental Plant Sciences, Wageningen University & Research

^3^ Corresponding author: [Henk.Schouten@wur.nl](mailto:Henk.Schouten@wur.nl)

High SNP Density Region

Recombination Cold-Spot

0.0

10.0

20.0

30.0

40.0

50.0

60.0

70.0

80.0

ToMV resistance locus

Physical Position on Chromosome 9 (Mbp)

**Supplementary Figure 1**. Schematic representation of chromosome 9 of *S. lycopersicum* cv. Moneyberg. The introgression from *S. peruvianum* resembles the grey high SNP density region. This region harbours also a series of inversions compared to *S. lycopersicum* cv. Moneymaker, and shows a very low recombination frequency. The chromosome is depicted as a blue bar, with physical distance indicated in Mbp. The ToMV resistance locus is marked by a vertical red dashed line. Inversion positions were obtained from work presented by van Rengs et al. (2022). Inversion events were illustrated by orange hourglass-shaped boxes. The grey bar highlights a high SNP density region. This high SNP density region was identified by Schouten et al. (2019). The green bar indicates recombination cold-spots and was bruit from findings provided by Víquez-Zamora et al. (2014).


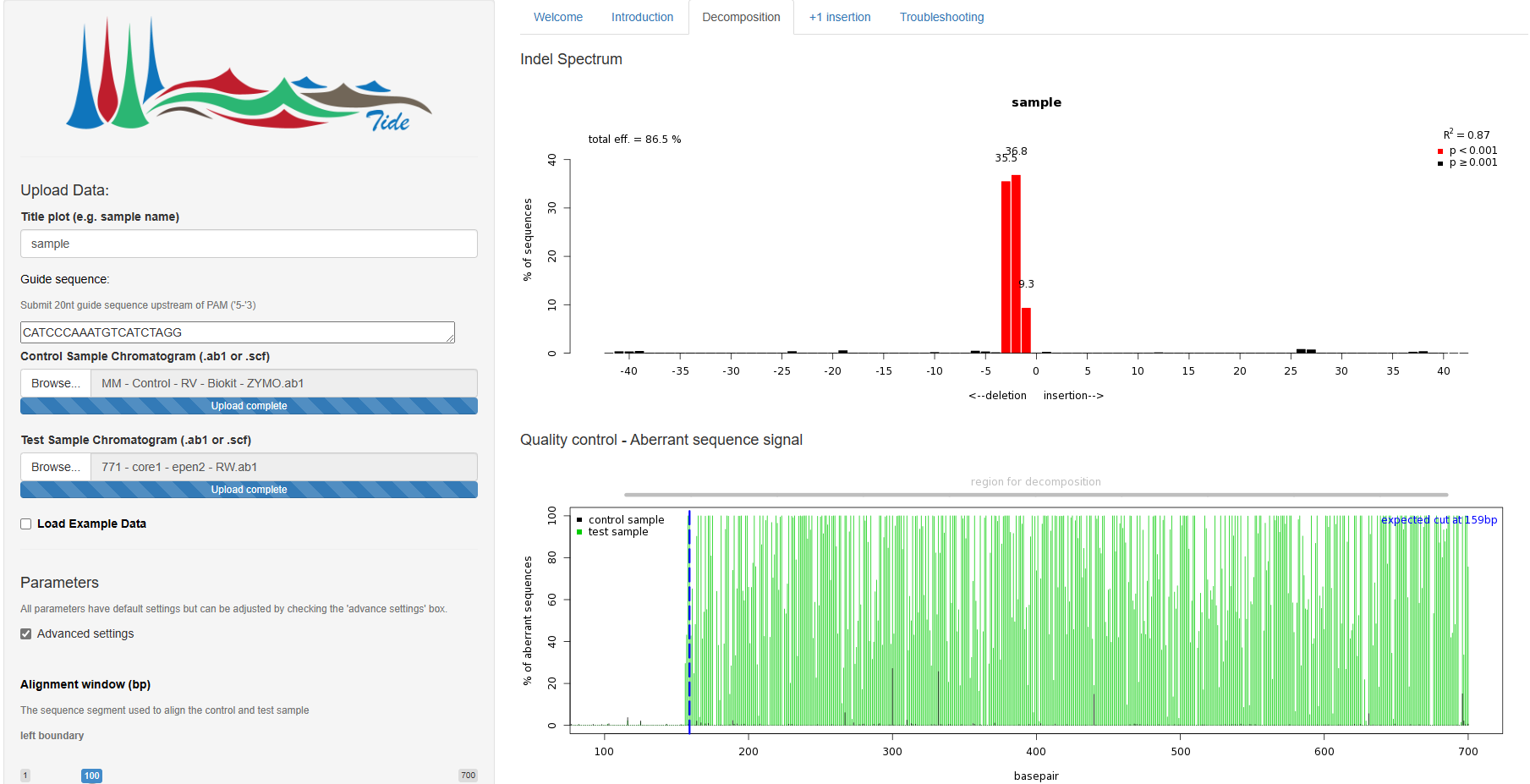

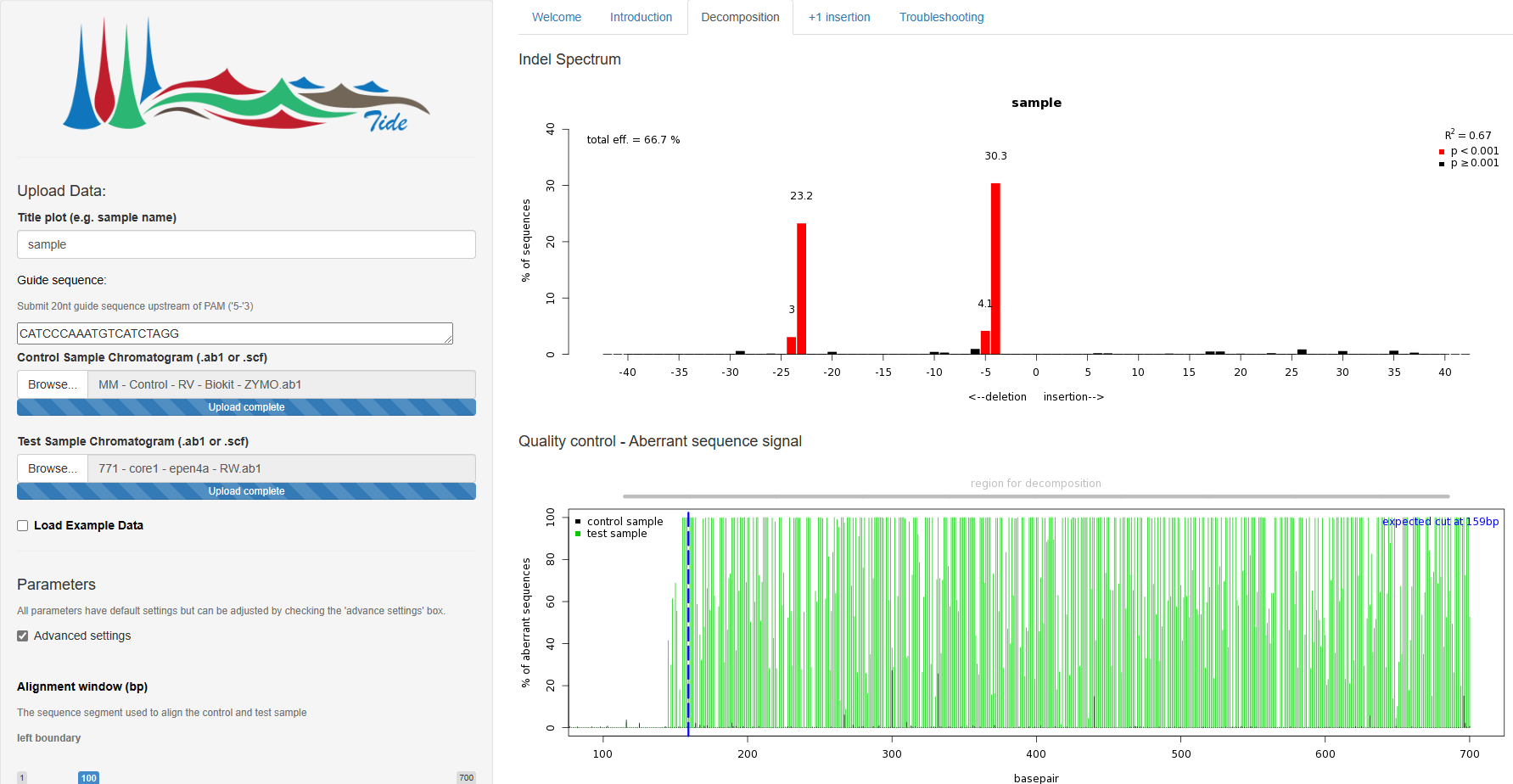

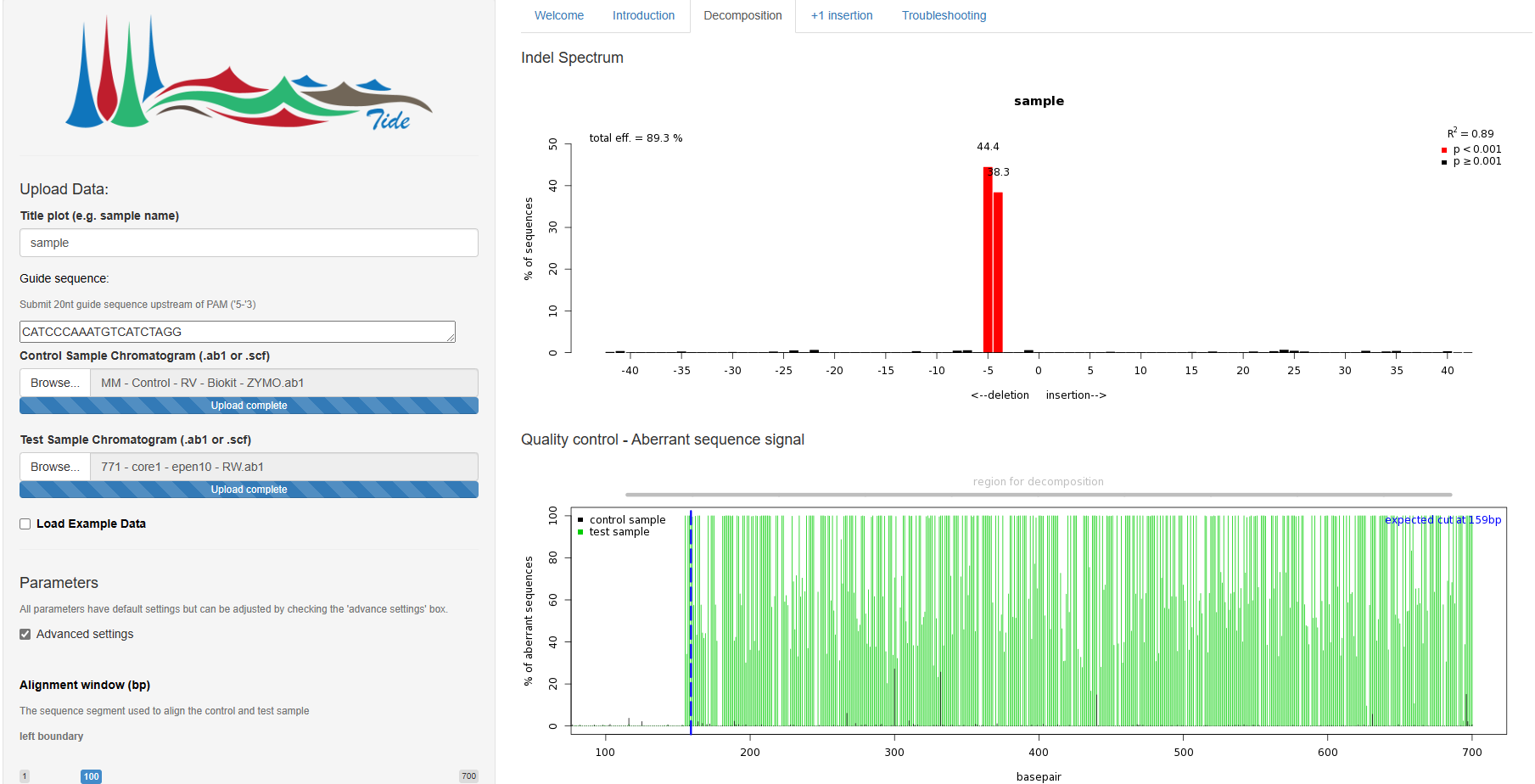

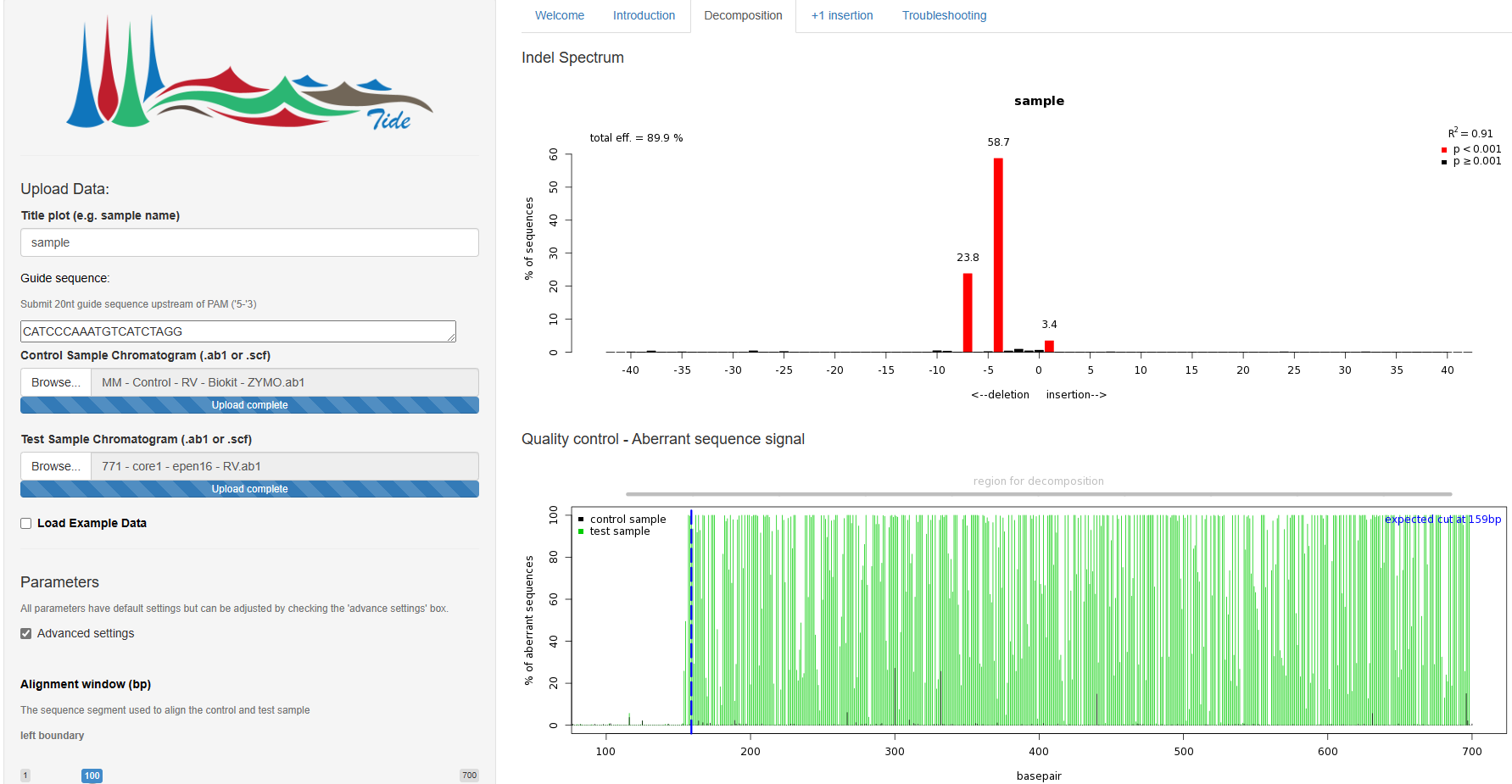

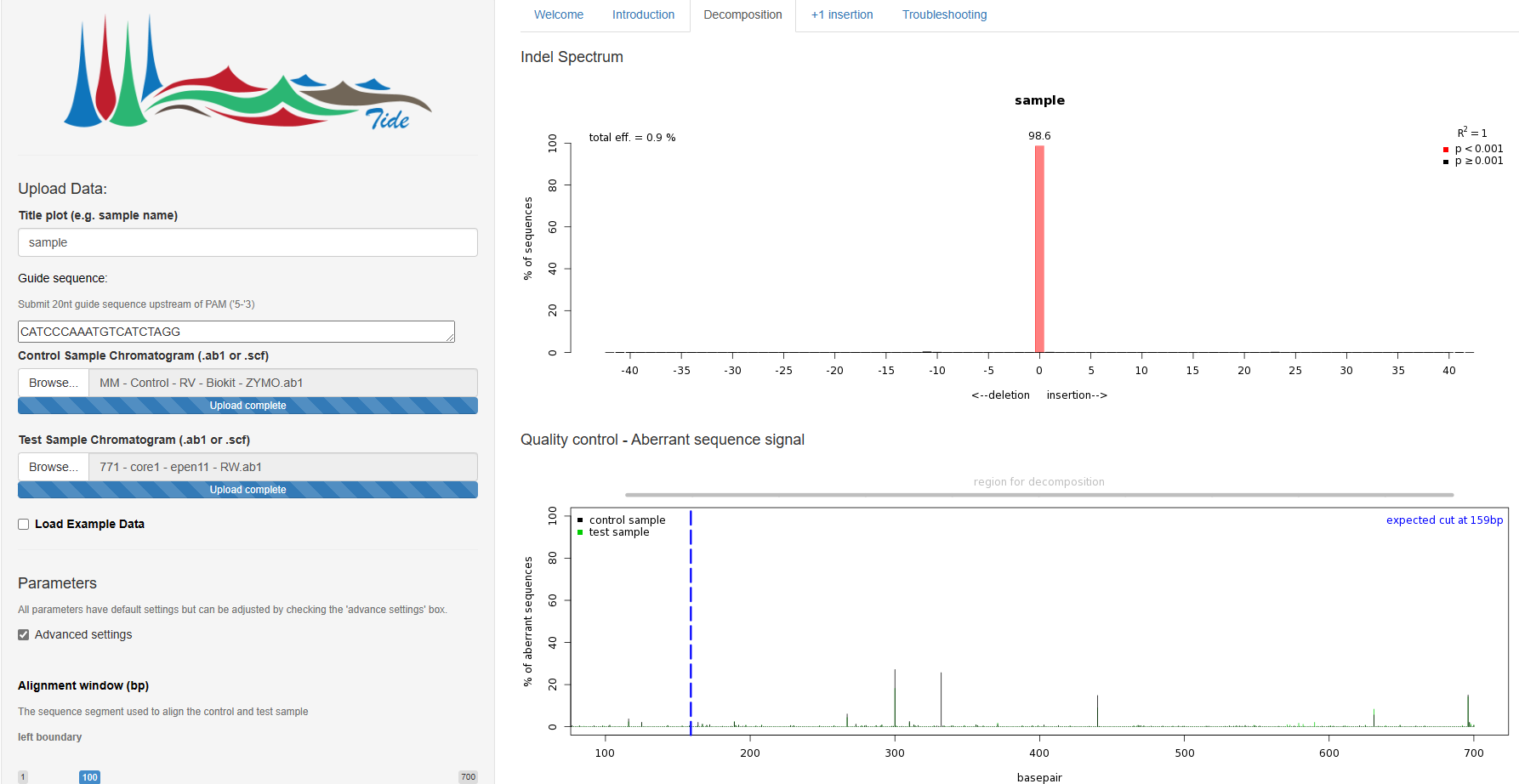


gRNA1-HDR


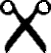


♂ tm-2


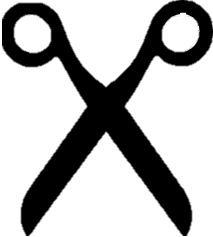


a

b

WT control

T_0_ plant 2

T_0_ plant 4a

T_0_ plant 6

T_0_ plant 10

**Supplementary Figure 2** a. Cartoon of the situation in the T_0_ plants harbouring the CRISPR/Cas9 construct with gRNA1-HDR The gRNA targets both *tm-2* alleles (indicated by blue bars) with the cutting sites marked by scissors icons. Plants with mutations in both alleles (indicated by red boxes in the *tm-2* alleles) were selected. b. TiDE Sanger Sequencing mutation analysis at the gRNA1-HDR target site for a WT control plant and four individual T_0_ plants selected for this study. The y-axis represents the percentage of sequences with a mutation, and the x-axis shows deletions and/or insertions relative to the predicted DSB location. Red bars indicate statistically significant differences from the WT sequence (p < 0.001), while black bars represent insignificant differences. The light red bar in the WT control plant indicates that 100% of the reads are WT sequence.


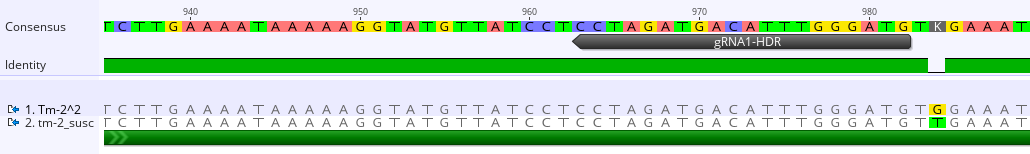


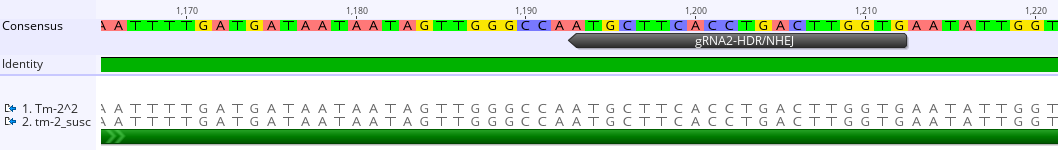


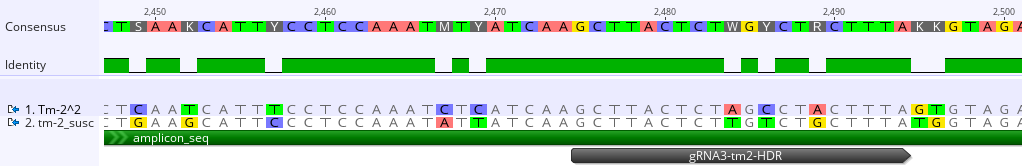


**Supplementary Figure 3**. Alignment between *Tm-2^2^* and *tm-2* at the target sites of gRNA1-HDR, gRNA2-HDR/NHEJ, and gRNA3-tm2-HDR.

*Alignment of Tm-2^2^ and tm-2.*

| Consensus CATAGAAAAAAGAAATTTCTCCTTTTTCATTAATGTGCAGCTGCCCCACGTTGTCACTCC 60 |
| --- |
| Tm-2^2 ............................................................ 60 |
| tm-2_susc -........................................................... 59 |
|  |
| Consensus CCTTCTTCTTTATMATTTCCTTCTTGACNATTATTAGGAGACTTGGCCGWGGACTCCATC 120 |
| Tm-2^2 .............C..............-....................T.......... 119 |
| tm-2_susc .............A..............A....................A.......... 119 |
|  |
| Consensus TACCACTAAAAAGCTAAAGCCATCAGTATACTCATTTTTTGGTAGCTACTGAAAAAGAGA 180 |
| Tm-2^2 ............................................................ 179 |
| tm-2_susc ............................................................ 179 |
|  |
| Consensus GAAAAAAAAATGGCTGAAATTCTTCTTACATCAGTAATCAATAAATCTGTAGAAATAGCT 240 |
| Tm-2^2 ............................................................ 239 |
| tm-2_susc ............................................................ 239 |
|  |
| Consensus GGAAATTTACTGATTCAAGAAGGAAAGCGTTTATATTGGTTGAAAGAGGATATCGATTGG 300 |
| Tm-2^2 ............................................................ 299 |
| tm-2_susc ............................................................ 299 |
|  |
| Consensus CTCCAGAGAGAAATGAGACACATTCGATCTTATGTTGACAACGCAAAGGCCAAGGAAGCT 360 |
| Tm-2^2 ............................................................ 359 |
| tm-2_susc ............................................................ 359 |
|  |
| Consensus GGAGGTGATTCAAGGGTCAAAAACTTATTGAAAGATATTCAAGAATTGGCAGGTGATGTG 420 |
| Tm-2^2 ............................................................ 419 |
| tm-2_susc ............................................................ 419 |
|  |
| Consensus GAGGATCTCTTAGATGACTTCCTTCCAAAAATTCAACRATCCAATAAGTTCAATTATTGC 480 |
| Tm-2^2 .....................................A...................... 479 |
| tm-2_susc .....................................G...................... 479 |
|  |
| Consensus CTTAAGASGAGTTCTTTTGCRGATGAGTTTGCTATGGAGATTGAGAAGATAAAGAGAAGG 540 |
| Tm-2^2 .......G............A....................................... 539 |
| tm-2_susc .......C............G....................................... 539 |
|  |
| Consensus GTTGTTGACATTGACCGAATAAGGAAAACTTACAACATCATAGATACAGATAACAATAAT 600 |
| Tm-2^2 ............................................................ 599 |
| tm-2_susc ............................................................ 599 |
|  |
| Consensus GATGATTGTGTTYTGCTGGATCGGAGAAGATTATTCCTACATGCTGATGAAACAGAGATC 660 |
| Tm-2^2 ............C............................................... 659 |
| tm-2_susc ............T............................................... 659 |
|  |
| Consensus ATCGGTTTGGATGATGACTTCAATATGCTACAAGCCAAATTACTYAATCAAGATTTGCAT 720 |
| Tm-2^2 ............................................T............... 719 |
| tm-2_susc ............................................C............... 719 |
|  |
| Consensus TATGGAGTTGTTTCCATAGTTGGCATGCCCGGTCTGGGGAAAACAACTCTTGCCAAGAAA 780 |
| Tm-2^2 ............................................................ 779 |
| tm-2_susc ............................................................ 779 |
|  |
| Consensus CTTTATAGGCTCATTCGTGATCAATTTGAGTGTTCTGGACTGGTCTACGTTTCACAACAG 840 |
| Tm-2^2 ............................................................ 839 |
| tm-2_susc ............................................................ 839 |
|  |
| Consensus CCAAGAGCGRGTGAAATCTTACTTGACATTGCCAAACAAATTGGACTGACGGAACAGAAA 900 |
| Tm-2^2 .........A.................................................. 899 |
| tm-2_susc .........G.................................................. 899 |
|  |
| Consensus ATKAAGGAAAATTTGGAGGACAACCTGCGATCACTCTTGAAAATAAAAAGGTATGTTATC 960 |
| Tm-2^2 ..G......................................................... 959 |
| tm-2_susc ..T......................................................... 959 |
|  |
| Consensus CTCCTAGATGACATTTGGGATGTKGAAATTTGGGATGATCTGAAACTTGTCCTTCCTGAA 1020 |
| Tm-2^2 .......................G.................................... 1019 |
| tm-2_susc .......................T.................................... 1019 |
|  |
| Consensus TGTGAYTCAAAAGTCGGCAGTAGAATGATAATCACGTCTCGAAATAGTAATGTAGGCAGA 1080 |
| Tm-2^2 .....T...................................................... 1079 |
| tm-2_susc .....C...................................................... 1079 |
|  |
| Consensus TACATAGGAGGGGAATCCTCCCTCCATGCATTGCAACCCCTAGAATCCGAGAAAAGCTTT 1140 |
| Tm-2^2 ............................................................ 1139 |
| tm-2_susc ............................................................ 1139 |
|  |
| Consensus GAACTCTTTACCAAGAAAATCTTTAATTTTGATGATAATAATAGTTGGGCCAATGCTTCA 1200 |
| Tm-2^2 ............................................................ 1199 |
| tm-2_susc ............................................................ 1199 |
|  |
| Consensus CCTGACTTGGTGAATATTGGTAGAAATATAGYTGGGAGATGTGGAGGTATACCGCTAGCC 1260 |
| Tm-2^2 ...............................T............................ 1259 |
| tm-2_susc ...............................C............................ 1259 |
|  |
| Consensus ATAGTGGTGACTGCAGGCATGTTAAGGGCAAGAGAAAGAACAGAACATGCGTGGAACAGA 1320 |
| Tm-2^2 ............................................................ 1319 |
| tm-2_susc ............................................................ 1319 |
|  |
| Consensus GTACTTGAGAGTATGGGCCATAAAGTTCAAGATGGATGTGCTAAGGTATTGGCTCTCAGT 1380 |
| Tm-2^2 ............................................................ 1379 |
| tm-2_susc ............................................................ 1379 |
|  |
| Consensus TACAATGATTTACCKATTGCCTCAAGGCCATGTTTCTTGTACTTTRGCCTTTACCCCGAG 1440 |
| Tm-2^2 ..............T..............................G.............. 1439 |
| tm-2_susc ..............G..............................A.............. 1439 |
|  |
| Consensus GACCATGAAATTCGTGCTTTTGATTTGATAAATATGTGGATTGCTGAGAAGTTTATWGTA 1500 |
| Tm-2^2 ........................................................A... 1499 |
| tm-2_susc ........................................................T... 1499 |
|  |
| Consensus GTAAATAGTGGTAATAGGCGAGAGGCTGAGGATTTGGCGGAGGACGTCCTAAATGATTTG 1560 |
| Tm-2^2 ............................................................ 1559 |
| tm-2_susc ............................................................ 1559 |
|  |
| Consensus GTTTCTAGAAACTTGATTCAACTTGCCAAAAGGACATATAATGGAAGAATTTCAAGTTGT 1620 |
| Tm-2^2 ............................................................ 1619 |
| tm-2_susc ............................................................ 1619 |
|  |
| Consensus CGCATACATGACTTGTTACATAGTTTGTGTGTGGACTTGGCTAAGGAAAGTAACTTCTTT 1680 |
| Tm-2^2 ............................................................ 1679 |
| tm-2_susc ............................................................ 1679 |
|  |
| Consensus CACACCGCGCATGATGYATTTGGTGATCCCGGCAATGTYGCTAGGCTYCGAAGGATTACA 1740 |
| Tm-2^2 ................C.....................T........C............ 1739 |
| tm-2_susc ................T.....................C........T............ 1739 |
|  |
| Consensus TTCTACTCTGACAATGTCATGATTGAGTTCTTCSGTTCWAATCCTAAGCTTGAGAAGCTT 1800 |
| Tm-2^2 .................................C....A..................... 1799 |
| tm-2_susc .................................G....T..................... 1799 |
|  |
| Consensus CGTGTACTTTTCTGTTTCRCAAAAGACCCTTCCATATTTTCTCATATGGCTTRTTTTGAC 1860 |
| Tm-2^2 ..................G.................................A....... 1859 |
| tm-2_susc ..................A.................................G....... 1859 |
|  |
| Consensus TTCAAATTGTTGCACACATTGGTTGTAGTCATGTCTCAAAGTTTTCAAGCATATGTCACT 1920 |
| Tm-2^2 ............................................................ 1919 |
| tm-2_susc ............................................................ 1919 |
|  |
| Consensus ATCCCAAGCAAATTTGGGAACATGACTTGCTTACGCTATCTGARATTGGAGGGGAATATT 1980 |
| Tm-2^2 ...........................................G................ 1979 |
| tm-2_susc ...........................................A................ 1979 |
|  |
| Consensus TGTGGAAAACTGCCAAATAGTATTGTCAAGCTCACACGTCTAGAGACCATAGACATTGAT 2040 |
| Tm-2^2 ............................................................ 2039 |
| tm-2_susc ............................................................ 2039 |
|  |
| Consensus CGACGTAGCCTCATTCAACYTCCTTCTGGTGTTTGGGAGTCTAAACATTTGAGACATCTT 2100 |
| Tm-2^2 ...................C........................................ 2099 |
| tm-2_susc ...................T........................................ 2099 |
|  |
| Consensus TGTTATAGAGATTATGGACAAGCATGTAACAGTTGCTTTTCTATAAGCTCATTTTACCCA 2160 |
| Tm-2^2 ............................................................ 2159 |
| tm-2_susc ............................................................ 2159 |
|  |
| Consensus AAYATTTACTCATTGCATCCTAACAATCTACAAACCTTGATGTGGATACCTGATAAATTT 2220 |
| Tm-2^2 ..T......................................................... 2219 |
| tm-2_susc ..C......................................................... 2219 |
|  |
| Consensus TTTGAACCGAGGTTGTTGCACCGATTGATCAATTTAAGAAAACTGGGTATACTGGGAGTG 2280 |
| Tm-2^2 ............................................................ 2279 |
| tm-2_susc ............................................................ 2279 |
|  |
| Consensus TCCAATTCWACCGTTAAGATRTTATCAAYATKTMGCCCTGTGCYWAAGGCGCTRRAGGTT 2340 |
| Tm-2^2 ........T...........G.......T..T.A.........TT........GG..... 2339 |
| tm-2_susc ........A...........A.......C..G.C.........CA........AA..... 2339 |
|  |
| Consensus CTGAAGCTCAGKTTTTYCAGTGAYCCGAGTGARCAAATAAASTTGTCATCSTATCCAMAW 2400 |
| Tm-2^2 ...........T....C......C........A........G........G......C.T 2399 |
| tm-2_susc ...........G....T......T........G........C........C......A.A 2399 |
|  |
| Consensus ATTGYTAAGTTGCATTTGAATGTTRACAGAACAATRGCCTTGAACTCTSAAKCATTYCCT 2460 |
| Tm-2^2 ....C...................A..........G............C..T....T... 2459 |
| tm-2_susc ....T...................G..........A............G..G....C... 2459 |
|  |
| Consensus CCAAATMTYATCAAGCTTACTCTWGYCTRCTTTAKKGTAGACMGTTRTMTACTGGCAGTR 2520 |
| Tm-2^2 ......C.C..............A.C..A.....GT......C...A.A..........A 2519 |
| tm-2_susc ......A.T..............T.T..G.....TG......A...G.C..........G 2519 |
|  |
| Consensus CTTAAGACATTWCCCAAATTAAGAAAACTTAAAATGKTCATCTGCAAGTATAATGAAGAA 2580 |
| Tm-2^2 ...........T........................T....................... 2579 |
| tm-2_susc ...........A........................G....................... 2579 |
|  |
| Consensus AAGATGGMTCTCTCGGGCGAGGCAAATGGTTATAGCTTTCCGCAACTTGAAGTTTTGCAT 2640 |
| Tm-2^2 .......A.................................................... 2639 |
| tm-2_susc .......C.................................................... 2639 |
|  |
| Consensus ATTCATAGCCCGAATGGGTTGTCTGAAGTAACRTGCACGGATGATGTCAGTATGCCCAAA 2700 |
| Tm-2^2 ................................G........................... 2699 |
| tm-2_susc ................................A........................... 2699 |
|  |
| Consensus TTGAAAAAGCTGTTACTTACAGGATTCCATTGCSGAATCAGTTTATCGGAACGGCTTAAA 2760 |
| Tm-2^2 .................................C.......................... 2759 |
| tm-2_susc .................................G.......................... 2759 |
|  |
| Consensus AAGCTGAGTAAATGAACATCTCAACAGGTCAGTTTGCTAGTATAACTATTTACGTACAGG 2820 |
| Tm-2^2 ............................................................ 2819 |
| tm-2_susc ............................................................ 2819 |
|  |
| Consensus GGTGCATCACAGCAACGTGAGTGTAGTAGTGTTGTTTCTTGTTGTGGTTGTTTGTTGGTT 2880 |
| Tm-2^2 ............................................................ 2879 |
| tm-2_susc .----------------------------------------------------------- 2879 |
|  |
| **Supplementary Figure 4.** Alignment between *Tm-2^2^* and *tm-2*. |
|  |
|  |
|  |
|  |
|  |
|  |


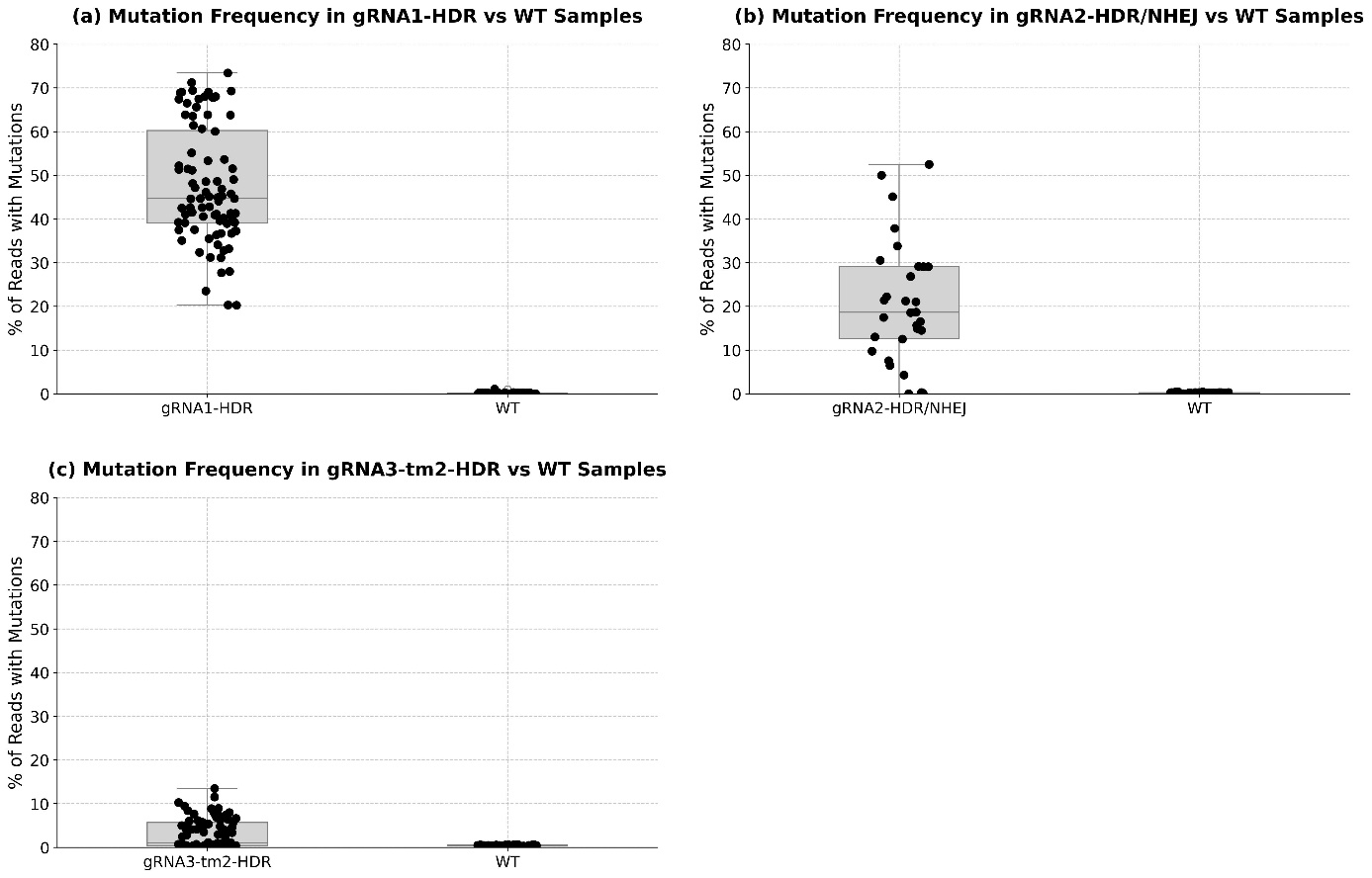


**Supplementary Figure 5.** Mutation frequency in F1 seedling pools associated with three different CRISPR/Cas9 targets: (a) gRNA1-HDR, (b) gRNA2-HDR/NHEJ, and (c) gRNA3-tm2-HDR. The Y-axis represents the percentage of sequencing reads that contain CRISPR/Cas9-induced mutations. Each dot corresponds to an individual F1 plant pool. Only pools containing samples from a single gRNA were included in the analysis; mixed-genotype pools were excluded to prevent skewed results.


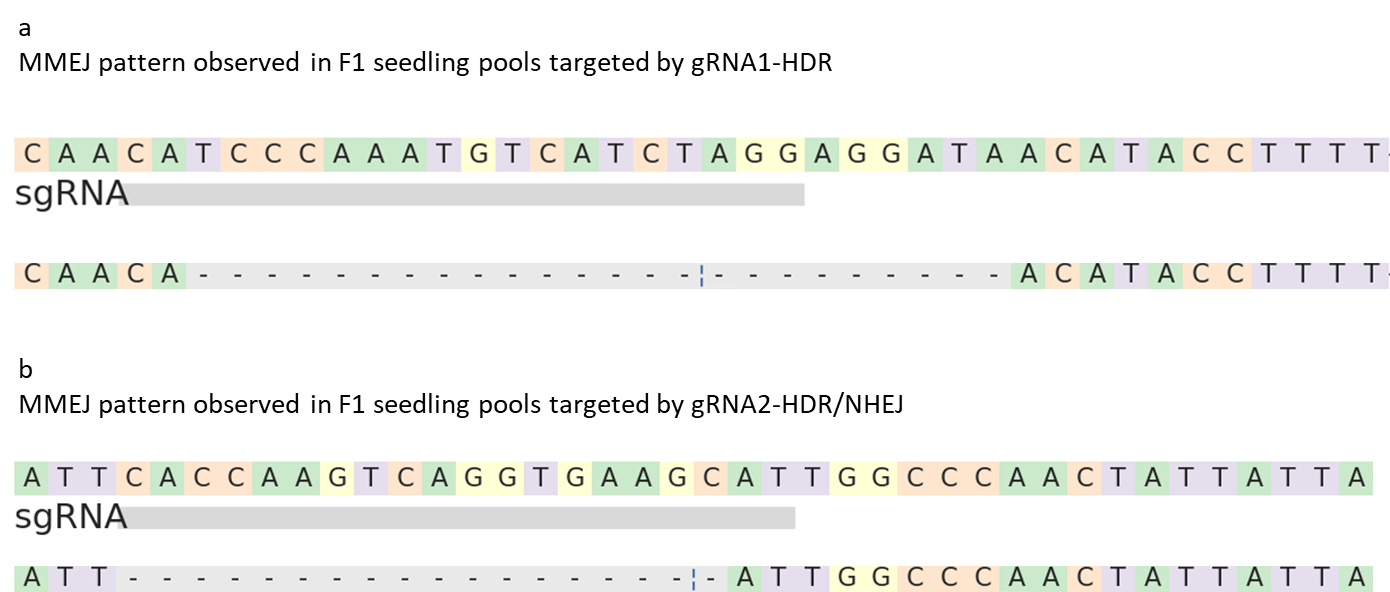


**Supplementary Figure 6.** Combined and cropped CRISPResso2 output illustrating mutation sequences resulting from MMEJ-based repair at the gRNA1-HDR (a) and gRNA2-HDR/NHEJ (b) target sites. Non-MMEJ repair events were excluded to focus exclusively on MMEJ-mediated modifications.


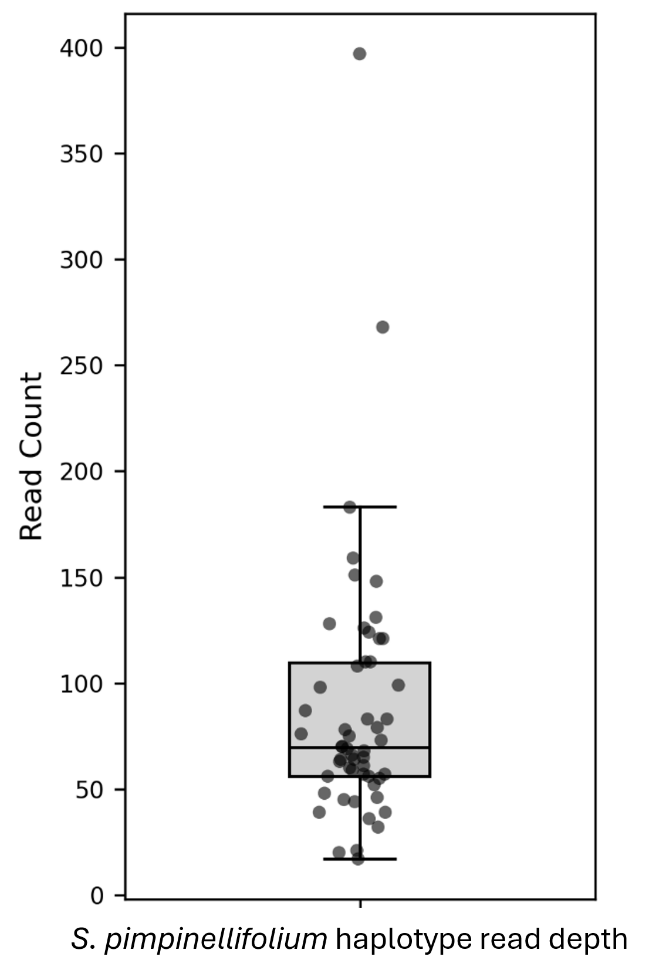


**Supplementary Figure 7.** Boxplot illustrating the distribution of read depths for pools with the *S. pimpinellifolium* haplotype string pattern outputted by the targeted recombination pipeline.


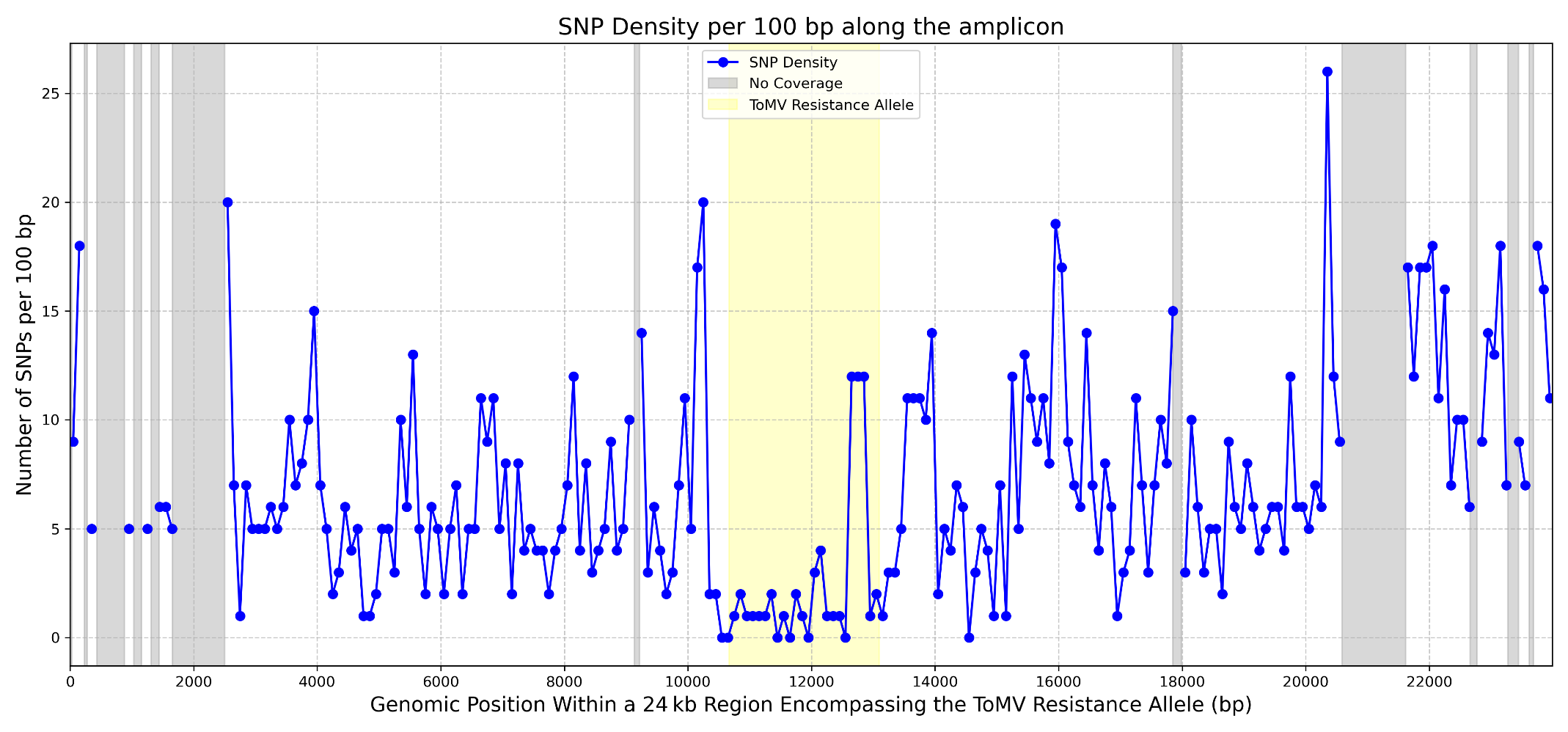


**Supplementary Figure 8.** SNP density across a 24 kb genomic region containing the ToMV resistance locus in *S.* lycopersicum cv. Moneymaker and Moneyberg. The plot shows the SNP density per 100 bp, with the ToMV resistance allele highlighted in yellow. The grey shaded regions indicate intervals where alignment coverage was absent due to high sequence divergence, resulting in gaps in SNP density data.


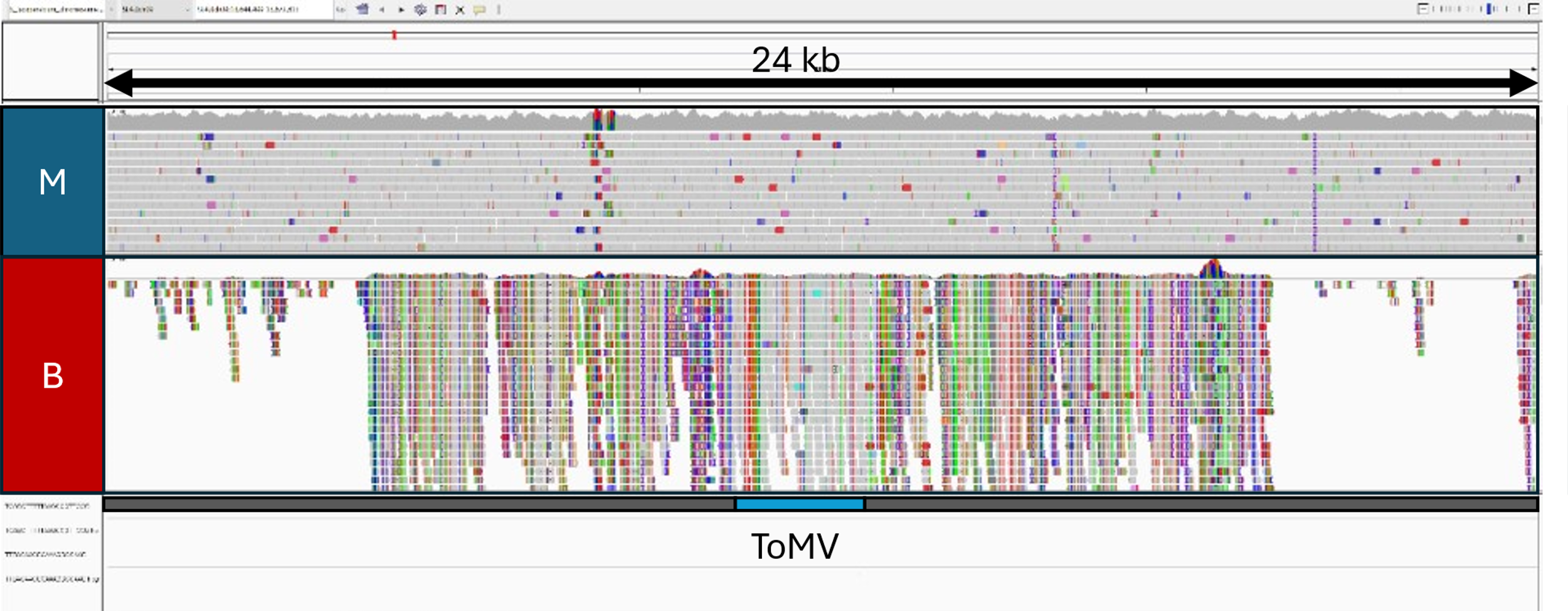


**Supplementary Figure 9.** Cartoon showing an IGV cut-out of the alignment of *S. lycopersicum* cv. Moneymaker (Blue square indicated with “M”) and *S. lycopersicum* cv. Moneyberg (Red square indicated with “B”) to the ITAG 4.0 reference genome around the ToMV locus. The ToMV locus is highlighted by a light blue bar, while the flanking regions are represented by gray bars. The alignment shows a region of 24 kb.

**Supplementary Table 1.** Overview of gRNA names, allele targets, target site positions, and sequences on chromosome 9.

| **gRNA name** | **Target allele** | **Location on SL4.0ch9** | **Sequence (5’- to to 3’ - end)** |
| --- | --- | --- | --- |
| gRNA1-HDR | *tm-2 and Tm-2^2^* | 13.658.688 | CATCCCAAATGTCATCTAGG |
| gRNA2-HDR/NHEJ | *tm-2 and Tm-2^2^* | 13.658.458 | CACCAAGTCAGGTGAAGCAT |
| gRNA3-tm2-HDR | *tm-2* | 13.657.331 | GCTTACTCTTGTCTGCTTTA |
| gRNA4-tm2-HDR | *tm-2* | 13.657.335 | CCCTGTGCCAAAGGCGCTAA |
| gRNA5-HDR/NHEJ | *tm-2 and Tm-2^2^* | 13.658.130 | AGAGGCTGAGGATTTGGCGG |
| gRNA6-HDR/NHEJ | *tm-2 and Tm-2^2^* | 13.657.621 | GCTACGTCGATCAATGTCTA |
| gRNA7-HDR/NHEJ | *tm-2 and Tm-2^2^* | 13.657.421 | ATCAATCGGTGCAACAACCT |
| Control gRNA | none | none | TCCTACTGATCATACCTCCC |

**Supplementary Table 2.** Construct names and sequences used to generate CRISPR/Cas9 constructs used for targeted recombination induction.

| **Plasmid content** | **Sequence (5’-> 3’)** |
| --- | --- |
| NosP::NPTII | ATGGTTGAACAAGATGGATTGCACGCAGGTTCTCCGGCCGCTTGGGTGGAGAGGCTATTCGGCTATGACTGGGCACAACAGACAATCGGCTGCTCTGATGCCGCCGTGTTCCGGCTGTCAGCGCAGGGGCGCCCGGTTCTTTTTGTCAAGACCGACCTGTCCGGTGCCCTGAATGAACTGCAGGACGAGGCAGCGCGGCTATCGTGGCTGGCCACGACGGGCGTTCCTTGCGCAGCTGTGCTCGACGTTGTCACTGAAGCGGGAAGGGACTGGCTGCTATTGGGCGAAGTGCCGGGGCAGGATCTCCTGTCATCTCACCTTGCTCCTGCCGAGAAAGTATCCATCATGGCTGATGCAATGCGGCGGCTGCATACGCTTGATCCGGCTACCTGCCCATTCGACCACCAAGCGAAACATCGCATCGAGCGAGCACGTACTCGGATGGAAGCCGGTCTTGTCGATCAGGATGATCTGGACGAAGAGCATCAGGGGCTCGCGCCAGCCGAACTGTTCGCCAGGCTCAAGGCGCGCATGCCCGACGGCGAGGATCTCGTCGTGACTCATGGCGATGCCTGCTTGCCGAATATCATGGTGGAAAATGGCCGCTTTTCTGGATTCATCGACTGTGGCCGGCTGGGTGTGGCGGACCGCTATCAGGACATAGCGTTGGCTACCCGTGATATTGCTGAAGAGCTTGGCGGCGAATGGGCTGACCGCTTCCTCGTGCTTTACGGTATCGCCGCTCCCGATTCGCAGCGCATCGCCTTCTATCGCCTTCTTGACGAGTTCTTCTGA |
| pCsVMV::turboGFP | ATGAGAGGATCTGGATCTGAGTCTGATGAGTCTGGACTTCCTGCTATGGAAATCGAGTGTAGAATCACTGGAACCCTTAACGGTGTTGAGTTCGAGCTTGTTGGAGGTGGTGAGGGAACTCCTGAGCAGGGAAGAATGACTAACAAGATGAAGTCTACCAAGGGTGCTCTTACCTTCTCTCCATACCTTCTTTCTCACGTTATGGGATACGGATTCTACCACTTCGGAACTTACCCATCTGGATACGAGAACCCTTTCCTTCATGCTATCAACAACGGTGGATACACCAACACTAGGATCGAGAAGTACGAGGATGGTGGTGTTCTTCACGTTAGCTTCTCTTACAGATACGAGGCTGGAAGAGTGATCGGAGATTTCAAGGTTATGGGAACTGGATTCCCTGAGGATTCTGTTATCTTCACCGACAAGATCATCAGGTCTAACGCTACTGTTGAGCATCTTCATCCTATGGGAGATAACGATCTCGATGGATCTTTCACCAGAACCTTCTCACTTAGAGATGGTGGTTACTACTCTTCTGTGGTGGATTCTCACATGCACTTCAAGTCTGCTATCCACCCTTCTATCCTTCAAAACGGTGGACCTATGTTCGCTTTCAGAAGAGTTGAGGAAGATCACTCTAACACCGAGCTTGGAATCGTTGAGTACCAACATGCTTTCAAGACCCCTGATGCTGATGCTGGTGAGGAATAG |
| pUBI::Cas9 | ATGTATATATGTAGATCTGGACTTTTTGGAGTTGTTGACTTGATTGTATTTGTGTGTGTATATGTGTGTTCTGATCTTGATATGTTATGTATGTGCAGCTACTGTATTTTTACAACAATTACCAACAACAACAAACAACAAACAACATTACAATTACTATTTACAATTACAATGGATAAGAAGTACTCTATCGGACTCGATATCGGAACTAACTCTGTGGGATGGGCTGTGATCACCGATGAGTACAAGGTGCCATCTAAGAAGTTCAAGGTTCTCGGAAACACCGATAGGCACTCTATCAAGAAAAACCTTATCGGTGCTCTCCTCTTCGATTCTGGTGAAACTGCTGAGGCTACCAGACTCAAGAGAACCGCTAGAAGAAGGTACACCAGAAGAAAGAACAGGATCTGCTACCTCCAAGAGATCTTCTCTAACGAGATGGCTAAAGTGGATGATTCATTCTTCCACAGGCTCGAAGAGTCATTCCTCGTGGAAGAAGATAAGAAGCACGAGAGGCACCCTATCTTCGGAAACATCGTTGATGAGGTGGCATACCACGAGAAGTACCCTACTATCTACCACCTCAGAAAGAAGCTCGTTGATTCTACTGATAAGGCTGATCTCAGGCTCATCTACCTCGCTCTCGCTCACATGATCAAGTTCAGAGGACACTTCCTCATCGAGGGTGATCTCAACCCTGATAACTCTGATGTGGATAAGTTGTTCATCCAGCTCGTGCAGACCTACAACCAGCTTTTCGAAGAGAACCCTATCAACGCTTCAGGTGTGGATGCTAAGGCTATCCTCTCTGCTAGGCTCTCTAAGTCAAGAAGGCTTGAGAACCTCATTGCTCAGCTCCCTGGTGAGAAGAAGAACGGACTTTTCGGAAACTTGATCGCTCTCTCTCTCGGACTCACCCCTAACTTCAAGTCTAACTTCGATCTCGCTGAGGATGCAAAGCTCCAGCTCTCAAAGGATACCTACGATGATGATCTCGATAACCTCCTCGCTCAGATCGGAGATCAGTACGCTGATTTGTTCCTCGCTGCTAAGAACCTCTCTGATGCTATCCTCCTCAGTGATATCCTCAGAGTGAACACCGAGATCACCAAGGCTCCACTCTCAGCTTCTATGATCAAGAGATACGATGAGCACCACCAGGATCTCACACTTCTCAAGGCTCTTGTTAGACAGCAGCTCCCAGAGAAGTACAAAGAGATTTTCTTCGATCAGTCTAAGAACGGATACGCTGGTTACATCGATGGTGGTGCATCTCAAGAAGAGTTCTACAAGTTCATCAAGCCTATCCTCGAGAAGATGGATGGAACCGAGGAACTCCTCGTGAAGCTCAATAGAGAGGATCTTCTCAGAAAGCAGAGGACCTTCGATAACGGATCTATCCCTCATCAGATCCACCTCGGAGAGTTGCACGCTATCCTTAGAAGGCAAGAGGATTTCTACCCATTCCTCAAGGATAACAGGGAAAAGATTGAGAAGATTCTCACCTTCAGAATCCCTTACTACGTGGGACCTCTCGCTAGAGGAAACTCAAGATTCGCTTGGATGACCAGAAAGTCTGAGGAAACCATCACCCCTTGGAACTTCGAAGAGGTGGTGGATAAGGGTGCTAGTGCTCAGTCTTTCATCGAGAGGATGACCAACTTCGATAAGAACCTTCCAAACGAGAAGGTGCTCCCTAAGCACTCTTTGCTCTACGAGTACTTCACCGTGTACAACGAGTTGACCAAGGTTAAGTACGTGACCGAGGGAATGAGGAAGCCTGCTTTTTTGTCAGGTGAGCAAAAGAAGGCTATCGTTGATCTCTTGTTCAAGACCAACAGAAAGGTGACCGTGAAGCAGCTCAAAGAGGATTACTTCAAGAAAATCGAGTGCTTCGATTCAGTTGAGATTTCTGGTGTTGAGGATAGGTTCAACGCATCTCTCGGAACCTACCACGATCTCCTCAAGATCATTAAGGATAAGGATTTCTTGGATAACGAGGAAAACGAGGATATCTTGGAGGATATCGTTCTTACCCTCACCCTCTTTGAAGATAGAGAGATGATTGAAGAAAGGCTCAAGACCTACGCTCATCTCTTCGATGATAAGGTGATGAAGCAGTTGAAGAGAAGAAGATACACTGGTTGGGGAAGGCTCTCAAGAAAGCTCATTAACGGAATCAGGGATAAGCAGTCTGGAAAGACAATCCTTGATTTCCTCAAGTCTGATGGATTCGCTAACAGAAACTTCATGCAGCTCATCCACGATGATTCTCTCACCTTTAAAGAGGATATCCAGAAGGCTCAGGTTTCAGGACAGGGTGATAGTCTCCATGAGCATATCGCTAACCTCGCTGGATCTCCTGCAATCAAGAAGGGAATCCTCCAGACTGTGAAGGTTGTGGATGAGTTGGTGAAGGTGATGGGAAGGCATAAGCCTGAGAACATCGTGATCGAAATGGCTAGAGAGAACCAGACCACTCAGAAGGGACAGAAGAACTCTAGGGAAAGGATGAAGAGGATCGAGGAAGGTATCAAAGAGCTTGGATCTCAGATCCTCAAAGAGCACCCTGTTGAGAACACTCAGCTCCAGAATGAGAAGCTCTACCTCTACTACCTCCAGAACGGAAGGGATATGTATGTGGATCAAGAGTTGGATATCAACAGGCTCTCTGATTACGATGTTGATCATATCGTGCCACAGTCATTCTTGAAGGATGATTCTATCGATAACAAGGTGCTCACCAGGTCTGATAAGAACAGGGGTAAGAGTGATAACGTGCCAAGTGAAGAGGTTGTGAAGAAAATGAAGAACTATTGGAGGCAGCTCCTCAACGCTAAGCTCATCACTCAGAGAAAGTTCGATAACTTGACTAAGGCTGAGAGGGGAGGACTCTCTGAATTGGATAAGGCAGGATTCATCAAGAGGCAGCTTGTGGAAACCAGGCAGATCACTAAGCACGTTGCACAGATCCTCGATTCTAGGATGAACACCAAGTACGATGAGAACGATAAGTTGATCAGGGAAGTGAAGGTTATCACCCTCAAGTCAAAGCTCGTGTCTGATTTCAGAAAGGATTTCCAATTCTACAAGGTGAGGGAAATCAACAACTACCACCACGCTCACGATGCTTACCTTAACGCTGTTGTTGGAACCGCTCTCATCAAGAAGTATCCTAAGCTCGAGTCAGAGTTCGTGTACGGTGATTACAAGGTGTACGATGTGAGGAAGATGATCGCTAAGTCTGAGCAAGAGATCGGAAAGGCTACCGCTAAGTATTTCTTCTACTCTAACATCATGAATTTCTTCAAGACCGAGATTACCCTCGCTAACGGTGAGATCAGAAAGAGGCCACTCATCGAGACAAACGGTGAAACAGGTGAGATCGTGTGGGATAAGGGAAGGGATTTCGCTACCGTTAGAAAGGTGCTCTCTATGCCACAGGTGAACATCGTTAAGAAAACCGAGGTGCAGACCGGTGGATTCTCTAAAGAGTCTATCCTCCCTAAGAGGAACTCTGATAAGCTCATTGCTAGGAAGAAGGATTGGGACCCTAAGAAATACGGTGGTTTCGATTCTCCTACCGTGGCTTACTCTGTTCTCGTTGTGGCTAAGGTTGAGAAGGGAAAGAGTAAGAAGCTCAAGTCTGTTAAGGAACTTCTCGGAATCACTATCATGGAAAGGTCATCTTTCGAGAAGAACCCAATCGATTTCCTCGAGGCTAAGGGATACAAAGAGGTTAAGAAGGATCTCATCATCAAGCTCCCAAAGTACTCACTCTTCGAACTCGAGAACGGTAGAAAGAGGATGCTCGCTTCTGCTGGTGAGCTTCAAAAGGGAAACGAGCTTGCTCTCCCATCTAAGTACGTTAACTTTCTTTACCTCGCTTCTCACTACGAGAAGTTGAAGGGATCTCCAGAAGATAACGAGCAGAAGCAACTTTTCGTTGAGCAGCACAAGCACTACTTGGATGAGATCATCGAGCAGATCTCTGAGTTCTCTAAAAGGGTGATCCTCGCTGATGCAAACCTCGATAAGGTGTTGTCTGCTTACAACAAGCACAGAGATAAGCCTATCAGGGAACAGGCAGAGAACATCATCCATCTCTTCACCCTTACCAACCTCGGTGCTCCTGCTGCTTTCAAGTACTTCGATACAACCATCGATAGGAAGAGATACACCTCTACCAAAGAAGTGCTCGATGCTACCCTCATCCATCAGTCTATCACTGGACTCTACGAGACTAGGATCGATCTCTCACAGCTCGGTGGTGATTCAAGGGCTGATCCTAAGAAGAAGAGGAAGGTTTGA |
| Backbone | CACGAAGTGATCCGTTTAAACTATCAGTGTTTGACAGGATATATTGGCGGGTAAACCTAAGAGAAAAGAGCGTTTATTAGAATAATCGGATATTTAAAAGGGCGTGAAAAGGTTTATCCGTTCGTCCATTTGTATGTGCCAGCCGTGCGGCTGCATGAAATCCTGGCCGGTTTGTCTGATGCCAAGCTGGCGGCCTGGCCGGCCAGCTTGGCCGCTGAAGAAACCGAGCGCCGCCGTCTAAAAAGGTGATGTGTATTTGAGTAAAACAGCTTGCGTCATGCGGTCGCTGCGTATATGATGCGATGAGTAAATAAACAAATACGCAAGGGGAACGCATGAAGGTTATCGCTGTACTTAACCAGAAAGGCGGGTCAGGCAAGACGACCATCGCAACCCATCTAGCCCGCGCCCTGCAACTCGCCGGGGCCGATGTTCTGTTAGTCGATTCCGATCCCCAGGGCAGTGCCCGCGATTGGGCGGCCGTGCGGGAAGATCAACCGCTAACCGTTGTCGGCATCGACCGCCCGACGATTGACCGCGACGTGAAGGCCATCGGCCGGCGCGACTTCGTAGTGATCGACGGAGCGCCCCAGGCGGCGGACTTGGCTGTGTCCGCGATCAAGGCAGCCGACTTCGTGCTGATTCCGGTGCAGCCAAGCCCTTACGACATATGGGCCACCGCCGACCTGGTGGAGCTGGTTAAGCAGCGCATTGAGGTCACGGATGGAAGGCTACAAGCGGCCTTTGTCGTGTCGCGGGCGATCAAAGGCACGCGCATCGGCGGTGAGGTTGCCGAGGCGCTGGCCGGGTACGAGCTGCCCATTCTTGAGTCCCGTATCACGCAGCGCGTGAGCTACCCAGGCACTGCCGCCGCCGGCACAACCGTTCTTGAATCAGAACCCGAGGGCGACGCTGCCCGCGAGGTCCAGGCGCTGGCCGCTGAAATTAAATCAAAACTCATTTGAGTTAATGAGGTAAAGAGAAAATGAGCAAAAGCACAAACACGCTAAGTGCCGGCCGTCCGAGCGCACGCAGCAGCAAGGCTGCAACGTTGGCCAGCCTGGCAGACACGCCAGCCATGAAGCGGGTCAACTTTCAGTTGCCGGCGGAGGATCACACCAAGCTGAAGATGTACGCGGTACGCCAAGGCAAGACCATTACCGAGCTGCTATCTGAATACATCGCGCAGCTACCAGAGTAAATGAGCAAATGAATAAATGAGTAGATGAATTTTAGCGGCTAAAGGAGGCGGCATGGAAAATCAAGAACAACCAGGCACCGACGCCGTGGAATGCCCCATGTGTGGAGGAACGGGCGGTTGGCCAGGCGTAAGCGGCTGGGTTGTCTGCCGGCCCTGCAATGGCACTGGAACCCCCAAGCCCGAGGAATCGGCGTGACGGTCGCAAACCATCCGGCCCGGTACAAATCGGCGCGGCGCTGGGTGATGACCTGGTGGAGAAGTTGAAGGCCGCGCAGGCCGCCCAGCGGCAACGCATCGAGGCAGAAGCACGCCCCGGTGAATCGTGGCAAGCGGCCGCTGATCGAATCCGCAAAGAATCCCGGCAACCGCCGGCAGCCGGTGCGCCGTCGATTAGGAAGCCGCCCAAGGGCGACGAGCAACCAGATTTTTTCGTTCCGATGCTCTATGACGTGGGCACCCGCGATAGTCGCAGCATCATGGACGTGGCCGTTTTCCGTCTGTCGAAGCGTGACCGACGAGCTGGCGAGGTGATCCGCTACGAGCTTCCAGACGGGCACGTAGAGGTTTCCGCAGGGCCGGCCGGCATGGCCAGTGTGTGGGATTACGACCTGGTACTGATGGCGGTTTCCCATCTAACCGAATCCATGAACCGATACCGGGAAGGGAAGGGAGACAAGCCCGGCCGCGTGTTCCGTCCACACGTTGCGGACGTACTCAAGTTCTGCCGGCGAGCCGATGGCGGAAAGCAGAAAGACGACCTGGTAGAAACCTGCATTCGGTTAAACACCACGCACGTTGCCATGCAGCGTACGAAGAAGGCCAAGAACGGCCGCCTGGTGACGGTATCCGAGGGTGAAGCCTTGATTAGCCGCTACAAGATCGTAAAGAGCGAAACCGGGCGGCCGGAGTACATCGAGATCGAGCTAGCTGATTGGATGTACCGCGAGATCACAGAAGGCAAGAACCCGGACGTGCTGACGGTTCACCCCGATTACTTTTTGATCGATCCCGGCATCGGCCGTTTTCTCTACCGCCTGGCACGCCGCGCCGCAGGCAAGGCAGAAGCCAGATGGTTGTTCAAGACGATCTACGAACGCAGTGGCAGCGCCGGAGAGTTCAAGAAGTTCTGTTTCACCGTGCGCAAGCTGATCGGGTCAAATGACCTGCCGGAGTACGATTTGAAGGAGGAGGCGGGGCAGGCTGGCCCGATCCTAGTCATGCGCTACCGCAACCTGATCGAGGGCGAAGCATCCGCCGGTTCCTAATGTACGGAGCAGATGCTAGGGCAAATTGCCCTAGCAGGGGAAAAAGGTCGAAAAAGCTTCTTTCCTGTGGATAGCACGTACATTGGGAACCCAAAGCCGTACATTGGGAACCGGAACCCGTACATTGGGAACCCAAAGCCGTACATTGGGAACCGGTCACACATGTAAGTGACTGATATAAAAGAGAAAAAAGGCGATTTTTCCGCCTAAAACTCTTTAAAACTTATTAAAACTCTTAAAACCCGCCTGGCCTGTGCATAACTGTCTGGCCAGCGCACAGCCGAACAGCTGCAAAAAGCGCCTACCCTTCGGTCGCTGCGCTCCCTACGCCCCGCCGCTTCGCGTCGGCCTATCGCGGCCGCTGGCCGCTCAAAAATGGCTGGCCTACGGCCAGGCAATCTACCAGGGCGCGGACAAGCCGCGCCGTCGCCACTCGACCGCCGGCGCCCACATCAAGGCTCCGAGTGCGCGGAACCCCTATTTGTTTATTTTTCTAAATACATTCAAATATGTATCCGCTCATGAGACAATAACCCTGATAAATGCTTCAATAATATTGAAAAAGGAAGAGTATGGCTAAAATGAGAATATCACCGGAATTGAAAAAACTGATCGAAAAATACCGCTGCGTAAAAGATACGGAAGGAATGTCTCCTGCTAAGGTATATAAGCTGGTGGGAGAAAATGAAAACCTATATTTAAAAATGACGGACAGCCGGTATAAAGGGACCACCTATGATGTGGAACGGGAAAAGGACATGATGCTATGGCTGGAAGGAAAGCTGCCTGTTCCAAAGGTCCTGCACTTTGAACGGCATGATGGCTGGAGCAATCTGCTCATGAGTGAGGCCGATGGCGTCCTTTGCTCGGAAGAGTATGAAGATGAACAAAGCCCTGAAAAGATTATCGAGCTGTATGCGGAGTGCATCAGGCTCTTTCACTCCATCGACATATCGGATTGTCCCTATACGAATAGCTTAGACAGCCGCTTAGCCGAATTGGATTACTTACTGAATAACGATCTGGCCGATGTGGATTGCGAAAACTGGGAAGAGGACACTCCATTTAAAGATCCGCGCGAGCTGTATGATTTTTTAAAGACGGAAAAGCCCGAAGAGGAACTTGTCTTTTCCCACGGCGACCTGGGAGACAGCAACATCTTTGTGAAAGATGGCAAAGTAAGTGGCTTTATTGATCTTGGGAGAAGCGGCAGGGCGGACAAGTGGTATGACATTGCCTTCTGCGTCCGGTCGCTCAGGGAGGATATCGGGGAAGAACAGTATGTCGAGCTATTTTTTGACTTACTGGGGATCAAGCCTGATTGGGAGAAAATAAAATATTATATTTTACTGGATGAATTGTTTTAGCTGTCAGACCAAGTTTACTCATATATACTTTAGATTGATTTAAAACTTCATTTTTAATTTAAAAGGATCTAGGTGAAGATCCTTTTTGATAATCTCATGACCAAAATCCCTTAACGTGAGTTTTCGTTCCACTGAGCGTCAGACCCCGTAGAAAAGATCAAAGGATCTTCTTGAGATCCTTTTTTTCTGCGCGTAATCTGCTGCTTGCAAACAAAAAAACCACCGCTACCAGCGGTGGTTTGTTTGCCGGATCAAGAGCTACCAACTCTTTTTCCGAAGGTAACTGGCTTCAGCAGAGCGCAGATACCAAATACTGTTCTTCTAGTGTAGCCGTAGTTAGGCCACCACTTCAAGAACTCTGTAGCACCGCCTACATACCTCGCTCTGCTAATCCTGTTACCAGTGGCTGCTGCCAGTGGCGATAAGTCGTGTCTTACCGGGTTGGACTCAAGACGATAGTTACCGGATAAGGCGCAGCGGTCGGGCTGAACGGGGGGTTCGTGCACACAGCCCAGCTTGGAGCGAACGACCTACACCGAACTGAGATACCTACAGCGTGAGCTATGAGAAAGCGCCACGCTTCCCGAAGGGAGAAAGGCGGACAGGTATCCGGTAAGCGGCAGGGTCGGAACAGGAGAGCGCACGAGGGAGCTTCCAGGGGGAAACGCCTGGTATCTTTATAGTCCTGTCGGGTTTCGCCACCTCTGACTTGAGCGTCGATTTTTGTGATGCTCGTCAGGGGGGCGGAGCCTATGGAAAAACGCCAGCAACGCGGCCTTTTTACGGTTCCTGCTCGGATCTGTTGGACCGGACAGTAGTCATGGTTGATGGGCTGCCTGTATCGAGTGGTGATTTTGTGCCGAGCTGCCGGTCGGGGAGCTGTTGGCTGGCTGGTGGCAGGATATATTGTGGTGTAAACAAATTGACGCTTAGACAACTTAATAACACATTGCGGACGTTTTTAATGTACTGGGGTTGAACACTCT |
| ColE1 | TCATGACCAAAATCCCTTAACGTGAGTTTTCGTTCCACTGAGCGTCAGACCCCGTAGAAAAGATCAAAGGATCTTCTTGAGATCCTTTTTTTCTGCGCGTAATCTGCTGCTTGCAAACAAAAAAACCACCGCTACCAGCGGTGGTTTGTTTGCCGGATCAAGAGCTACCAACTCTTTTTCCGAAGGTAACTGGCTTCAGCAGAGCGCAGATACCAAATACTGTTCTTCTAGTGTAGCCGTAGTTAGGCCACCACTTCAAGAACTCTGTAGCACCGCCTACATACCTCGCTCTGCTAATCCTGTTACCAGTGGCTGCTGCCAGTGGCGATAAGTCGTGTCTTACCGGGTTGGACTCAAGACGATAGTTACCGGATAAGGCGCAGCGGTCGGGCTGAACGGGGGGTTCGTGCACACAGCCCAGCTTGGAGCGAACGACCTACACCGAACTGAGATACCTACAGCGTGAGCTATGAGAAAGCGCCACGCTTCCCGAAGGGAGAAAGGCGGACAGGTATCCGGTAAGCGGCAGGGTCGGAACAGGAGAGCGCACGAGGGAGCTTCCAGGGGGAAACGCCTGGTATCTTTATAGTCCTGTCGGGTTTCGCCACCTCTGACTTGAGCGTCGATTTTTGTGATGCTCGTCAGGGGGGCGGAGCCTATGGAAAAACGCCAGCAACGCGGCC |

**Supplementary Table 3.** Primers for amplification of ToMV loci and amplicon barcoding.

| **Primer name** | **Sequence (5'-end to 3'-end)** |
| --- | --- |
| FW-ToMV_primer | /5AmMC6/gcagtcgaacatgtagctgactcaggtcacTCAGCTTTTTAAGCCGTTCCG |
| RV-ToMV_primer | /5AmMC6/tggatcacttgtgcaagcatcacatcgtagTTGACAACGCAAAGGCCAAG |
| FW_BUP_primer | [16bp_barcode_sequence]gcagtcgaacatgtagctgactcaggtcac |
| RV_BUP_primer | [16bp_barcode_sequence]tggatcacttgtgcaagcatcacatcgtag |

**Supplementary Table 4.** Cross information and parental plant details for F1 seed generation.

| **TableI D** | **♀ plant information** | **♂ plant information** | **Crossing ID  (♀ x ♂)** | **Seeds sown** |
| --- | --- | --- | --- | --- |
| table_1 | MoneyBerg | CORE 1 771 Plant 16 | 10x16_1 | 4 |
| table_1 | MoneyBerg | CORE 1 771 Plant 02 | 10x2_1 | 22 |
| table_1 | MoneyBerg | CORE 1 771 Plant 02 | 10x2_2 | 84 |
| table_1 | MoneyBerg | CORE 1 771 Plant 02 | 10x2_3 | 32 |
| table_1 | MoneyBerg | CORE 1 771 Plant 10 | 11x13_1 | 112 |
| table_1 | MoneyBerg | WT Moneymaker | 11x38_1 | 156 |
| table_1 | MoneyBerg | CORE 1 771 Plant 10 | 12x13_1 | 10 |
| table_1 | MoneyBerg | CORE 1 771 Plant 10 | 12x13_2 | 8 |
| table_1 | MoneyBerg | CORE 1 771 Plant 10 | 12x13_3 | 10 |
| table_1 | MoneyBerg | CORE 1 771 Plant 4A | 12x6_1 | 8 |
| table_1 | MoneyBerg | CORE 1 771 Plant 02 | 13x2_1 | 88 |
| table_1 | MoneyBerg | CORE 1 771 Plant 02 | 13x2_2 | 90 |
| table_1 | MoneyBerg | CORE 1 771 Plant 02 | 13x2_3 | 152 |
| table_1 | MoneyBerg | CORE 1 771 Plant 02 | 14x2_1 | 6 |
| table_1 | MoneyBerg | CORE 1 771 Plant 02 | 14x2_2 | 26 |
| table_1 | MoneyBerg | CORE 1 771 Plant 4A | 14x6_1 | 10 |
| table_1 | MoneyBerg | CORE 1 771 Plant 4A | 14x6_2 | 8 |
| table_1 | MoneyBerg | CORE 1 771 Plant 4A | 14x6_3 | 6 |
| table_1 | MoneyBerg | CORE 1 771 Plant 6 | 15x10_1 | 30 |
| table_1 | MoneyBerg | CORE 1 771 Plant 02 | 16x2_1 | 162 |
| table_1 | MoneyBerg | CORE 1 771 Plant 02 | 16x2_2 | 12 |
| table_1 | MoneyBerg | CORE 1 771 Plant 02 | 16x2_3 | 8 |
| table_1 | MoneyBerg | CORE 1 771 Plant 02 | 16x2_4 | 34 |
| table_1 | MoneyBerg | CORE 1 771 Plant 6 | 17x10_1 | 14 |
| table_1 | MoneyBerg | CORE 1 771 Plant 6 | 17x10_2 | 16 |
| table_1 | MoneyBerg | CORE 1 771 Plant 16 | 17x16_1 | 16 |
| table_1 | MoneyBerg | CORE 1 771 Plant 02 | 17x2_1 | 86 |
| table_1 | MoneyBerg | CORE 1 771 Plant 02 | 17x2_2 | 158 |
| table_1 | MoneyBerg | CORE 1 771 Plant 02 | 17x2_3 | 12 |
| table_1 | MoneyBerg | CORE 1 771 Plant 02 | 18x2_1 | 74 |
| table_1 | MoneyBerg | CORE 1 771 Plant 02 | 18x2_2 | 16 |
| table_1 | MoneyBerg | CORE 1 771 Plant 02 | 18x2_3 | 30 |
| table_1 | MoneyBerg | CORE 1 771 Plant 02 | 18x2_4 | 86 |
| table_1 | MoneyBerg | CORE 1 HEM Plant 5 | 1x21_1 | 2 |
| table_1 | MoneyBerg | CORE 1 771 Plant 10 | 2x13_1 | 20 |
| table_1 | MoneyBerg | CORE 1 771 Plant 10 | 2x13_1 | 6 |
| table_1 | MoneyBerg | WT Moneymaker | 2x38_1 | 130 |
| table_1 | MoneyBERG- Cas9 plant 7 | CORE 2 TMV3 Plant 1 | 30x22_1 | 98 |
| table_1 | MoneyBERG- Cas9 plant 7 | CORE 2 TMV3 Plant 1 | 30x22_1 | 12 |
| table_1 | MoneyBERG- Cas9 plant 7 | CORE 2 TMV3 Plant 1 | 30x22_2 | 52 |
| table_1 | MoneyBERG- Cas9 plant 7 | CORE 2 TMV3 Plant 1 | 30x22_3 | 36 |
| table_1 | MoneyBERG- Cas9 plant 1 | CORE 2 TMV3 Plant 2 | 31x23_1 | 110 |
| table_1 | MoneyBERG- Cas9 plant 1 | CORE 2 TMV3 Plant 2 | 31x23_1 | 74 |
| table_1 | MoneyBERG- Cas9 plant 1 | CORE 2 TMV3 Plant 2 | 31x23_2 | 36 |
| table_1 | MoneyBERG- Cas9 plant 1 | CORE 2 TMV3 Plant 2 | 31x23_3 | 70 |
| table_1 | MoneyBERG- Cas9 plant 2 | CORE 2 TMV3 Plant 2 | 32x23_1 | 88 |
| table_1 | MoneyBERG- Cas9 plant 2 | CORE 2 TMV3 Plant 2 | 32x23_1 | 20 |
| table_1 | MoneyBERG- Cas9 plant 2 | CORE 2 1001 Plant 1 | 32x24_1 | 8 |
| table_1 | MoneyBERG- Cas9 plant 2 | CORE 2 1001 Plant 1 | 32x24_2 | 52 |
| table_1 | MoneyBERG- Cas9 plant 3 | CORE 2 TMV3 Plant 2 | 33x23_1 | 84 |
| table_1 | MoneyBERG- Cas9 plant 3 | CORE 2 1001 Plant 2 | 33x25_1 | 36 |
| table_1 | MoneyBERG- Cas9 plant 3 | CORE 2 1001 Plant 2 | 33x25_2 | 22 |
| table_1 | MoneyBERG- Cas9 plant 3 | CORE 2 1001 Plant 2 | 33x25_3 | 2 |
| table_1 | MoneyBERG- Cas9 plant 4 | CORE 2 TMV3 Plant 2 | 34x23_4 | 18 |
| table_1 | MoneyBERG- Cas9 plant 4 | CORE 2 1001 Plant 1 | 34x24_2 | 46 |
| table_1 | MoneyBERG- Cas9 plant 4 | CORE 2 1001 Plant 1 | 34x24_3 | 28 |
| table_1 | MoneyBERG- Cas9 plant 4 | CORE 2 1001 Plant 3 | 34x26_1 | 24 |
| table_1 | MoneyBERG- Cas9 plant 4 | CORE 2 1001 Plant 3 | 34x26_2 | 60 |
| table_1 | MoneyBERG- Cas9 plant 5 | CORE 2 TMV3 Plant 1 | 35x22_1 | 32 |
| table_1 | MoneyBERG- Cas9 plant 5 | CORE 2 TMV3 Plant 1 | 35x22_2 | 12 |
| table_1 | MoneyBERG- Cas9 plant 5 | CORE 2 TMV3 Plant 1 | 35x22_3 | 12 |
| table_1 | MoneyBERG- Cas9 plant 5 | CORE 2 1001 Plant 1 | 35x24_1 | 96 |
| table_1 | MoneyBERG- Cas9 plant 5 | CORE 2 1001 Plant 1 | 35x24_2 | 12 |
| table_1 | MoneyBERG- Cas9 plant 5 | CORE 2 1001 Plant 3 | 35x26_4 | 2 |
| table_1 | MoneyBERG- Cas9 plant 5 | CORE 2 1001 Plant 1 | 35x27_1 | 2 |
| table_1 | MoneyBERG- Cas9 plant 5 | WT Moneymaker | 35x39_1 | 38 |
| table_1 | MoneyBERG- Cas9 plant 5 | WT Moneymaker | 35x39_2 | 12 |
| table_1 | MoneyBERG- Cas9 plant 5 | WT Moneymaker | 35x39_3 | 6 |
| table_1 | MoneyBERG- Cas9 plant 6 | CORE 2 1001 Plant 2 | 36x25_1 | 86 |
| table_1 | MoneyBERG- Cas9 plant 6 | CORE 2 1001 Plant 2 | 36x25_2 | 20 |
| table_1 | MoneyBERG- Cas9 plant 6 | CORE 2 1001 Plant 2 | 36x25_3 | 26 |
| table_1 | MoneyBerg | CORE 1 771 Plant 6 | 3x10_1 | 4 |
| table_1 | MoneyBerg | CORE 1 771 Plant 10 | 3x13_1 | 160 |
| table_1 | MoneyBerg | CORE 1 771 Plant 10 | 3x13_2 | 12 |
| table_1 | MoneyBerg | CORE 1 771 Plant 4A | 3x6_1 | 26 |
| table_1 | MoneyBerg | CORE 1 771 Plant 4A | 3x6_2 | 4 |
| table_1 | MoneyBerg | CORE 1 771 Plant 4A | 3x6_3 | 8 |
| table_1 | MoneyBerg | CORE 1 771 Plant 6 | 4x10_1 | 4 |
| table_1 | MoneyBerg | WT Moneymaker | 4X38_1 | 130 |
| table_1 | MoneyBerg | CORE 1 771 Plant 4A | 4x6_1 | 62 |
| table_1 | MoneyBerg | CORE 1 771 Plant 4A | 4x6_2 | 32 |
| table_1 | MoneyBerg | CORE 1 771 Plant 4A | 4x6_3 | 16 |
| table_1 | MoneyBerg | CORE 1 771 Plant 6 | 5x10_1 | 2 |
| table_1 | MoneyBerg | CORE 1 771 Plant 6 | 5x10_2 | 10 |
| table_1 | MoneyBerg | CORE 1 771 Plant 10 | 5x13_1 | 6 |
| table_1 | MoneyBerg | CORE 1 771 Plant 10 | 5x13_2 | 12 |
| table_1 | MoneyBerg | CORE 1 771 Plant 10 | 5x13_3 | 8 |
| table_1 | MoneyBerg | CORE 1 771 Plant 6 | 6x10_1 | 52 |
| table_1 | MoneyBerg | CORE 1 771 Plant 4A | 6x6_1 | 16 |
| table_1 | MoneyBerg | CORE 1 771 Plant 4A | 6x6_2 | 12 |
| table_1 | MoneyBerg | WT Moneymaker | 7x38_1 | 138 |
| table_1 | MoneyBerg | CORE 1 771 Plant 4A | 7x6_1 | 52 |
| table_1 | MoneyBerg | CORE 1 771 Plant 4A | 7x6_2 | 14 |
| table_1 | MoneyBerg | CORE 1 HEM Plant 2 | 8x18_1 | 2 |
| table_1 | MoneyBerg | CORE 1 771 Plant 02 | 8x2_1 | 42 |
| table_1 | MoneyBerg | WT Moneymaker | 8x37_1 | 48 |
| table_1 | MoneyBerg | CORE 1 771 Plant 4A | 8x6_1 | 4 |
| table_1 | MoneyBerg | CORE 1 771 Plant 4A | 8x6_2 | 34 |
| table_2 | MoneyBerg | CORE 1 771 Plant 02 | 11x2_1 | 86 |
| table_2 | MoneyBerg | WT Moneymaker | 11x38_1 | 162 |
| table_2 | MoneyBerg | WT Moneymaker | 11x38_1 | 18 |
| table_2 | MoneyBerg | WT Moneymaker | 11x38_2 | 28 |
| table_2 | MoneyBerg | CORE 1 771 Plant 02 | 12x2_1 | 38 |
| table_2 | MoneyBerg | CORE 1 771 Plant 02 | 12x2_2 | 96 |
| table_2 | MoneyBerg | CORE 1 771 Plant 6 | 14x10_1 | 10 |
| table_2 | MoneyBerg | CORE 1 771 Plant 10 | 15x13_1 | 4 |
| table_2 | MoneyBerg | CORE 1 771 Plant 10 | 15x13_2 | 2 |
| table_2 | MoneyBerg | CORE 1 771 Plant 10 | 15x13_3 | 90 |
| table_2 | MoneyBerg | CORE 1 771 Plant 6 | 16x10_1 | 4 |
| table_2 | MoneyBerg | CORE 1 771 Plant 6 | 17x10_1 | 116 |
| table_2 | MoneyBerg | CORE 1 771 Plant 10 | 17x13_1 | 40 |
| table_2 | MoneyBerg | CORE 1 771 Plant 4A | 17x6_1 | 64 |
| table_2 | MoneyBerg | CORE 1 771 Plant 6 | 18x10_1 | 14 |
| table_2 | MoneyBerg | CORE 1 771 Plant 10 | 2x13_1 | 4 |
| table_2 | MoneyBerg | WT Moneymaker | 2x38_1 | 36 |
| table_2 | MoneyBerg | CORE 1 771 Plant 4A | 2x6_1 | 30 |
| table_2 | MoneyBerg | CORE 1 771 Plant 4A | 2x6_2 | 32 |
| table_2 | MoneyBERG- Cas9 plant 7 | CORE 2 TMV3 Plant 1 | 30x22_1 | 100 |
| table_2 | MoneyBERG- Cas9 plant 1 | CORE 2 TMV3 Plant 1 | 31x22_1 | 14 |
| table_2 | MoneyBERG- Cas9 plant 1 | CORE 2 TMV3 Plant 1 | 31x22_2 | 60 |
| table_2 | MoneyBERG- Cas9 plant 1 | CORE 2 1001 Plant 2 | 31x25_1 | 38 |
| table_2 | MoneyBERG- Cas9 plant 1 | WT Moneymaker | 31x40_1 | 70 |
| table_2 | MoneyBERG- Cas9 plant 1 | WT Moneymaker | 31x40_2 | 100 |
| table_2 | MoneyBERG- Cas9 plant 1 | WT Moneymaker | 31x40_3 | 84 |
| table_2 | MoneyBERG- Cas9 plant 2 | CORE 2 TMV3 Plant 1 | 32x22_1 | 124 |
| table_2 | MoneyBERG- Cas9 plant 2 | CORE 2 TMV3 Plant 1 | 32x22_2 | 32 |
| table_2 | MoneyBERG- Cas9 plant 3 | CORE 2 TMV3 Plant 1 | 33x22_1 | 78 |
| table_2 | MoneyBERG- Cas9 plant 3 | CORE 2 TMV3 Plant 1 | 33x22_2 | 122 |
| table_2 | MoneyBERG- Cas9 plant 4 | CORE 2 1001 Plant 2 | 34x25_1 | 102 |
| table_2 | MoneyBERG- Cas9 plant 4 | CORE 2 1001 Plant 2 | 34x25_1 | 92 |
| table_2 | MoneyBERG- Cas9 plant 4 | CORE 2 1001 Plant 2 | 34x25_1 | 50 |
| table_2 | MoneyBERG- Cas9 plant 5 | CORE 2 TMV3 Plant 1 | 35x22_1 | 94 |
| table_2 | MoneyBERG- Cas9 plant 5 | CORE 2 TMV3 Plant 2 | 35x23_1 | 94 |
| table_2 | MoneyBERG- Cas9 plant 5 | CORE 2 TMV3 Plant 2 | 35x23_2 | 76 |
| table_2 | MoneyBERG- Cas9 plant 5 | CORE 2 TMV3 Plant 2 | 35x23_3 | 114 |
| table_2 | MoneyBERG- Cas9 plant 5 | CORE 2 TMV3 Plant 2 | 35x23_4 | 40 |
| table_2 | MoneyBERG- Cas9 plant 5 | CORE 2 1001 Plant 2 | 35x25_1 | 54 |
| table_2 | MoneyBERG- Cas9 plant 6 | CORE 2 TMV3 Plant 1 | 36x22_1 | 132 |
| table_2 | MoneyBERG- Cas9 plant 6 | CORE 2 TMV3 Plant 2 | 36x23_1 | 12 |
| table_2 | MoneyBERG- Cas9 plant 6 | CORE 2 TMV3 Plant 2 | 36x23_1 | 144 |
| table_2 | MoneyBERG- Cas9 plant 6 | CORE 2 TMV3 Plant 2 | 36x23_1 | 122 |
| table_2 | MoneyBERG- Cas9 plant 6 | CORE 2 TMV3 Plant 2 | 36x23_2 | 74 |
| table_2 | MoneyBERG- Cas9 plant 6 | CORE 2 TMV3 Plant 2 | 36x23_3 | 62 |
| table_2 | MoneyBERG- Cas9 plant 6 | CORE 2 TMV3 Plant 2 | 36x23_4 | 68 |
| table_2 | MoneyBERG- Cas9 plant 6 | CORE 2 1001 Plant 2 | 36x25_1 | 80 |
| table_2 | MoneyBerg | CORE 1 771 Plant 6 | 4x10_2 | 64 |
| table_2 | MoneyBerg | CORE 1 771 Plant 6 | 4x10_2 | 24 |
| table_2 | MoneyBerg | WT Moneymaker | 5x38_1 | 12 |
| table_2 | MoneyBerg | WT Moneymaker | 5x38_1 | 6 |
| table_2 | MoneyBerg | WT Moneymaker | 5x38_2 | 144 |
| table_2 | MoneyBerg | CORE 1 771 Plant 4A | 5x6_1 | 22 |
| table_2 | MoneyBerg | CORE 1 771 Plant 6 | 6x10_1 | 86 |
| table_2 | MoneyBerg | WT Moneymaker | 7x38_1 | 82 |
| table_2 | MoneyBerg | WT Moneymaker | 7x38_1 | 146 |
| table_2 | MoneyBerg | WT Moneymaker | 7x38_2 | 82 |
| table_2 | MoneyBerg | CORE 1 771 Plant 6 | 8x10_1 | 72 |
| table_2 | MoneyBerg | CORE 1 771 Plant 4A | 9x6_1 | 22 |
| table_2 | MoneyBerg | CORE 1 771 Plant 4A | 9x6_2 | 8 |
| table_2 | MoneyBerg | CORE 1 771 Plant 4A | 9x6_3 | 70 |
| table_3 | MoneyBerg | CORE 1 771 Plant 4A | 10x6_1 | 66 |
| table_3 | MoneyBerg | CORE 1 771 Plant 4A | 10x6_2 | 20 |
| table_3 | MoneyBerg | CORE 1 771 Plant 10 | 11x13_1 | 14 |
| table_3 | MoneyBerg | CORE 1 771 Plant 10 | 11x13_2 | 42 |
| table_3 | MoneyBerg | CORE 1 771 Plant 4A | 11x6_1 | 6 |
| table_3 | MoneyBerg | CORE 1 771 Plant 4A | 11x6_1 | 2 |
| table_3 | MoneyBerg | CORE 1 771 Plant 4A | 11x6_2 | 4 |
| table_3 | MoneyBerg | CORE 1 771 Plant 4A | 15x6 | 84 |
| table_3 | MoneyBerg | CORE 1 771 Plant 4A | 15x6_1 | 2 |
| table_3 | MoneyBerg | CORE 1 771 Plant 4A | 15x6_1 | 102 |
| table_3 | MoneyBerg | CORE 1 771 Plant 02 | 16x2_1 | 66 |
| table_3 | MoneyBerg | CORE 1 771 Plant 6 | 17x10_2 | 22 |
| table_3 | MoneyBerg | CORE 1 771 Plant 02 | 17x2_1 | 78 |
| table_3 | MoneyBerg | CORE 1 771 Plant 10 | 2x13_1 | 30 |
| table_3 | MoneyBerg | CORE 1 771 Plant 10 | 2x13_2 | 8 |
| table_3 | MoneyBerg | WT Moneymaker | 2x38_1 | 12 |
| table_3 | MoneyBERG- Cas9 plant 7 | CORE 2 TMV3 Plant 1 | 30x22_1 | 50 |
| table_3 | MoneyBERG- Cas9 plant 7 | CORE 2 TMV3 Plant 2 | 30x23_1 | 120 |
| table_3 | MoneyBERG- Cas9 plant 7 | WT Moneymaker | 30x39_1 | 236 |
| table_3 | MoneyBERG- Cas9 plant 7 | WT Moneymaker | 30x40_1 | 46 |
| table_3 | MoneyBERG- Cas9 plant 7 | WT Moneymaker | 30x40_2 | 54 |
| table_3 | MoneyBERG- Cas9 plant 7 | WT Moneymaker | 30x40_3 | 94 |
| table_3 | MoneyBERG- Cas9 plant 7 | WT Moneymaker | 30x40_4 | 6 |
| table_3 | MoneyBERG- Cas9 plant 7 | WT Moneymaker | 30x40_5 | 48 |
| table_3 | MoneyBERG- Cas9 plant 7 | WT Moneymaker | 30x40_6 | 82 |
| table_3 | MoneyBERG- Cas9 plant 7 | WT Moneymaker | 30x40_7 | 20 |
| table_3 | MoneyBERG- Cas9 plant 1 | CORE 2 TMV3 Plant 1 | 31x22_1 | 16 |
| table_3 | MoneyBERG- Cas9 plant 1 | CORE 2 TMV3 Plant 1 | 31x22_2 | 26 |
| table_3 | MoneyBERG- Cas9 plant 1 | CORE 2 TMV3 Plant 2 | 31x23_1 | 16 |
| table_3 | MoneyBERG- Cas9 plant 1 | CORE 2 TMV3 Plant 2 | 31x23_2 | 4 |
| table_3 | MoneyBERG- Cas9 plant 1 | CORE 2 TMV3 Plant 2 | 31x23_3 | 18 |
| table_3 | MoneyBERG- Cas9 plant 1 | CORE 2 TMV3 Plant 2 | 31x23_4 | 28 |
| table_3 | MoneyBERG- Cas9 plant 1 | CORE 2 TMV3 Plant 2 | 31x23_5 | 4 |
| table_3 | MoneyBERG- Cas9 plant 1 | CORE 2 TMV3 Plant 2 | 31x23_6 | 42 |
| table_3 | MoneyBERG- Cas9 plant 1 | CORE 2 TMV3 Plant 2 | 31x23_7 | 36 |
| table_3 | MoneyBERG- Cas9 plant 2 | CORE 2 TMV3 Plant 2 | 32x23_1 | 96 |
| table_3 | MoneyBERG- Cas9 plant 2 | CORE 2 1001 Plant 1 | 32x24_1 | 120 |
| table_3 | MoneyBERG- Cas9 plant 2 | CORE 2 1001 Plant 1 | 32x24_1 | 2 |
| table_3 | MoneyBERG- Cas9 plant 2 | WT Moneymaker | 32x40_2 | 74 |
| table_3 | MoneyBERG- Cas9 plant 2 | WT Moneymaker | 32x40_3 | 38 |
| table_3 | MoneyBERG- Cas9 plant 3 | CORE 2 TMV3 Plant 1 | 33x22_1 | 128 |
| table_3 | MoneyBERG- Cas9 plant 3 | CORE 2 TMV3 Plant 2 | 33x23_1 | 90 |
| table_3 | MoneyBERG- Cas9 plant 3 | CORE 2 1001 Plant 2 | 33x25_1 | 70 |
| table_3 | MoneyBERG- Cas9 plant 3 | CORE 2 1001 Plant 2 | 33x25_1 | 100 |
| table_3 | MoneyBERG- Cas9 plant 3 | WT Moneymaker | 33x39_1 | 16 |
| table_3 | MoneyBERG- Cas9 plant 3 | WT Moneymaker | 33x40_1 | 12 |
| table_3 | MoneyBERG- Cas9 plant 4 | CORE 2 1001 Plant 1 | 34x24_1 | 120 |
| table_3 | MoneyBERG- Cas9 plant 4 | CORE 2 1001 Plant 2 | 34x25_1 | 10 |
| table_3 | MoneyBERG- Cas9 plant 4 | CORE 2 1001 Plant 2 | 34x25_2 | 4 |
| table_3 | MoneyBERG- Cas9 plant 4 | CORE 2 1001 Plant 2 | 34x25_3 | 4 |
| table_3 | MoneyBERG- Cas9 plant 4 | CORE 2 1001 Plant 2 | 34x25_4 | 2 |
| table_3 | MoneyBERG- Cas9 plant 4 | CORE 2 1001 Plant 2 | 34x25_5 | 12 |
| table_3 | MoneyBERG- Cas9 plant 4 | CORE 2 1001 Plant 2 | 34x25_6 | 6 |
| table_3 | MoneyBERG- Cas9 plant 4 | CORE 2 1001 Plant 2 | 34x25_7 | 18 |
| table_3 | MoneyBERG- Cas9 plant 4 | CORE 2 1001 Plant 2 | 34x25_7 | 26 |
| table_3 | MoneyBERG- Cas9 plant 4 | WT Moneymaker | 34x40_1 | 94 |
| table_3 | MoneyBERG- Cas9 plant 4 | WT Moneymaker | 34x40_1 | 18 |
| table_3 | MoneyBERG- Cas9 plant 5 | CORE 2 TMV3 Plant 1 | 35x22_1 | 72 |
| table_3 | MoneyBERG- Cas9 plant 5 | CORE 2 TMV3 Plant 1 | 35x22_1 | 78 |
| table_3 | MoneyBERG- Cas9 plant 5 | CORE 2 TMV3 Plant 1 | 35x22_2 | 78 |
| table_3 | MoneyBERG- Cas9 plant 5 | CORE 2 TMV3 Plant 1 | 35x22_3 | 68 |
| table_3 | MoneyBERG- Cas9 plant 5 | CORE 2 TMV3 Plant 2 | 35x23_1 | 80 |
| table_3 | MoneyBERG- Cas9 plant 5 | CORE 2 TMV3 Plant 2 | 35x23_2 | 4 |
| table_3 | MoneyBERG- Cas9 plant 5 | CORE 2 1001 Plant 1 | 35x24_1 | 46 |
| table_3 | MoneyBERG- Cas9 plant 6 | CORE 2 TMV3 Plant 1 | 36x22_1 | 86 |
| table_3 | MoneyBERG- Cas9 plant 6 | CORE 2 TMV3 Plant 1 | 36x22_2 | 40 |
| table_3 | MoneyBERG- Cas9 plant 6 | CORE 2 TMV3 Plant 2 | 36x23_1 | 4 |
| table_3 | MoneyBERG- Cas9 plant 6 | CORE 2 TMV3 Plant 2 | 36x23_2 | 30 |
| table_3 | MoneyBERG- Cas9 plant 6 | CORE 2 TMV3 Plant 2 | 36x23_3 | 14 |
| table_3 | MoneyBERG- Cas9 plant 6 | CORE 2 TMV3 Plant 2 | 36x23_4 | 2 |
| table_3 | MoneyBERG- Cas9 plant 6 | CORE 2 TMV3 Plant 2 | 36x23_5 | 116 |
| table_3 | MoneyBERG- Cas9 plant 6 | CORE 2 TMV3 Plant 2 | 36x23_6 | 98 |
| table_3 | MoneyBERG- Cas9 plant 6 | CORE 2 1001 Plant 2 | 36x25_1 | 26 |
| table_3 | MoneyBERG- Cas9 plant 6 | CORE 2 1001 Plant 2 | 36x25_2 | 16 |
| table_3 | MoneyBERG- Cas9 plant 6 | CORE 2 1001 Plant 2 | 36x25_3 | 64 |
| table_3 | MoneyBERG- Cas9 plant 6 | WT Moneymaker | 36x39_1 | 18 |
| table_3 | MoneyBERG- Cas9 plant 6 | WT Moneymaker | 36x39_1 | 12 |
| table_3 | MoneyBERG- Cas9 plant 6 | WT Moneymaker | 36x40_1 | 50 |
| table_3 | MoneyBERG- Cas9 plant 6 | WT Moneymaker | 36x40_2 | 8 |
| table_3 | MoneyBerg | CORE 1 771 Plant 4A | 4x6_2 | 38 |
| table_3 | MoneyBerg | CORE 1 771 Plant 4A | 4x6_3 | 22 |
| table_3 | MoneyBerg | CORE 1 771 Plant 4A | 4x6_4 | 40 |
| table_3 | MoneyBerg | CORE 1 771 Plant 6 | 6x10_1 | 16 |
| table_3 | MoneyBerg | CORE 1 771 Plant 6 | 6x10_2 | 12 |
| table_3 | MoneyBerg | CORE 1 771 Plant 6 | 6x10_3 | 32 |
| table_3 | MoneyBerg | CORE 1 771 Plant 6 | 6x10_4 | 78 |
| table_3 | MoneyBerg | CORE 1 771 Plant 6 | 6x10_5 | 56 |
| table_3 | MoneyBerg | CORE 1 771 Plant 4A | 7x6_1 | 2 |

**Supplementary Table 5.** PCR reaction parameters and mix composition for ToMV allele enrichment.

| **PCR 1 ToMV allele enrichment** | | | |  |
| --- | --- | --- | --- | --- |
|  |  |  |  |  |
| Phire Hot Start II DNA Polymerase PCR Cycing settings | | | |  |
|  |  |  |  |  |
| **Cycle step** | **Temp** | **Time** | **Cycles** |  |
| Initial denaturation | 98 | 30 | 1 |  |
| Denaturation | 98 | 15 | 22 |  |
| Annealing | 64.3 | 15 | 22 |  |
| Extension | 72 | 180 | 22 |  |
| Final extension | 72 | 600 | 1 |  |

| Phire Hot Start II DNA Polymerase | Per 20 μL |
| --- | --- |
| Reaction Mix | reaction |
| Nucease-Free water | 12.6 |
| 5 x Phire Reaction Buffer | 4 |
| dNTPs (5mM stock) | 0.8 |
| FW Primer (100 mM stock) | 0.1 |
| RV Primer (100 mM stock) | 0.1 |
| DNA (80 ng) | 2 |
| Phire Polymerase | 0.4 |

**Supplementary Table 6.** PCR reaction parameters and mix composition for barcode addition using barcoded universal primers.

| Phire Hot Start II DNA Polymerase | Per 10 μL |
| --- | --- |
| Reaction Mix | reaction |
| Nucease-Free water | 6.8 |
| 5 x Phire Reaction Buffer | 4 |
| dNTPs (5mM stock) | 0.8 |
| FW Primer (10 mM stock) | 2 |
| RV Primer (10 mM stock) | 2 |
| Ampure XP cleaned PCR 1 product | 4 |
| Phire Polymerase | 0.4 |

| **PCR 2 Barcoded Universal Primer addition** | | | |  |
| --- | --- | --- | --- | --- |
|  |  |  |  |  |
| Phire Hot Start II DNA Polymerase PCR Cycing settings | | | |  |
|  |  |  |  |  |
| **Cycle step** | **Temp** | **Time** | **Cycles** |  |
| Initial denaturation | 98 | 30 | 1 |  |
| Denaturation | 98 | 15 | 2 |  |
| Annealing | 68 | 15 | 2 |  |
| Extension | 72 | 180 | 2 |  |
| Final extension | 72 | 600 | 1 |  |

**Supplementary Table 7.** *S. pimpinellifolium*, *S. lycopersicum* cv. Moneymaker and cv. Moneyberg haplotype sequences.

| **Genotype** | **Haplotype pattern** |
| --- | --- |
| *S. pimpinellifolium* | MMMMMMMMMBMMMMMMMMMMMMMMMMMMMMMMMMMMMMMMMMMMMMMMMBMMMMMMMMMMMM |
| *S. lycopersicum* cv. Moneymaker | MMMMMMMMMMMMMMMMMMMMMMMMMMMMMMMMMMMMMMMMMMMMMMMMMMMMMMMMMMMMMM |
| *S. lycopersicum* cv. Moneyberg | BBBBBBBBBBBBBBBBBBBBBBBBBBBBBBBBBBBBBBBBBBBBBBBBBBBBBBBBBBBBBB |

**Supplementary Table 8.** Frequency of CRISPR/Cas9-induced indels in F1 seedlings across column pools.

| **Column pool name*** | **Expected target site** | **Number of analysed reads** | **% of reads with mutations at gRNA1-HDR** | **% of reads with mutations at gRNA2-HDR/NHEJ** | **% of reads with mutations gRNA3-tm2-HDR** |
| --- | --- | --- | --- | --- | --- |
| 1_A1 | gRNA1-HDR | 3893 | 46.85 | 0.08 | 0.39 |
| 1_A10 | gRNA1-HDR | 3954 | 51.44 | 0.15 | 0.35 |
| 1_A11 | gRNA1-HDR | 2883 | 49.01 | 0.42 | 0.42 |
| 1_A12 | gRNA1-HDR | 3497 | 67.49 | 0.26 | 0.37 |
| 1_A13 | gRNA1-HDR | 3190 | 67.84 | 0.16 | 0.47 |
| 1_A14 | gRNA1-HDR | 2731 | 63.86 | 0.29 | 0.44 |
| 1_A15 | gRNA1-HDR | 2778 | 65.62 | 0.14 | 0.40 |
| 1_A16 | gRNA1-HDR | 2801 | 67.80 | 0.36 | 0.46 |
| 1_A17 | gRNA1-HDR | 3121 | 63.79 | 0.13 | 0.42 |
| 1_A18 | gRNA1-HDR | 3289 | 42.63 | 0.12 | 0.49 |
| 1_A19 | gRNA1-HDR | 3392 | 44.10 | 0.18 | 0.44 |
| 1_A2 | gRNA1-HDR | 2471 | 42.82 | 0.32 | 0.65 |
| 1_A20 | gRNA1-HDR | 2818 | 36.73 | 0.25 | 0.18 |
| 1_A21 | gRNA1-HDR | 3003 | 55.14 | 0.30 | 0.43 |
| 1_A3 | gRNA1-HDR | 2995 | 39.60 | 0.27 | 0.23 |
| 1_A4 | gRNA1-HDR | 3368 | 38.95 | 0.09 | 0.36 |
| 1_A5 | gRNA1-HDR | 3503 | 39.25 | 0.17 | 0.26 |
| 1_A6 | gRNA1-HDR | 3858 | 48.63 | 0.26 | 0.41 |
| 1_A7 | gRNA1-HDR | 4180 | 44.93 | 0.19 | 0.31 |
| 1_A8 | gRNA1-HDR | 3707 | 31.18 | 0.22 | 0.46 |
| 1_A9 | gRNA1-HDR | 3093 | 35.05 | 0.26 | 0.45 |
| 1_B1 | gRNA1-HDR | 3843 | 34.09 | 0.21 | 0.34 |
| 1_B10 | gRNA1-HDR | 3231 | 45.13 | 0.19 | 0.50 |
| 1_B11 | gRNA1-HDR | 3398 | 41.14 | 0.21 | 0.44 |
| 1_B12 | gRNA1-HDR | 3186 | 37.57 | 0.13 | 0.25 |
| 1_B13 | gRNA1-HDR | 2969 | 41.53 | 0.24 | 0.30 |
| 1_B14 | gRNA1-HDR | 2909 | 41.32 | 0.24 | 0.21 |
| 1_B15 | gRNA1-HDR | 3973 | 31.11 | 0.23 | 0.40 |
| 1_B16 | gRNA1-HDR | 2843 | 28.00 | 0.35 | 0.35 |
| 1_B17 | gRNA1-HDR | 3440 | 41.31 | 0.17 | 0.35 |
| 1_B18 | gRNA1-HDR | 3096 | 44.67 | 0.19 | 0.45 |
| 1_B19 | gRNA1-HDR | 2309 | 60.63 | 0.17 | 0.39 |
| 1_B2 | gRNA1-HDR | 3068 | 32.37 | 0.16 | 0.46 |
| 1_B20 | gRNA1-HDR | 2558 | 69.31 | 0.20 | 0.31 |
| 1_B21 | gRNA1-HDR | 2078 | 69.01 | 0.29 | 0.29 |
| 1_B3 | gRNA1-HDR | 3768 | 39.20 | 0.29 | 0.37 |
| 1_B4 | gRNA1-HDR | 2642 | 40.54 | 0.26 | 0.26 |
| 1_B5 | gRNA1-HDR | 2522 | 41.12 | 0.36 | 0.40 |
| 1_B6 | gRNA1-HDR | 2690 | 41.08 | 0.52 | 0.45 |
| 1_B7 | gRNA1-HDR | 2097 | 36.72 | 0.38 | 0.29 |
| 1_B8 | gRNA1-HDR | 3119 | 47.13 | 0.35 | 0.51 |
| 1_B9 | gRNA1-HDR | 2936 | 37.23 | 0.17 | 0.31 |
| 1_C1 | gRNA1-HDR | 2143 | 61.41 | 0.23 | 0.19 |
| 1_C10 | WT | 2611 | 0.23 | 0.23 | 0.34 |
| 1_C11 | WT (6) gRNA3-tm2-HDR (15) | 2216 | 0.05 | 0.23 | 4.29 |
| 1_C12 | gRNA3-tm2-HDR | 2897 | 0.07 | 0.38 | 3.35 |
| 1_C13 | gRNA3-tm2-HDR | 7282 | 0.03 | 0.26 | 1.06 |
| 1_C14 | gRNA3-tm2-HDR | 1090 | 0.18 | 0.18 | 0.64 |
| 1_C15 | gRNA3-tm2-HDR | 2244 | 0.18 | 0.22 | 0.45 |
| 1_C16 | gRNA3-tm2-HDR | 509 | 0.00 | 0.20 | 0.98 |
| 1_C17 | gRNA3-tm2-HDR | 2073 | 0.00 | 0.19 | 0.72 |
| 1_C18 | gRNA3-tm2-HDR | 1925 | 0.10 | 0.26 | 0.47 |
| 1_C19 | gRNA3-tm2-HDR | 1674 | 0.06 | 0.12 | 0.72 |
| 1_C2 | gRNA1-HDR | 2727 | 51.34 | 0.51 | 0.40 |
| 1_C20 | gRNA3-tm2-HDR | 1307 | 0.00 | 0.08 | 0.38 |
| 1_C21 | gRNA3-tm2-HDR (2) gRNA1-HDR (19) | 1729 | 0.00 | 0.23 | 0.23 |
| 1_C3 | gRNA1-HDR | 528 | 20.27 | 0.19 | 0.19 |
| 1_C4 | gRNA1-HDR (6) WT (15) | 2743 | 0.15 | 0.18 | 0.29 |
| 1_C5 | WT | 2630 | 0.23 | 0.34 | 0.34 |
| 1_C6 | WT | 2589 | 0.19 | 0.31 | 0.39 |
| 1_C7 | WT | 3148 | 0.19 | 0.32 | 0.44 |
| 1_C8 | WT | 2399 | 0.17 | 0.13 | 0.46 |
| 1_C9 | WT | 3321 | 1.08 | 0.06 | 0.27 |
| 1_D_1 | gRNA1-HDR | 1352 | 46.15 | 0.00 | 0.37 |
| 1_D_10 | WT (10) gRNA3-tm2-HDR (11) | 2415 | 0.83 | 0.12 | 10.52 |
| 1_D_11 | gRNA3-tm2-HDR | 2421 | 0.08 | 0.25 | 2.44 |
| 1_D_12 | gRNA3-tm2-HDR | 2505 | 0.12 | 0.40 | 6.47 |
| 1_D_13 | gRNA3-tm2-HDR | 2580 | 0.12 | 0.43 | 4.84 |
| 1_D_14 | gRNA3-tm2-HDR | 2419 | 1.28 | 4.59 | 0.45 |
| 1_D_15 | gRNA3-tm2-HDR | 2186 | 0.27 | 0.23 | 0.23 |
| 1_D_16 | gRNA3-tm2-HDR | 114 | 0.00 | 0.00 | 0.00 |
| 1_D_17 | gRNA3-tm2-HDR (9) gRNA2-HDR/NHEJ (12) | 2441 | 0.29 | 2.09 | 0.37 |
| 1_D_18 | gRNA2-HDR/NHEJ | 2390 | 0.21 | 29.16 | 0.13 |
| 1_D_19 | gRNA2-HDR/NHEJ | 1973 | 0.00 | 9.73 | 0.66 |
| 1_D_2 | gRNA1-HDR (5) WT (16) | 1605 | 33.02 | 0.31 | 0.50 |
| 1_D_20 | gRNA2-HDR/NHEJ | 2617 | 0.08 | 21.02 | 0.57 |
| 1_D_21 | gRNA2-HDR/NHEJ | 2755 | 0.18 | 13.03 | 0.25 |
| 1_D_3 | WT | 1726 | 0.29 | 0.29 | 0.29 |
| 1_D_4 | WT | 1763 | 0.23 | 0.11 | 0.40 |
| 1_D_5 | WT | 2141 | 0.05 | 0.23 | 0.51 |
| 1_D_6 | WT | 1827 | 0.16 | 0.27 | 0.33 |
| 1_D_7 | WT | 1538 | 0.13 | 0.39 | 0.33 |
| 1_D_8 | WT | 2491 | 0.00 | 0.28 | 0.28 |
| 1_D_9 | WT | 767 | 0.00 | 0.26 | 0.26 |
| 1_E_1 | gRNA2-HDR/NHEJ | 1720 | 0.06 | 14.53 | 0.58 |
| 1_E_10 | gRNA1-HDR (3) WT (18) | 1654 | 3.87 | 0.18 | 0.30 |
| 1_E_2 | gRNA2-HDR/NHEJ | 1122 | 0.00 | 16.49 | 0.62 |
| 1_E_3 | gRNA2-HDR/NHEJ | 1715 | 6.36 | 52.48 | 0.41 |
| 1_E_4 | gRNA2-HDR/NHEJ | 1918 | 0.16 | 30.55 | 0.47 |
| 1_E_5 | gRNA2-HDR/NHEJ | 1840 | 0.16 | 21.20 | 0.33 |
| 1_E_6 | gRNA2-HDR/NHEJ | 1785 | 0.11 | 18.71 | 0.28 |
| 1_E_7 | gRNA2-HDR/NHEJ | 1345 | 0.07 | 6.47 | 0.22 |
| 1_E_8 | gRNA2-HDR/NHEJ | 1869 | 0.16 | 0.16 | 0.37 |
| 1_E_9 | gRNA2-HDR/NHEJ (10) WT (11) | 1653 | 0.06 | 0.24 | 1.88 |
| 2_A1 | gRNA1-HDR | 3742 | 37.49 | 0.24 | 0.51 |
| 2_A10 | gRNA1-HDR | 3296 | 52.18 | 0.12 | 0.46 |
| 2_A11 | gRNA1-HDR | 3398 | 20.34 | 0.21 | 0.35 |
| 2_A12 | gRNA1-HDR | 3368 | 33.17 | 0.30 | 0.42 |
| 2_A13 | gRNA1-HDR | 4161 | 32.80 | 0.17 | 0.58 |
| 2_A14 | gRNA1-HDR | 2648 | 40.41 | 0.26 | 0.42 |
| 2_A15 | gRNA1-HDR | 3255 | 45.75 | 0.28 | 0.52 |
| 2_A16 | gRNA1-HDR | 3045 | 45.22 | 0.10 | 0.39 |
| 2_A17 | gRNA1-HDR | 2323 | 48.13 | 0.09 | 0.26 |
| 2_A18 | gRNA1-HDR (9) WT (12) | 3858 | 35.41 | 0.29 | 0.54 |
| 2_A2 | gRNA1-HDR | 2999 | 42.61 | 0.30 | 0.63 |
| 2_A20 | WT | 3510 | 0.23 | 0.17 | 0.34 |
| 2_A21 | WT | 3330 | 0.12 | 0.18 | 0.57 |
| 2_A3 | gRNA1-HDR | 3960 | 48.56 | 0.23 | 0.40 |
| 2_A4 | gRNA1-HDR | 3607 | 27.72 | 0.22 | 0.55 |
| 2_A5 | gRNA1-HDR | 5911 | 39.10 | 0.25 | 0.24 |
| 2_A6 | gRNA1-HDR | 3397 | 60.05 | 0.15 | 0.47 |
| 2_A7 | gRNA1-HDR | 3080 | 71.20 | 0.36 | 0.42 |
| 2_A8 | gRNA1-HDR | 3403 | 68.03 | 0.21 | 0.59 |
| 2_A9 | gRNA1-HDR | 3566 | 63.85 | 0.31 | 0.56 |
| 2_B1 | WT | 2191 | 0.09 | 0.32 | 0.32 |
| 2_B10 | gRNA3-tm2-HDR | 3780 | 0.32 | 0.34 | 0.93 |
| 2_B11 | gRNA3-tm2-HDR | 1799 | 0.00 | 0.39 | 0.39 |
| 2_B12 | gRNA3-tm2-HDR | 3555 | 0.08 | 0.23 | 0.42 |
| 2_B13 | gRNA3-tm2-HDR | 2509 | 0.12 | 0.20 | 0.56 |
| 2_B14 | gRNA3-tm2-HDR | 3121 | 0.00 | 0.42 | 0.42 |
| 2_B15 | gRNA3-tm2-HDR (5) gRNA2-HDR/NHEJ (16) | 3704 | 0.08 | 54.16 | 0.46 |
| 2_B16 | gRNA2-HDR/NHEJ | 3244 | 0.28 | 29.07 | 0.28 |
| 2_B17 | gRNA2-HDR/NHEJ | 3259 | 0.15 | 45.11 | 0.25 |
| 2_B18 | gRNA2-HDR/NHEJ | 3728 | 0.00 | 7.54 | 0.40 |
| 2_B19 | gRNA2-HDR/NHEJ | 1896 | 0.11 | 0.32 | 0.37 |
| 2_B2 | WT | 2658 | 0.08 | 0.41 | 0.30 |
| 2_B20 | gRNA2-HDR/NHEJ | 1991 | 0.15 | 15.67 | 0.65 |
| 2_B21 | gRNA2-HDR/NHEJ | 2 | 0.00 | 50.00 | 50.00 |
| 2_B3 | WT | 3368 | 0.03 | 0.24 | 0.45 |
| 2_B4 | WT (13) gRNA3-tm2-HDR (8) | 2747 | 0.15 | 0.18 | 3.64 |
| 2_B5 | gRNA3-tm2-HDR | 3358 | 0.12 | 0.39 | 6.94 |
| 2_B6 | gRNA3-tm2-HDR | 2309 | 0.09 | 0.30 | 9.44 |
| 2_B7 | gRNA3-tm2-HDR | 3142 | 0.16 | 0.29 | 5.76 |
| 2_B8 | gRNA3-tm2-HDR | 3578 | 0.14 | 0.25 | 8.41 |
| 2_B9 | gRNA3-tm2-HDR | 2992 | 0.10 | 0.23 | 13.44 |
| 2_C1 | WT | 2390 | 0.08 | 0.08 | 0.50 |
| 2_C10 | gRNA3-tm2-HDR | 20 | 0.00 | 0.00 | 5.00 |
| 2_C11 | gRNA3-tm2-HDR | 33 | 0.00 | 0.00 | 6.06 |
| 2_C12 | gRNA3-tm2-HDR | 3028 | 0.03 | 0.17 | 3.93 |
| 2_C13 | gRNA3-tm2-HDR | 2051 | 0.15 | 0.24 | 7.41 |
| 2_C14 | gRNA3-tm2-HDR | 1559 | 0.13 | 0.45 | 0.32 |
| 2_C15 | gRNA3-tm2-HDR | 1652 | 0.18 | 0.36 | 0.30 |
| 2_C16 | gRNA3-tm2-HDR | 2036 | 0.05 | 0.20 | 0.49 |
| 2_C17 | gRNA3-tm2-HDR | 2090 | 0.00 | 0.33 | 0.57 |
| 2_C18 | gRNA3-tm2-HDR | 1901 | 0.16 | 0.16 | 0.37 |
| 2_C19 | gRNA3-tm2-HDR | 1776 | 0.06 | 0.11 | 0.39 |
| 2_C2 | WT | 2554 | 0.04 | 0.20 | 0.55 |
| 2_C20 | gRNA3-tm2-HDR | 2809 | 0.21 | 0.25 | 0.25 |
| 2_C21 | gRNA3-tm2-HDR | 34 | 0.00 | 0.00 | 0.00 |
| 2_C3 | WT | 2555 | 0.20 | 0.51 | 0.55 |
| 2_C4 | WT | 2464 | 0.16 | 0.28 | 0.53 |
| 2_C5 | WT | 2729 | 0.07 | 0.22 | 0.29 |
| 2_C6 | WT | 2158 | 0.05 | 0.28 | 0.37 |
| 2_C7 | WT (3) gRNA3-tm2-HDR (18) | 2024 | 0.10 | 0.25 | 5.24 |
| 2_C8 | gRNA3-tm2-HDR | 2625 | 0.15 | 0.27 | 7.70 |
| 2_C9 | gRNA3-tm2-HDR | 2487 | 0.08 | 0.16 | 8.89 |
| 2_D1 | WT | 2790 | 0.07 | 0.25 | 0.57 |
| 2_D10 | WT (11) gRNA3-tm2-HDR (10) | 37 | 2.70 | 0.00 | 0.00 |
| 2_D11 | WT (7) gRNA1-HDR (14) | 2527 | 26.08 | 0.32 | 0.47 |
| 2_D12 | gRNA1-HDR | 2287 | 44.64 | 0.44 | 0.39 |
| 2_D13 | gRNA1-HDR gRNA2-HDR/NHEJ | 2982 | 8.72 | 8.01 | 0.37 |
| 2_D14 | gRNA2-HDR/NHEJ | 2891 | 0.14 | 17.50 | 0.48 |
| 2_D15 | gRNA2-HDR/NHEJ (7) gRNA1-HDR (14) | 3245 | 38.64 | 7.89 | 0.46 |
| 2_D16 | gRNA1-HDR | 2891 | 36.32 | 0.14 | 0.59 |
| 2_D17 | gRNA1-HDR | 3023 | 40.92 | 0.23 | 0.33 |
| 2_D18 | gRNA1-HDR (3) WT (18) | 2436 | 6.86 | 0.16 | 0.33 |
| 2_D19 | WT | 1840 | 0.16 | 0.22 | 0.43 |
| 2_D2 | WT | 2893 | 0.31 | 0.21 | 0.52 |
| 2_D20 | WT | 3210 | 0.06 | 0.25 | 0.40 |
| 2_D21 | WT | 2795 | 0.11 | 0.14 | 0.32 |
| 2_D3 | WT | 2809 | 0.04 | 0.43 | 0.50 |
| 2_D4 | WT | 30 | 0.00 | 0.00 | 0.00 |
| 2_D5 | WT | 2215 | 0.00 | 0.18 | 0.50 |
| 2_D6 | WT | 2847 | 0.21 | 0.14 | 0.42 |
| 2_D7 | WT (1) gRNA3-tm2-HDR (20) | 28 | 0.00 | 0.00 | 0.00 |
| 2_D8 | gRNA3-tm2-HDR | 2591 | 0.04 | 0.15 | 0.58 |
| 2_D9 | gRNA3-tm2-HDR | 15 | 0.00 | 0.00 | 0.00 |
| 2_E1 | gRNA3-tm2-HDR | 3303 | 0.09 | 0.27 | 0.45 |
| 2_E10 | gRNA2-HDR/NHEJ | 60 | 0.00 | 0.00 | 0.00 |
| 2_E2 | gRNA3-tm2-HDR | 2682 | 0.15 | 0.30 | 0.26 |
| 2_E3 | WT gRNA3-tm2-HDR | 2824 | 0.14 | 0.32 | 0.42 |
| 2_E4 | WT | 3115 | 0.26 | 0.22 | 0.32 |
| 2_E5 | gRNA3-tm2-HDR | 2802 | 0.07 | 0.21 | 5.03 |
| 2_E6 | gRNA3-tm2-HDR | 48 | 2.08 | 2.08 | 4.17 |
| 2_E7 | gRNA3-tm2-HDR | 65 | 0.00 | 0.00 | 6.15 |
| 2_E8 | gRNA3-tm2-HDR | 3182 | 0.06 | 0.25 | 4.56 |
| 2_E9 | gRNA3-tm2-HDR gRNA2-HDR/NHEJ | 3392 | 0.00 | 0.29 | 4.95 |
| 3_A_1 | WT | 2924 | 0.17 | 0.27 | 0.21 |
| 3_A_10 | gRNA1-HDR | 3387 | 51.52 | 0.21 | 0.24 |
| 3_A_11 | gRNA1-HDR | 2596 | 35.48 | 0.19 | 0.31 |
| 3_A_12 | gRNA1-HDR | 2474 | 51.17 | 0.20 | 0.53 |
| 3_A_13 | gRNA1-HDR | 3039 | 67.39 | 0.20 | 0.36 |
| 3_A_14 | gRNA1-HDR | 2661 | 69.41 | 0.30 | 0.19 |
| 3_A_15 | gRNA1-HDR | 15 | 53.33 | 0.00 | 0.00 |
| 3_A_16 | gRNA1-HDR | 17 | 23.53 | 0.00 | 0.00 |
| 3_A_17 | gRNA1-HDR gRNA2-HDR/NHEJ | 3280 | 17.74 | 14.82 | 0.43 |
| 3_A_18 | gRNA1-HDR gRNA2-HDR/NHEJ | 3313 | 19.98 | 11.02 | 0.36 |
| 3_A_19 | gRNA2-HDR/NHEJ | 8 | 0.00 | 12.50 | 0.00 |
| 3_A_2 | WT | 2518 | 0.08 | 0.04 | 0.44 |
| 3_A_20 | gRNA2-HDR/NHEJ | 3109 | 0.06 | 14.96 | 0.45 |
| 3_A_21 | gRNA2-HDR/NHEJ | 3126 | 1.22 | 22.17 | 0.22 |
| 3_A_3 | WT | 3324 | 0.12 | 0.24 | 0.48 |
| 3_A_4 | WT | 2958 | 0.17 | 0.20 | 0.34 |
| 3_A_5 | WT | 3543 | 0.11 | 0.20 | 0.34 |
| 3_A_6 | WT | 2829 | 0.04 | 0.11 | 0.67 |
| 3_A_7 | WT | 2770 | 0.18 | 0.22 | 0.32 |
| 3_A_8 | WT | 3813 | 0.08 | 0.24 | 0.29 |
| 3_A_9 | gRNA1-HDR (15) WT (6) | 2534 | 33.86 | 0.28 | 0.51 |
| 3_B1 | gRNA3-tm2-HDR | 8 | 0.00 | 0.00 | 0.00 |
| 3_B10 | gRNA2-HDR/NHEJ | 3720 | 0.05 | 4.33 | 0.62 |
| 3_B11 | gRNA2-HDR/NHEJ | 14 | 0.00 | 21.43 | 0.00 |
| 3_B12 | gRNA2-HDR/NHEJ / gRNA3-tm2-HDR | 2585 | 0.04 | 7.70 | 2.94 |
| 3_B13 | gRNA3-tm2-HDR | 3079 | 0.13 | 0.19 | 5.20 |
| 3_B14 | gRNA3-tm2-HDR | 9 | 0.00 | 11.11 | 0.00 |
| 3_B15 | gRNA3-tm2-HDR | 3 | 0.00 | 0.00 | 0.00 |
| 3_B16 | gRNA3-tm2-HDR | 11 | 0.00 | 0.00 | 0.00 |
| 3_B17 | gRNA3-tm2-HDR | 26 | 0.00 | 0.00 | 0.00 |
| 3_B18 | gRNA3-tm2-HDR | 6 | 0.00 | 0.00 | 0.00 |
| 3_B19 | gRNA3-tm2-HDR | 2039 | 0.05 | 0.15 | 6.87 |
| 3_B2 | gRNA3-tm2-HDR | 7 | 0.00 | 0.00 | 0.00 |
| 3_B20 | gRNA3-tm2-HDR | 13 | 0.00 | 0.00 | 7.69 |
| 3_B21 | gRNA3-tm2-HDR | 17 | 0.00 | 11.76 | 0.00 |
| 3_B3 | gRNA3-tm2-HDR | 3112 | 0.10 | 0.39 | 0.32 |
| 3_B4 | gRNA3-tm2-HDR | 3500 | 0.11 | 0.14 | 1.11 |
| 3_B5 | gRNA3-tm2-HDR | 4066 | 0.05 | 0.20 | 5.68 |
| 3_B6 | gRNA3-tm2-HDR | 8 | 0.00 | 0.00 | 0.00 |
| 3_B7 | gRNA3-tm2-HDR | 3674 | 0.05 | 0.24 | 3.97 |
| 3_B8 | gRNA3-tm2-HDR | 17 | 0.00 | 0.00 | 0.00 |
| 3_B9 | gRNA3-tm2-HDR gRNA2-HDR/NHEJ | 15 | 0.00 | 0.00 | 0.00 |
| 3_C1 | gRNA3-tm2-HDR | 3145 | 0.13 | 0.22 | 0.41 |
| 3_C10 | gRNA3-tm2-HDR | 2235 | 0.00 | 0.18 | 2.86 |
| 3_C11 | gRNA3-tm2-HDR | 3031 | 0.03 | 0.40 | 4.12 |
| 3_C12 | gRNA3-tm2-HDR | 2459 | 0.12 | 0.16 | 0.45 |
| 3_C13 | gRNA3-tm2-HDR gRNA2-HDR/NHEJ | 3709 | 0.08 | 5.90 | 0.54 |
| 3_C14 | gRNA3-tm2-HDR gRNA2-HDR/NHEJ | 2920 | 0.07 | 21.75 | 1.64 |
| 3_C15 | gRNA3-tm2-HDR | 2860 | 0.10 | 0.35 | 6.29 |
| 3_C16 | gRNA3-tm2-HDR | 3037 | 0.07 | 0.20 | 2.93 |
| 3_C17 | gRNA3-tm2-HDR | 2902 | 0.00 | 0.17 | 8.92 |
| 3_C18 | gRNA3-tm2-HDR | 3867 | 0.18 | 0.16 | 4.81 |
| 3_C19 | gRNA3-tm2-HDR | 3327 | 0.12 | 0.12 | 7.94 |
| 3_C2 | gRNA3-tm2-HDR | 3363 | 0.12 | 0.33 | 0.39 |
| 3_C20 | gRNA3-tm2-HDR | 3652 | 0.22 | 0.14 | 6.65 |
| 3_C21 | gRNA3-tm2-HDR | 3168 | 0.16 | 0.19 | 4.17 |
| 3_C3 | gRNA3-tm2-HDR | 3813 | 0.08 | 0.18 | 0.39 |
| 3_C4 | gRNA3-tm2-HDR | 3506 | 0.06 | 0.14 | 2.42 |
| 3_C5 | gRNA3-tm2-HDR | 3536 | 0.14 | 0.14 | 8.00 |
| 3_C6 | gRNA3-tm2-HDR | 2247 | 0.04 | 0.22 | 7.57 |
| 3_C7 | gRNA3-tm2-HDR | 2411 | 0.08 | 0.17 | 5.35 |
| 3_C8 | gRNA3-tm2-HDR | 3053 | 0.13 | 0.20 | 10.28 |
| 3_C9 | gRNA3-tm2-HDR | 3163 | 0.06 | 0.35 | 3.51 |
| 3_D1 | WT | 3048 | 0.10 | 0.30 | 0.46 |
| 3_D10 | WT | 2540 | 0.12 | 0.24 | 0.39 |
| 3_D11 | WT gRNA1-HDR | 1503 | 33.27 | 0.27 | 0.00 |
| 3_D12 | gRNA1-HDR | 2419 | 68.00 | 0.17 | 0.45 |
| 3_D13 | gRNA1-HDR | 3195 | 73.40 | 0.16 | 0.38 |
| 3_D14 | gRNA1-HDR | 2000 | 53.65 | 0.30 | 0.35 |
| 3_D15 | gRNA1-HDR | 2949 | 63.55 | 0.20 | 0.41 |
| 3_D16 | gRNA1-HDR | 3288 | 66.51 | 0.24 | 0.33 |
| 3_D17 | gRNA1-HDR | 1936 | 69.01 | 0.15 | 0.62 |
| 3_D18 | gRNA1-HDR | 2798 | 68.83 | 0.29 | 0.43 |
| 3_D19 | gRNA1-HDR gRNA2-HDR/NHEJ | 2264 | 13.91 | 17.23 | 0.18 |
| 3_D2 | WT | 2807 | 0.11 | 0.21 | 0.18 |
| 3_D20 | gRNA2-HDR/NHEJ | 2436 | 0.21 | 29.06 | 0.25 |
| 3_D21 | gRNA2-HDR/NHEJ | 2245 | 0.09 | 37.82 | 0.49 |
| 3_D3 | WT | 2835 | 0.21 | 0.11 | 0.21 |
| 3_D4 | WT | 3032 | 0.10 | 0.20 | 0.56 |
| 3_D5 | WT | 1327 | 0.15 | 0.08 | 0.45 |
| 3_D6 | WT | 2582 | 0.04 | 0.27 | 0.43 |
| 3_D7 | WT | 1480 | 0.00 | 0.07 | 0.27 |
| 3_D8 | WT | 2216 | 0.05 | 0.05 | 0.18 |
| 3_D9 | WT | 3074 | 0.20 | 0.20 | 0.52 |
| 3_E1 | gRNA2-HDR/NHEJ | 2194 | 0.14 | 18.55 | 0.50 |
| 3_E10 | gRNA3-tm2-HDR | 52 | 0.00 | 0.00 | 11.54 |
| 3_E2 | gRNA2-HDR/NHEJ WT | 2444 | 0.25 | 16.65 | 0.41 |
| 3_E3 | gRNA2-HDR/NHEJ | 2239 | 0.18 | 33.77 | 0.45 |
| 3_E4 | gRNA2-HDR/NHEJ | 2578 | 0.19 | 26.84 | 0.43 |
| 3_E5 | gRNA1-HDR | 2177 | 25.13 | 5.24 | 0.23 |
| 3_E6 | gRNA1-HDR | 2496 | 40.18 | 0.20 | 0.56 |
| 3_E7 | gRNA1-HDR | 2410 | 42.49 | 0.25 | 0.46 |
| 3_E8 | gRNA1-HDR | 2416 | 44.66 | 0.41 | 0.37 |
| 3_E9 | gRNA1-HDR | 1837 | 37.18 | 0.05 | 0.38 |

Note*: The Column Pool Name is coded to convey the sample location coordinates:

First Number (1, 2, or 3): Indicates the originating table (Table 1, Table 2, or Table 3).

Letter (A–E): Represents the block number within the table (Block 1 to Block 5).

Number (1–21): Denotes the pool number within the block (Pool 1 to Pool 21).

*For example, "1_A1" refers to a sample from Table 1, Block A (Block 1), Pool 1.*

**Supplementary Table 9.** Chimeric patterns identified in specific pools with corresponding read depths and haplotype strings.

| **Pool Name*** | **Pattern Depth (Reads)** | **Haplotype String**** |
| --- | --- | --- |
| 1_d_10 | 6 | BBBBBBBBBBBBBBBBBBBBBBBBBBBBBBBBBBBBBBBBBBBBBBBBBBBBBBBBBBBBMM |
| 1_d_15 | 4 | BBBBBBBBBBBBBBBBBBBBBBBBBBBBBBBBBBBBBBBBBBBBBBBBBBBBBBBBBBBBMM |
| 1_d_7 | 4 | BBBBBBBBBBBBBBBBBBBBBBBBBBBBBBBBBBBBBBBBBBBBBBBBBBBBBBBBBBBBMM |
| 1_d_8 | 5 | BBBBBBBBBBBBBBBBBBBBBBBBBBBBBBBBBBBBBBBBBBBBBBBBBBBBBBBBBBBBMM |
| 1_d_10 | 4 | BBBBBBBBBBBBBBBBBBBBBBBBBBBBBBBBBBBBBBBBBBBBBBBBBBBBBBBBBBMMMM |
| 1_d_8 | 3 | BBBBBBBBBBBBBBBBBBBBBBBBBBBBBBBBBBBBBBBBBBBBBBBBBBBBBBBBBBMMMM |
| 1_d_9 | 3 | BBBBBBBBBBBBBBBBBBBBBBBBBBBBBBBBBBBBBBBBBBBBBBBBBBBBBBBBBBMMMM |
| 1_d_10 | 5 | BBBBBBBBBBBBBBBBBBBBBBBMMMMMMMMMMMMMMMMMMMMMMMMMMMMMMMMMMMMMMM |
| 1_d_15 | 8 | BBBBBBBBBBBBBBBBBBBBBBBMMMMMMMMMMMMMMMMMMMMMMMMMMMMMMMMMMMMMMM |
| 1_d_10 | 7 | BBBBBBBBBBBBBBBBBBBBBBMMMMMMMMMMMMMMMMMMMMMMMMMMMMMMMMMMMMMMMM |
| 1_d_15 | 8 | BBBBBBBBBBBBBBBBBBBBBBMMMMMMMMMMMMMMMMMMMMMMMMMMMMMMMMMMMMMMMM |
| 1_d_8 | 3 | BBBBBBBBBBBBBBBBBBBBBBMMMMMMMMMMMMMMMMMMMMMMMMMMMMMMMMMMMMMMMM |
| 1_d_9 | 4 | BBBBBBBBBBBBBBBBBBBBBBMMMMMMMMMMMMMMMMMMMMMMMMMMMMMMMMMMMMMMMM |
| 1_d_10 | 4 | BBBBBBBBBBBBBBBBBBBBBMMMMMMMMMMMMMMMMMMMMMMMMMMMMMMMMMMMMMMMMM |
| 1_d_15 | 6 | BBBBBBBBBBBBBBBBBBBBBMMMMMMMMMMMMMMMMMMMMMMMMMMMMMMMMMMMMMMMMM |
| 1_d_10 | 3 | BBBBBBBBBBBBBBBBBBBBMMMMMMMMMMMMMMMMMMMMMMMMMMMMMMMMMMMMMMMMMM |
| 1_d_10 | 3 | BBBBBBBBBBBBBBBBBBBMMMMMMMMMMMMMMMMMMMMMMMMMMMMMMMMMMMMMMMMMMM |
| 1_d_15 | 3 | BBBBBBBBBBBBBBBBBBBMMMMMMMMMMMMMMMMMMMMMMMMMMMMMMMMMMMMMMMMMMM |
| 1_d_10 | 3 | BBBBBBBBBBBBBMMMMMMMMMMMMMMMMMMMMMMMMMMMMMMMMMMMMMMMMMMMMMMMMM |
| 1_d_10 | 3 | BBBBBBBBBBBBMMMMMMMMMMMMMMMMMMMMMMMMMMMMMMMMMMMMMMMMMMMMMMMMMM |
| 1_d_10 | 4 | BBBBBBBBBBMMMMMMMMMMMMMMMMMMMMMMMMMMMMMMMMMMMMMMMMMMMMMMMMMMMM |
| 1_d_15 | 4 | BBBBBBBBBBMMMMMMMMMMMMMMMMMMMMMMMMMMMMMMMMMMMMMMMMMMMMMMMMMMMM |
| 1_d_10 | 10 | BBBBBBBBBMMMMMMMMMMMMMMMMMMMMMMMMMMMMMMMMMMMMMMMMMMMMMMMMMMMMM |
| 1_d_15 | 12 | BBBBBBBBBMMMMMMMMMMMMMMMMMMMMMMMMMMMMMMMMMMMMMMMMMMMMMMMMMMMMM |
| 1_d_9 | 5 | BBBBBBBBBMMMMMMMMMMMMMMMMMMMMMMMMMMMMMMMMMMMMMMMMMMMMMMMMMMMMM |
| 1_d_15 | 3 | BBBBBBBBMMMMMMMMMMMMMMMMMMMMMMMMMMMMMMMMMMMMMMMMMMMMMMMMMMMMMM |
| 1_d_10 | 5 | BBBBBBBMMMMMMMMMMMMMMMMMMMMMMMMMMMMMMMMMMMMMMMMMMMMMMMMMMMMMMM |
| 1_d_15 | 4 | BBBBBBBMMMMMMMMMMMMMMMMMMMMMMMMMMMMMMMMMMMMMMMMMMMMMMMMMMMMMMM |
| 1_d_9 | 4 | BBBBBBBMMMMMMMMMMMMMMMMMMMMMMMMMMMMMMMMMMMMMMMMMMMMMMMMMMMMMMM |
| 1_d_10 | 4 | BBBBBMMMMMMMMMMMMMMMMMMMMMMMMMMMMMMMMMMMMMMMMMMMMMMMMMMMMMMMMM |
| 1_d_15 | 5 | BBBBBMMMMMMMMMMMMMMMMMMMMMMMMMMMMMMMMMMMMMMMMMMMMMMMMMMMMMMMMM |
| 1_d_8 | 3 | BBBBBMMMMMMMMMMMMMMMMMMMMMMMMMMMMMMMMMMMMMMMMMMMMMMMMMMMMMMMMM |
| 1_d_9 | 6 | BBBBBMMMMMMMMMMMMMMMMMMMMMMMMMMMMMMMMMMMMMMMMMMMMMMMMMMMMMMMMM |
| 1_d_10 | 4 | BBBBMMMMMMMMMMMMMMMMMMMMMMMMMMMMMMMMMMMMMMMMMMMMMMMMMMMMMMMMMM |
| 1_d_11 | 3 | BBBBMMMMMMMMMMMMMMMMMMMMMMMMMMMMMMMMMMMMMMMMMMMMMMMMMMMMMMMMMM |
| 1_d_15 | 7 | BBBBMMMMMMMMMMMMMMMMMMMMMMMMMMMMMMMMMMMMMMMMMMMMMMMMMMMMMMMMMM |
| 1_d_9 | 4 | BBBBMMMMMMMMMMMMMMMMMMMMMMMMMMMMMMMMMMMMMMMMMMMMMMMMMMMMMMMMMM |
| 1_d_10 | 8 | BBBMMMMMMMMMMMMMMMMMMMMMMMMMMMMMMMMMMMMMMMMMMMMMMMMMMMMMMMMMMM |
| 1_d_15 | 9 | BBBMMMMMMMMMMMMMMMMMMMMMMMMMMMMMMMMMMMMMMMMMMMMMMMMMMMMMMMMMMM |
| 1_d_8 | 6 | BBBMMMMMMMMMMMMMMMMMMMMMMMMMMMMMMMMMMMMMMMMMMMMMMMMMMMMMMMMMMM |
| 1_d_9 | 7 | BBBMMMMMMMMMMMMMMMMMMMMMMMMMMMMMMMMMMMMMMMMMMMMMMMMMMMMMMMMMMM |
| 1_d_15 | 3 | BBMMMMMMMMMMMMMMMMMMMMMMMMMMMMMMMMMMMMMMMMMMMMMMMMMMMMMMMMMMMM |
| 1_d_10 | 13 | MMMBBBBBBBBBBBBBBBBBBBBBBBBBBBBBBBBBBBBBBBBBBBBBBBBBBBBBBBBBBB |
| 1_d_15 | 7 | MMMBBBBBBBBBBBBBBBBBBBBBBBBBBBBBBBBBBBBBBBBBBBBBBBBBBBBBBBBBBB |
| 1_d_7 | 5 | MMMBBBBBBBBBBBBBBBBBBBBBBBBBBBBBBBBBBBBBBBBBBBBBBBBBBBBBBBBBBB |
| 1_d_8 | 5 | MMMBBBBBBBBBBBBBBBBBBBBBBBBBBBBBBBBBBBBBBBBBBBBBBBBBBBBBBBBBBB |
| 1_d_10 | 3 | MMMMBBBBBBBBBBBBBBBBBBBBBBBBBBBBBBBBBBBBBBBBBBBBBBBBBBBBBBBBBB |
| 1_d_10 | 4 | MMMMMBBBBBBBBBBBBBBBBBBBBBBBBBBBBBBBBBBBBBBBBBBBBBBBBBBBBBBBBB |
| 1_d_9 | 4 | MMMMMBBBBBBBBBBBBBBBBBBBBBBBBBBBBBBBBBBBBBBBBBBBBBBBBBBBBBBBBB |
| 1_d_10 | 5 | MMMMMMMBBBBBBBBBBBBBBBBBBBBBBBBBBBBBBBBBBBBBBBBBBBBBBBBBBBBBBB |
| 1_d_15 | 3 | MMMMMMMBBBBBBBBBBBBBBBBBBBBBBBBBBBBBBBBBBBBBBBBBBBBBBBBBBBBBBB |
| 1_d_9 | 3 | MMMMMMMBBBBBBBBBBBBBBBBBBBBBBBBBBBBBBBBBBBBBBBBBBBBBBBBBBBBBBB |
| 1_d_10 | 14 | MMMMMMMMMBBBBBBBBBBBBBBBBBBBBBBBBBBBBBBBBBBBBBBBBBBBBBBBBBBBBB |
| 1_d_15 | 8 | MMMMMMMMMBBBBBBBBBBBBBBBBBBBBBBBBBBBBBBBBBBBBBBBBBBBBBBBBBBBBB |
| 1_d_10 | 3 | MMMMMMMMMMBBBBBBBBBBBBBBBBBBBBBBBBBBBBBBBBBBBBBBBBBBBBBBBBBBBB |
| 1_d_15 | 3 | MMMMMMMMMMBBBBBBBBBBBBBBBBBBBBBBBBBBBBBBBBBBBBBBBBBBBBBBBBBBBB |
| 1_d_15 | 3 | MMMMMMMMMMMMBBBBBBBBBBBBBBBBBBBBBBBBBBBBBBBBBBBBBBBBBBBBBBBBBB |
| 1_d_10 | 4 | MMMMMMMMMMMMMBBBBBBBBBBBBBBBBBBBBBBBBBBBBBBBBBBBBBBBBBBBBBBBBB |
| 1_d_15 | 4 | MMMMMMMMMMMMMBBBBBBBBBBBBBBBBBBBBBBBBBBBBBBBBBBBBBBBBBBBBBBBBB |
| 1_d_15 | 4 | MMMMMMMMMMMMMMMMMMMMBBBBBBBBBBBBBBBBBBBBBBBBBBBBBBBBBBBBBBBBBB |
| 1_d_9 | 3 | MMMMMMMMMMMMMMMMMMMMBBBBBBBBBBBBBBBBBBBBBBBBBBBBBBBBBBBBBBBBBB |
| 1_d_10 | 3 | MMMMMMMMMMMMMMMMMMMMMBBBBBBBBBBBBBBBBBBBBBBBBBBBBBBBBBBBBBBBBB |
| 1_d_10 | 4 | MMMMMMMMMMMMMMMMMMMMMMBBBBBBBBBBBBBBBBBBBBBBBBBBBBBBBBBBBBBBBB |
| 1_d_15 | 5 | MMMMMMMMMMMMMMMMMMMMMMBBBBBBBBBBBBBBBBBBBBBBBBBBBBBBBBBBBBBBBB |
| 1_d_10 | 3 | MMMMMMMMMMMMMMMMMMMMMMMBBBBBBBBBBBBBBBBBBBBBBBBBBBBBBBBBBBBBBB |
| 1_d_15 | 5 | MMMMMMMMMMMMMMMMMMMMMMMBBBBBBBBBBBBBBBBBBBBBBBBBBBBBBBBBBBBBBB |
| 1_d_8 | 3 | MMMMMMMMMMMMMMMMMMMMMMMMMMMMMMMMMMMMMMMMMMMMMMMMMMMMMMMMMMBBBB |
| 1_d_9 | 3 | MMMMMMMMMMMMMMMMMMMMMMMMMMMMMMMMMMMMMMMMMMMMMMMMMMMMMMMMMMBBBB |
| 1_d_15 | 3 | MMMMMMMMMMMMMMMMMMMMMMMMMMMMMMMMMMMMMMMMMMMMMMMMMMMMMMMMMMMBBB |
| 1_d_10 | 3 | MMMMMMMMMMMMMMMMMMMMMMMMMMMMMMMMMMMMMMMMMMMMMMMMMMMMMMMMMMMMBB |
| 1_d_9 | 4 | MMMMMMMMMMMMMMMMMMMMMMMMMMMMMMMMMMMMMMMMMMMMMMMMMMMMMMMMMMMMBB |
| 1_4_6 | 3 | BBBBBBBBBBBBBMMMMMMMMMMMMMMMMMMMMMMMMMMMMMMMMMMMMMMMMMMMMMMMMM |
| 1_4_6 | 3 | BBBBBBBBBMMMMMMMMMMMMMMMMMMMMMMMMMMMMMMMMMMMMMMMMMMMMMMMMMMMMM |
| 1_4_2 | 4 | BBBBBBBMMMMMMMMMMMMMMMMMMMMMMMMMMMMMMMMMMMMMMMMMMMMMMMMMMMMMMM |
| 1_4_6 | 4 | BBBBBBBMMMMMMMMMMMMMMMMMMMMMMMMMMMMMMMMMMMMMMMMMMMMMMMMMMMMMMM |
| 1_4_6 | 3 | BBBMMMMMMMMMMMMMMMMMMMMMMMMMMMMMMMMMMMMMMMMMMMMMMMMMMMMMMMMMMM |
| 1_4_6 | 7 | MMMMMBBBBBBBBBBBBBBBBBBBBBBBBBBBBBBBBBBBBBBBBBBBBBBBBBBBBBBBBB |
| 1_4_2 | 7 | MMMMMBBBBBBBBBBBBBBBBBBBBBBBBBBBBBBBBBBBBBBBBBBBBBBBBBBBBBBBBB |
| 1_4_6 | 3 | MMMMMMMBBBBBBBBBBBBBBBBBBBBBBBBBBBBBBBBBBBBBBBBBBBBBBBBBBBBBBB |
| 1_4_2 | 3 | MMMMMMMBBBBBBBBBBBBBBBBBBBBBBBBBBBBBBBBBBBBBBBBBBBBBBBBBBBBBBB |
| 1_4_2 | 3 | MMMMMMMMMBBBBBBBBBBBBBBBBBBBBBBBBBBBBBBBBBBBBBBBBBBBBBBBBBBBBB |
| 1_4_6 | 6 | MMMMMMMMMBBBBBBBBBBBBBBBBBBBBBBBBBBBBBBBBBBBBBBBBBBBBBBBBBBBBB |
| 1_4_6 | 3 | MMMMMMMMMMBBBBBBBBBBBBBBBBBBBBBBBBBBBBBBBBBBBBBBBBBBBBBBBBBBBB |
| 1_4_2 | 3 | MMMMMMMMMMBBBBBBBBBBBBBBBBBBBBBBBBBBBBBBBBBBBBBBBBBBBBBBBBBBBB |
| 1_4_2 | 3 | MMMMMMMMMMMMMMMMMMMMBBBBBBBBBBBBBBBBBBBBBBBBBBBBBBBBBBBBBBBBBB |
| 1_4_6 | 3 | MMMMMMMMMMMMMMMMMMMMBBBBBBBBBBBBBBBBBBBBBBBBBBBBBBBBBBBBBBBBBB |
| 1_4_2 | 3 | MMMMMMMMMMMMMMMMMMMMMBBBBBBBBBBBBBBBBBBBBBBBBBBBBBBBBBBBBBBBBB |
| 1_4_6 | 3 | MMMMMMMMMMMMMMMMMMMMMBBBBBBBBBBBBBBBBBBBBBBBBBBBBBBBBBBBBBBBBB |
| 1_4_2 | 3 | MMMMMMMMMMMMMMMMMMMMMMBBBBBBBBBBBBBBBBBBBBBBBBBBBBBBBBBBBBBBBB |
| 1_4_6 | 4 | MMMMMMMMMMMMMMMMMMMMMMBBBBBBBBBBBBBBBBBBBBBBBBBBBBBBBBBBBBBBBB |
| 1_4_2 | 4 | MMMMMMMMMMMMMMMMMMMMMMMBBBBBBBBBBBBBBBBBBBBBBBBBBBBBBBBBBBBBBB |
| 1_4_6 | 5 | MMMMMMMMMMMMMMMMMMMMMMMBBBBBBBBBBBBBBBBBBBBBBBBBBBBBBBBBBBBBBB |

NOTE*: The Pool Name column is coded to convey the sample location coordinates as follows:

First Number (1, 2, or 3): Indicates the originating table (Table 1, Table 2, or Table 3).

Second Number (1–5): Represents the block number within the table (Block 1 to Block 5).

Third Number (1–21): Denotes the pool number within the block (Pool 1 to Pool 21).

For example, "1_1_1" refers to a sample from Table 1, Block 1, Pool 1.

Note**: Haplotype string colors link SNP strings with complementary haplotype switches; for instance, "…MMMMBBBB…" and "…BBBBMMMM…" are displayed in the same color to indicate their complementary relationship.

**Supplementary Table 10.** Table listing the F1 seedling pools with putative PCR template switching artefacts.

| **Pool Name*** | **Total Chimeric Reads** | **Reads in Pool** | **PCR Template Switch Rate (%)** |
| --- | --- | --- | --- |
| 1_A18 | 3 | 3289 | 0.091 |
| 1_B10 | 3 | 3231 | 0.093 |
| 1_C13 | 3 | 7282 | 0.041 |
| 1_3_13 | 10 | 962 | 2.703 |
| 1_3_15 | 3 | 904 | 1.549 |
| 1_5_21 | 3 | 3858 | 0.078 |
| 2_1_14 | 3 | 2420 | 0.124 |
| 2_2_1 | 7 | 2643 | 0.265 |
| 2_3_10 | 3 | 3321 | 0.09 |
| 2_3_18 | 3 | 2889 | 0.104 |
| 2_D8 | 3 | 2591 | 0.116 |
| 2_4_3 | 3 | 2227 | 0.135 |
| 2_4_13 | 3 | 2065 | 0.145 |
| 3_3_9 | 3 | 2253 | 0.133 |
| 3_3_7 | 3 | 2876 | 0.104 |
| 3_4_16 | 3 | 2296 | 0.131 |

NOTE*: The Pool Name column is coded to convey the sample location coordinates as follows:

First Number (1, 2, or 3): Indicates the originating table (Table 1, Table 2, or Table 3).

Second Number (1–5): Represents the block number within the table (Block 1 to Block 5).

Third Number (1–21): Denotes the pool number within the block (Pool 1 to Pool 21).

For example, "1_1_1" refers to a sample from Table 1, Block 1, Pool 1.

Note**: Haplotype string colors link SNP strings with complementary haplotype switches; for instance, "…MMMMBBBB…" and "…BBBBMMMM…" are displayed in the same color to indicate their complementary relationship.

**Supplementary Table 11.** Table of chimeric patterns detected in ONT sequencing data of F1 plants that match patterns found in seedling corresponding pool F1 pools.

| **Plant Information** | **Sequence** | **Read Depth** | **Total reads** | **Control** |  |
| --- | --- | --- | --- | --- | --- |
| gRNA3-tm2-HDR_Plant_2 | MMMMMMMMMMMMMMMMMMMMBBBBBBBBBBBBBBBBBBBBBBBBBBBBBBBBBBBBBBBBBB | 10 | 14841 | no |  |
| gRNA3-tm2-HDR_Plant_2 | MMMMMMMMMBBBBBBBBBBBBBBBBBBBBBBBBBBBBBBBBBBBBBBBBBBBBBBBBBBBBB | 21 |  | no |  |
| gRNA3-tm2-HDR_Plant_2 | MMMMMMMMMMMMMMMMMMMMMMBBBBBBBBBBBBBBBBBBBBBBBBBBBBBBBBBBBBBBBB | 27 |  | no |  |
| gRNA3-tm2-HDR_Plant_2 | MMMMMMMBBBBBBBBBBBBBBBBBBBBBBBBBBBBBBBBBBBBBBBBBBBBBBBBBBBBBBB | 31 |  | no |  |
| gRNA3-tm2-HDR_Plant_2 | MMMMMMMMMMMMMMMMMMMMMMMBBBBBBBBBBBBBBBBBBBBBBBBBBBBBBBBBBBBBBB | 31 |  | no |  |
| gRNA3-tm2-HDR_Plant_2 | BBBBBBBMMMMMMMMMMMMMMMMMMMMMMMMMMMMMMMMMMMMMMMMMMMMMMMMMMMMMMM | 34 |  | no |  |
| gRNA3-tm2-HDR_Plant_2 | BBBMMMMMMMMMMMMMMMMMMMMMMMMMMMMMMMMMMMMMMMMMMMMMMMMMMMMMMMMMMM | 37 |  | no |  |
| gRNA3-tm2-HDR_Plant_2 | BBBBBBBBBMMMMMMMMMMMMMMMMMMMMMMMMMMMMMMMMMMMMMMMMMMMMMMMMMMMMM | 38 |  | no |  |
| WT_MBxMM | MMMMMMMMMMMMMMMMMMMMBBBBBBBBBBBBBBBBBBBBBBBBBBBBBBBBBBBBBBBBBB | 16 | 23195 | yes |  |
| WT_MBxMM | MMMMMMMBBBBBBBBBBBBBBBBBBBBBBBBBBBBBBBBBBBBBBBBBBBBBBBBBBBBBBB | 19 |  | yes |  |
| WT_MBxMM | MMMMMMMMMMMMMMMMMMMMMBBBBBBBBBBBBBBBBBBBBBBBBBBBBBBBBBBBBBBBBB | 24 |  | yes |  |

**Supplementary Table 12.** Multiple comparison of means (tukey hsd) for assessing targeted recombination patterns in transfected protoplast pools across gRNA- and control groups.

| **group1** | **group2** | **meandiff** | **p-adj** | **lower** | **upper** | **reject** |
| --- | --- | --- | --- | --- | --- | --- |
| gRNA-control | gRNA1-HDR/NHEJ | -0.0229 | 0.9953 | -0.1262 | 0.0804 | FALSE |
| gRNA-control | gRNA2-HDR/NHEJ | -0.046 | 0.8217 | -0.1493 | 0.0574 | FALSE |
| gRNA-control | gRNA3-tm2-HDR | -0.0437 | 0.8556 | -0.147 | 0.0597 | FALSE |
| gRNA-control | gRNA4-tm2-HDR | 0.0577 | 0.6053 | -0.0456 | 0.161 | FALSE |
| gRNA-control | gRNA5-HDR/NHEJ | -0.0257 | 0.9906 | -0.1291 | 0.0776 | FALSE |
| gRNA-control | gRNA6-HDR/NHEJ | 0.0215 | 0.9969 | -0.0819 | 0.1248 | FALSE |
| gRNA-control | gRNA7-HDR/NHEJ | 0.0555 | 0.6502 | -0.0479 | 0.1588 | FALSE |
| gRNA1-HDR/NHEJ | gRNA2-HDR/NHEJ | -0.023 | 0.9977 | -0.1396 | 0.0935 | FALSE |
| gRNA1-HDR/NHEJ | gRNA3-tm2-HDR | -0.0208 | 0.9988 | -0.1373 | 0.0958 | FALSE |
| gRNA1-HDR/NHEJ | gRNA4-tm2-HDR | 0.0806 | 0.346 | -0.0359 | 0.1972 | FALSE |
| gRNA1-HDR/NHEJ | gRNA5-HDR/NHEJ | -0.0028 | 1 | -0.1194 | 0.1137 | FALSE |
| gRNA1-HDR/NHEJ | gRNA6-HDR/NHEJ | 0.0444 | 0.9095 | -0.0722 | 0.1609 | FALSE |
| gRNA1-HDR/NHEJ | gRNA7-HDR/NHEJ | 0.0784 | 0.3801 | -0.0382 | 0.1949 | FALSE |
| gRNA2-HDR/NHEJ | gRNA3-tm2-HDR | 0.0023 | 1 | -0.1143 | 0.1188 | FALSE |
| gRNA2-HDR/NHEJ | gRNA4-tm2-HDR | 0.1037 | 0.1084 | -0.0129 | 0.2202 | FALSE |
| gRNA2-HDR/NHEJ | gRNA5-HDR/NHEJ | 0.0202 | 0.999 | -0.0964 | 0.1368 | FALSE |
| gRNA2-HDR/NHEJ | gRNA6-HDR/NHEJ | 0.0674 | 0.5644 | -0.0491 | 0.184 | FALSE |
| gRNA2-HDR/NHEJ | gRNA7-HDR/NHEJ | 0.1014 | 0.1232 | -0.0152 | 0.218 | FALSE |
| gRNA3-tm2-HDR | gRNA4-tm2-HDR | 0.1014 | 0.1234 | -0.0152 | 0.218 | FALSE |
| gRNA3-tm2-HDR | gRNA5-HDR/NHEJ | 0.0179 | 0.9995 | -0.0986 | 0.1345 | FALSE |
| gRNA3-tm2-HDR | gRNA6-HDR/NHEJ | 0.0651 | 0.6048 | -0.0514 | 0.1817 | FALSE |
| gRNA3-tm2-HDR | gRNA7-HDR/NHEJ | 0.0991 | 0.1398 | -0.0174 | 0.2157 | FALSE |
| gRNA4-tm2-HDR | gRNA5-HDR/NHEJ | -0.0835 | 0.3056 | -0.2 | 0.0331 | FALSE |
| gRNA4-tm2-HDR | gRNA6-HDR/NHEJ | -0.0362 | 0.9672 | -0.1528 | 0.0803 | FALSE |
| gRNA4-tm2-HDR | gRNA7-HDR/NHEJ | -0.0023 | 1 | -0.1188 | 0.1143 | FALSE |
| gRNA5-HDR/NHEJ | gRNA6-HDR/NHEJ | 0.0472 | 0.8799 | -0.0693 | 0.1638 | FALSE |
| gRNA5-HDR/NHEJ | gRNA7-HDR/NHEJ | 0.0812 | 0.3374 | -0.0354 | 0.1978 | FALSE |
| gRNA6-HDR/NHEJ | gRNA7-HDR/NHEJ | 0.034 | 0.9769 | -0.0826 | 0.1505 | FALSE |

# References

van Rengs, W. M., Schmidt, M. H. W., Effgen, S., Le, D. B., Wang, Y., Zaidan, M. W. A. M., ... & Underwood, C. J. (2022). A chromosome scale tomato genome built from complementary PacBio and Nanopore sequences alone reveals extensive linkage drag during breeding. The Plant Journal, 110(2), 572-588.

Schouten, H. J., Tikunov, Y., Verkerke, W., Finkers, R., Bovy, A., Bai, Y., & Visser, R. G. (2019). Breeding has increased the diversity of cultivated tomato in the Netherlands. Frontiers in plant science, 10, 1606.

Víquez-Zamora, M., Caro, M., Finkers, R., Tikunov, Y., Bovy, A., Visser, R. G., ... & van Heusden, S. (2014). Mapping in the era of sequencing: high density genotyping and its application for mapping TYLCV resistance in Solanum pimpinellifolium. BMC genomics, 15, 1-10.
